# Supplementary material for: Retrospective observational study of emergency department syndromic surveillance data during air pollution episodes across London and Paris in 2014
Source: BMJ Open. 2018 Apr 19;8(4):e018732. doi: 10.1136/bmjopen-2017-018732 (PMC5914706; doi:10.1136/bmjopen-2017-018732)
Supplement: Author's manuscript [file bmjopen-2017-018732.draft_revisions.pdf]

BMJ Open

'A tale of two cities': a comparison of emergency department syndromic surveillance data during air pollution episodes across London and Paris in 2014

Journal:	*BMJ Open*
Manuscript ID	bmjopen-2017-018732
Article Type:	Research
Date Submitted by the Author:	19-Jul-2017
Complete List of Authors:	Hughes, Helen; Public Health England, Real-time Syndromic Surveillance; The Farr Institute @ HeRC,<br>Morbey, Roger; Public Health England, Real-time Syndromic Surveillance Team<br>Fouillet, Anne; Sante publique France, Syndromic Surveillance Unit<br>Caserio-Schönemann, Céline; Sante publique France, Syndromic Surveillance Unit<br>Dobney, Alec; Public Health England, Environmental Hazards and Emergencies Department<br>Hughes, Thomas; John Radcliffe Hospital, Emergency Department; Royal College of Emergency Medicine<br>Smith, Gillian; Public Health England, Real-time Syndromic Surveillance Team<br>Elliot, Alex; Health Protection Agency, Real-time Syndromic Surveillance Team
**Primary Subject Heading**:	Public health
Secondary Subject Heading:	Emergency medicine, Global health, Epidemiology
Keywords:	syndromic surveillance, air quality, particulate matter, emergency department, Asthma < THORACIC MEDICINE

SCHOLARONE™
Manuscripts

For peer review only - http://bmjopen.bmj.com/site/about/guidelines.xhtml

'A tale of two cities': a comparison of emergency department syndromic surveillance data during air pollution episodes across London and Paris in 2014

H. E. Hughes[1,2,], R. Morbey[1], A. Fouillet[3], C. Caserio-Schönemann[3], A Dobney[4], T. C. Hughes[5,6], G. E. Smith[1], and A. J. Elliot[1]

1 Real-time Syndromic Surveillance Team, National Infection Service, Public Health England, Birmingham, UK;

2 Farr Institute at HeRC, Liverpool, UK

3 Syndromic Surveillance Unit, Santé publique France, the national public health agency, Saint-Maurice, France;

4 Environmental Hazards and Emergencies Department, Centre for Radiation, Chemical and Environmental Hazards, Public Health England, Birmingham, UK;

5 John Radcliffe Hospital, Oxford, UK;

6 The Royal College of Emergency Medicine, London, UK.

Address for correspondence:

Helen E Hughes, Real-time Syndromic Surveillance Team, Public Health England, 6th Floor, 5 St Philip's Place, Birmingham B3 2PW, UK

email: h.hughes7@liverpool.ac.uk

phone: +44 (0)844 225 3560 option 4 > option 2; fax: +44 (0)121 236 2215

Reprints will not be available from the author.

Word count: 3613

Key words: Syndromic surveillance, Emergency Department, Air quality, particulate matter, asthma, London, Paris

For peer review only

Abstract

Introduction

Poor air quality (AQ) is a global public health issue and AQ events can span across countries. Using emergency department (ED) syndromic surveillance from England and France, we describe changes in human health indicators during periods of particularly poor AQ in London and Paris during 2014.

Methods

Using daily AQ data for 2014, we identified 3 periods of poor AQ affecting both London and Paris. Anonymised near real-time ED attendance syndromic surveillance data from EDs across England and France were used to monitor the health impact of poor AQ.

Using the routine English syndromic surveillance detection methods, increases in selected ED syndromic indicators (asthma, difficulty breathing and myocardial ischaemia), in total and by age, were identified and compared to periods of poor AQ in each city. Retrospective Wilcoxon-Mann-Witney tests were used to identify significant increases in ED attendance data on days with (and up to 3 days following) poor AQ.

Results

Almost 1.5 million ED attendances were recorded during the study period (27/2/14-1/10/14). Significant increases in ED attendances for asthma were identified around periods of poor AQ in both cities, especially in children (0-14yrs). Some variation was seen in Paris with a rapid increase during the first AQ period in asthma attendances amongst children (0-14yrs), whereas during the second period the increase was greater in adults.

Discussion

For peer review only

This work demonstrates the public health value of real-time syndromic surveillance in response to air pollution incidents, and the potential for further cross-border harmonisation to provide Europe-wide early alerting to health impacts.

Strengths and limitations of this study

- Routinely collected syndromic surveillance data from both England (London) and France (Paris) were analysed using similar health indicators
- A single statistical method, designed specifically for daily syndromic surveillance, was applied to data from both cities
- Air quality measurements were standardised across both cities, to overcome differences in the standard reporting from each
- Pollutants other than particulate matter were not included, though they may be responsible for impacts on human health
- We could not control for the potential effects of health warnings and media coverage on health care seeking behaviour

For peer review only

Introduction

Air quality

Air pollution has negative impacts on human health. Short term exposure to poor air quality can affect lung function, including exacerbating asthma symptoms, and is associated with other acute deteriorations in respiratory and cardiovascular health [1]. Similar health effects have also been reported due to long term exposure, with exposure to ambient air pollution associated with lung cancer and chronic respiratory and cardiovascular conditions [1]. In addition to illness within the community and increased need for health care, air pollution is also associated with increased mortality, with an estimated 4.7% of deaths in the England attributed to air pollution [2] and 9% of deaths in France attributed to PM2.5 [3].

Air quality (AQ) monitoring identifies long term trends informing policy, provides evidence of meeting (or missing) statutory target levels and quantifies the impact of preventative measures [4, 5]. Daily AQ monitoring enables daily reporting of both actual and modelled AQ (predicting one or more days in advance), for whole countries and/or individual cities, as well as on a smaller scale around individual monitoring stations [6-8]. This information is increasingly easy to access through websites and apps and is often reported through the media, especially following formal health warnings [9].

Syndromic surveillance

Syndromic surveillance initially focussed on infectious diseases such as influenza but is increasingly being used for other non-infectious public health events. This type of surveillance uses real-time data from patient contacts with health care services (e.g. telephone helplines, general practice/ family doctors, or emergency departments). Patient contacts/ attendances are grouped by diagnoses/ symptoms creating syndromic indicators such as ‘respiratory’ or ‘gastrointestinal’, providing valuable information for public health action [10]. The use of emergency department (ED) data lends itself particularly well to the

For peer review only

syndromic surveillance of non-infectious public health events, with patients seeking attention for a range of acute conditions [11-13]. Previous investigation of periods of poor air quality, have shown associated increases in health seeking behaviour as evidenced by syndromic surveillance, particularly for asthma and/ or difficulty breathing and heart failure[14-16], though not for myocardial infarction [16].

Aims

During March and early April 2014 there was a period of widespread poor AQ across Europe. In particular, the urban conurbations of London (England) and Paris (France) were affected by high temperatures, Saharan dust and industrial emissions, resulting in widespread media attention [17-19]. Here, we use routine emergency department (ED) syndromic surveillance data collected across London and Paris during poor AQ periods throughout 2014 to investigate the compatibility of the two countries' ED syndromic surveillance systems and estimate the public health impact and associated short-term changes in health care seeking behaviour for selected respiratory and cardiac syndromes across different age groups.

For peer review only

Methods

Air quality data

The area studied here has been limited to London and the whole Paris region (Île-de-France), rather than a country level. In England, the Department for Environment, Food and Rural Affairs monitors and reports on levels of air pollution using monitoring stations and provides health advice using the Daily Air Quality Index (DAQI) [9]. Air quality in the Paris region is monitored by Airparif and reported using the Citeair index [20].

Both DAQI and Citeair systems monitor and report on multiple pollutants, however each index is reported using different methodology. Therefore the daily pollution levels across both London and Paris were standardised here, using the reported levels of particulate matter ($PM_{2.5}$ and PM_{10}). The city wide average value for each PM on each calendar day was calculated as a mean of the maximum values reported for each monitoring station on that day, in that city [21, 22]. Periods of poor AQ were then defined as those when either the calculated $PM_{2.5}$ or PM_{10} average value corresponded to the DAQI index value of 'high', or 'very high' (index levels of 7-10: $PM_{2.5}$ >=54 or PM_{10} >=76). At these levels people, including those with no pre-existing medical conditions, are advised to consider reducing their activity levels, particularly outdoors [8].

Emergency department syndromic surveillance data

The Emergency Department Syndromic Surveillance System (EDSSS), is a sentinel ED system coordinated by Public Health England (PHE), collecting anonymised data from participating EDs on a daily basis [23]. EDs eligible for inclusion in this study were defined as those reporting using ICD-10 [24] or Snomed CT [25] diagnosis coding systems. This investigation included EDSSS participating EDs within the London PHE Centre, which all fall within central London.

For peer review only

The French national ED syndromic surveillance system collects daily data from the Organisation de la Surveillance COordonnée des URgences (OSCOUR®) network of EDs, coordinated by Santé Publique France [26]. All EDs reporting to OSCOUR® use ICD-10 for the coding of diagnoses. Aggregated, anonymised daily data for the Paris region were made available for this analysis.

Epidemiological analysis

Syndromic indicators were selected from the comparable indicators already created for each system, based on clinical knowledge and experience of the potential health effects linked to air pollution and those used in previous syndromic surveillance work: asthma, difficulty breathing and myocardial ischaemia (MI) (table 1). The syndromic surveillance indicators available were similar in both EDSSS and OSCOUR®, with minor differences only in non-asthma difficulty breathing type conditions (full details on the underlying clinical codes in each indicator are included in supplementary table 1).

Table 1: Syndromic surveillance indicators included in the EDSSS (London) and OSCOUR® (Paris) emergency department systems and used in the study

**EDSSS (London)**	**OSCOUR® (Paris)**	**Reported here as**
Asthma	Asthme	Asthma
Wheeze/ difficulty breathing	Dyspnée/ Insuffisance respiratoire	Difficulty breathing
Myocardial ischaemia	Ischémie myocardique	Myocardial Ischaemia (MI)

For each syndromic surveillance system, attendances were aggregated by age group defined as 0-14, 15-44, 45-64 and 65 years and over.

The epidemiological analysis of ED attendance data included construction of trends using the daily number of total (all cause) attendances with a diagnosis code within each surveillance system (denominator) and the number of attendances within an indicator (numerator). This

For peer review only

information was used to calculate the daily percentage of attendances for diagnoses mapped to each syndromic indicator, both for all ages and for each age group, and city.

Statistical analysis

The EDSSS and OSCOUR® are both live public health surveillance systems prospectively collecting data with automated contemporaneous statistical algorithms underpinning the detection of unusual activity. We applied the routine syndromic surveillance statistical detection algorithm from England: the RAMMIE method (Rising Activity, Multi-level Mixed effects Indicator Emphasis [27]). RAMMIE was applied to both English and French ED data, including to age specific data. Using RAMMIE two separate statistical thresholds were calculated: a 'historical' threshold (based on the previous 2 years of data) to identify significant activity compared to previous years, and a 'spike' threshold (based on the previous two weeks) to identify recent, statistically significant, increases in daily activity.

To ensure that sufficient data were included here to cover each of the AQ events identified, a study period of a minimum of 7 days pre the first and 7 days post the final period of poor AQ identified in London/ Paris during 2014 was selected. A further period of 2 years of data prior to the first AQ event provided required baseline data for the RAMMIE method.

In addition to the RAMMIE analysis, the Wilcoxon-Mann-Witney non-parametric test was used to test for significant differences in the syndromic indicators during the 2014 study period, by age group between those days with a poor AQ and those without. To allow for the possibility of a delayed response, separate analyses were conducted incorporating lags of one to three days following a day of poor AQ.

All analyses were undertaken using Stata v13.1[28].

For peer review only

Results

Air quality events

During 2014, several periods of poor AQ were identified where the 'high' or 'very high' air pollution thresholds for particulate matter ($PM_{2.5}$ and/or PM_{10}) had been breached in both London and Paris (supplementary figure 1). Periods of poor air quality in Paris were generally observed to be of a longer duration and with higher DAQI levels than in London, though more individual days of poor AQ were identified in London. Two main periods of poor AQ overlapped in these cities in mid-March (AQ1, the largest event in both locations and where transboundary dust from the Sahara contributed to the makeup of the particulate matter fraction [14]) and early April (AQ2, mainly in London, though a 1 day PM_{10} spike in Paris), with a third, less severe period during September occurring in both cities within a 7 day period (AQ3) (table 2).

Supplementary Figure 1: Calculated mean daily PM value and corresponding Daily Air Quality Index band, by day during 2014 in London a. $PM_{2.5}$, b. PM_{10}: Paris c. $PM_{2.5}$, d. PM_{10}.

An overall study period was defined as 27 February 2014 – 1 October 2014, to encompass each period where poor AQ occurred in both London and Paris, including 7 days before and after the first and final AQ events identified (table 2).

Table 2: Dates of poor air quality, coinciding in London and Paris during 2014

	AQ1	AQ2	AQ3
**London**	08/03/14 - 14/03/14	28/03/14 - 04/04/14	16/09/14 - 20/09/14
**Paris**	06/03/14 - 15/03/14	31/03/14	24/09/14

For peer review only

ED attendances

Over the study period 1,436,163 ED attendances were recorded across both London and Paris (table 3). Total attendances were higher in Paris (1,163,353; from 58 EDs) than London (272,810; from 5 EDs). A comparable level of diagnosis coding was included in each city with 79% of London attendances and 72% of Paris attendances including a clinical diagnosis code.

Table 3: Attendances recorded in EDs, by city, over the study period (27/02/14-01/10/14)

		ED Attendances		*Indicator attendances*		
**City**	**EDs**	**Total attendances**	**Diagnosis Coded**	**Asthma**	**Difficulty breathing**	**Myocardial ischaemia**
London	5*	272,810	214,730 (79%)	1,893 (0.9%)	812 (0.4%)	1,370 (0.6%)
Paris	58	1,163,353	840,309 (72%)	12,374 (1.5%)	5,433 (0.6%)	1,685 (0.2%)

*1 small ED stopped reporting to EDSSS on 10/09/2014. All 5 EDs were included in descriptive and RAMMIE analysis; 4 EDs that reported throughout were included in Wilcoxon-Mann-Whitney testing.

On a weekly basis, total ED attendances in both London and Paris showed similar trends, with a peak observed on a Monday. Examination of indicator trends illustrated that there were further similarities between EDSSS and OSCOUR® with highest levels of asthma attendances (as a percentage of attendances with a diagnosis code; and lowest levels of MI attendances, reported on Sundays (supplementary figure 2).

For peer review only

Supplementary Figure 2: Mean emergency department attendances by day of week, 27 February 2014 – 1 October 2014, by syndromic indicators, London reported to EDSSS (a,c,e) and Paris reported to OSCOUR® (b,d,f).

ED attendances during poor air quality periods

The application of RAMMIE generated statistical 'alarms', where the number of attendances observed was greater than threshold calculated from the baseline data ('historical' alarm; based on previous 2 years of data) and/or more recent trend data ('spike' alarm; based on previous 2 weeks). Overlaying these alarms on the daily percentage of attendances for each syndromic indicator and the periods of poor AQ showed where significantly higher than expected levels of ED attendances were observed (figures 1 & 2).

London ED attendances

Small increases in asthma attendances (all ages) in London EDs were observed following AQ1 (figure 1a). ED asthma attendances continued to increase during and immediately following AQ2. RAMMIE spike alarms were reported for the increases in asthma (all ages) immediately following AQ1 in London, indicating an attendance level higher than the previous 2 weeks. However, single day spike alarms were not unusual in this data and were also observed during periods with no reported AQ issues. Historical asthma alarms are less frequent and were not observed in these data during the study period.

Figure 1: Daily percentages of London ED attendances for syndromic surveillance indicators of A-B; asthma, C-D; difficulty breathing and E-F; myocardial ischaemia, selected age groups, with statistical alarms, reported to EDSSS.

The observed increase of asthma attendances during the AQ2 episode in London was most evident in children aged 0-14 years, and young adults (15-44 years) with each age group reaching a peak in attendances 1 to 2 days later (figure 1b). Asthma attendances for older adults showed no evidence of increase around periods of poor AQ (data not shown).

For peer review only

An additional peak in asthma (all ages) attendances was observed on 20/7/14 (figure 1a), particularly in children (0-14yrs; figure 1b), though there was no poor AQ identified at that time. During early September increases in all age attendances for asthma, largely driven by child attendances (0-14yrs), were observed to have started prior to AQ3.

A small increase in difficulty breathing attendances (all ages) immediately following AQ2 (figure 1c), was most apparent in the older adults (65+ years; figure 1d). This single day peak was the highest level seen in this age group, around double the usual level, though not significantly higher than historical data. Other age groups were not affected.

MI attendances were less common than asthma attendances in London EDs (table 3) and affected the adult age groups almost exclusively, as would be expected. Though a peak in MI attendances was observed during AQ2, particularly in those aged 65yrs+, a similar peak also occurred in late September, several days prior to the AQ3 (figure 1e & f).

Paris ED attendances

Clear increases in ED attendances (all ages) for asthma occurred during both AQ1 and AQ2 in Paris (figure 2a). These increases were detected by RAMMIE as statistically significant in comparison to previous years (historical alarm), as well as compared to the preceding 2 weeks (spike alarm). However, when broken down by age, the increase in asthma attendances in the 0-14 years age group occurred during AQ1, but not AQ2; while asthma attendances in young adults (15-44yrs) were greater during AQ2 than AQ1. No statistical alarms were observed for asthma in children around AQ2, though they were present for young adults (figure 2b).

The largest peak in asthma attendances was observed on 20/07/14, for all ages apart from 65yrs+ (data not shown), matching the spike seen in London, despite this not being a poor AQ period. One further peak in asthma attendances, apparent in all ages and individual age

For peer review only

groups, was observed on 9-10/6/14 (figure 2a & b). The observed peaks were not concomitant with any period of poor AQ in Paris, nor London.

Similar to London, an increase in asthma attendances was observed in Paris at the beginning of September, driven predominantly by children (0-14 years).

Figure 2: Daily percentages of Paris ED attendances for syndromic surveillance indicators of A-B; asthma, C-D; difficulty breathing and E-F; myocardial ischaemia, selected age groups, with statistical alarms, reported to OSCOUR®.

Difficulty breathing attendances in Paris were much lower than for asthma overall, with a single increase after AQ2 (figure 2c). Within the 15-44yrs age group there was, however an increase in difficulty breathing attendances following AQ1 (figure 4d).

Attendances for MI in Paris showed no evidence of increase in Paris during/ following days of poor AQ (figures 2e & f).

Retrospective statistical analysis

Wilcoxon-Mann-Whitney testing confirmed the descriptive epidemiology and RAMMIE results, with strong associations found between days of poor AQ and asthma attendances all ages and particularly in children 0-14yrs (supplementary table 2). The highest levels of statistical significance were identified following days of poor AQ: after 2 days in London and 3 days in Paris, which also showed an increase in older age groups. Asthma attendances 0-14yrs in London on the day of poor AQ showed a statistically significant increase, though not in Paris, where significant increases first occurred a day later.

Though there was some evidence of increased attendances for difficulty breathing and MI in selected age groups in London 1 day after poor AQ, these were single significant values (rather than the grouping of significant asthma results by age group). These were also not reflected in the Paris data.

For peer review only

Discussion

Main findings

We used two national ED syndromic surveillance systems to describe and compare the short-term changes in ED indicators during periods of poor AQ in two European capital cities. The AQ events reported here in Paris and London were related to the same pollutants ($PM_{2.5}$/ PM_{10}), and were very similar in terms of the dates and duration, and changes in public health outcomes in terms of ED attendances.

The most sensitive ED indicator during periods of poor AQ was asthma, with the impact most apparent up to 3 days after a day of poor AQ. The breakdown of attendances by age group revealed some differences, with the strongest associations overall seen between poor AQ and asthma attendances in children. This finding was consistent with previous studies which have shown children to be more susceptible to exacerbation of asthma symptoms requiring health care in association with air pollution [29].

The investigation of individual AQ incidents demonstrated the potential for differing levels of impact on different age groups at different times. Though generally children were most affected by AQ, a large increase in adult asthma attendances was observed during and immediately following AQ2 in both London and Paris. Within England this has previously been described [30]. As the second period of poor AQ to occur in a short period of time, media coverage and the associated communication of health warning information and interventions put in place during AQ2 may have resulted in changes in behaviour which affected the levels of exposure of different age groups.

In addition to the increases observed during AQ periods, a sharp increase in asthma attendances (all ages) was observed in Paris on 9-10/06/14, and in both London and Paris on 20/07/14. These peaks did not coincide with any AQ event identified here, however, additional meteorological data (not presented) revealed periods of major thunderstorm

For peer review only

activity within each city at the time [31-33]. These findings match those previously reported, including from the EDSSS, describing the health effects of 'thunderstorm asthma', where sudden exacerbation of asthma symptoms results in increased health care seeking behaviour over a short time period [13, 34-37], possibly due to increased levels of pollen and fungal spores, though the mechanism has not yet been confirmed[34].

We also observed further increases in asthma attendances in both Paris and London (and England and France as whole; data not shown) towards the start of September. This increase was particularly evident in children and is likely linked to an annual 'back to school' increase in asthma type attendances in EDs during September [38-40].

Other syndromic indicators investigated showed little (difficulty breathing), to no (MI) association with the AQ incidents identified here.

Strengths and limitations

The OSCOUR® system includes greater representative coverage nationally, with more EDs participating than the sentinel EDSSS system (540 EDs across France were reporting to OSCOUR® [41]. While 34 EDs across England and Northern Ireland were reporting to EDSSS at 20 March 2014, the five reported here were located in London making the EDSSS more representative in London than at the national level [42]). The large number of OSCOUR® EDs reported here resulted in much more stable data from Paris, reducing background noise and allowing clearer differentiation of spikes/increases in attendances. The smaller number of attendances within the EDSSS data made identifying spikes 'harder', however the use of RAMMIE enables significant increases in attendances to be identified, even when not initially obvious [27].

Despite underlying differences in the method of data collection, with EDSSS taking a single snapshot of daily attendances and OSCOUR® allowing the initial snapshot data to be updated retrospectively, both systems reported over 70% completion of the clinical diagnosis

For peer review only - http://bmjopen.bmj.com/site/about/guidelines.xhtml

field making diagnostic data comparable. Furthermore, though these systems were developed individually, it was found that the syndromic indicators used within each system were similar, making comparisons of health impact possible. However, the EDSSS used a wheeze/ difficulty breathing indicator whereas OSCOUR® used a difficulty breathing/ respiratory failure indicator. This is, in part, likely to be related to the use of different clinical coding systems, with the identification of symptoms (e.g. wheeze) more difficult using ICD-10 (as used in France) than Snomed-CT (used by some EDs in England).

The levels of attendances for each indicator were different between cities, with respiratory indicators higher in Paris (asthma 1.5%, difficulty breathing 0.7%), than London (asthma 0.9%, difficulty breathing 0.4%) and MI attendances higher in London (0.6%) than in Paris (0.2%). This may be due to differences in diagnosis coding practices or even clinical procedures used for treating patients (e.g. immediate transfer to cardiac care rather than ED for MI patients). However, the trends observed within weeks were very similar in both systems, implying they are broadly comparable (supplementary figure 2).

A limitation of the statistical methods used here is that the occurrence of previous events (e.g. poor AQ or weather systems) influencing the indicators were not identified or removed from the 2 years of historical data used as RAMMIE training data. This may impact on the RAMMIE model thresholds, though 2 years is considered sufficient for meaningful results (personal communication with R. Morbey).

This study focussed solely on particulate matter, though other pollutants impact on human health. The application of the DAQI levels to both London and Paris mean daily data allowed for an international comparison, based on days with higher than usual $PM_{2.5}$ and/ or PM_{10} specific to each city. The use of the highest daily $PM_{2.5}$/ PM_{10} values was considered, but these were found to be at the high/ very high on the DAQI scale on the majority of days of 2014.

For peer review only

The impact of health warnings and media reporting associated with actual and predicted periods of poor AQ could not be controlled for here. The intention of health warnings, which are reported in the media, is to reduce the impact on human health, encouraging the public to reduce exposure as recommended [9, 43]. There were increases in asthma attendances in children during and following AQ1 in Paris in particular, though these younger age groups appeared unaffected during later events, whereas young adults were more greatly affected by AQ2. These differences of impact by age group in AQ2 may have been due to changes in behaviour of younger age groups so soon after AQ1 and subsequent reduced exposure to poor AQ, rather than a biological response observed in adults only. In addition to the impact of media reporting, France has introduced several other measures when air quality limit values are exceeded in major cities; speed limits, alternate driving days (to limit the number of cars on the road) and free public transportation. The implementation of these measures could have had an impact on the results presented here.

It is important here to underline that variations of near real-time indicators are not easy to attribute directly to poor AQ. An absence of short term variation (e.g. MI in this study) cannot not be interpreted as a total lack of any longer term impact. Similarly, the identification of a significant increase in syndromic indicators reported here (e.g. asthma) has not formally accounted for other associated factors such as climatic conditions (e.g. weather and allergens) or viral circulation. Further time series analysis should be completed to control potential confounding factors.

Future work

This is the first example of the RAMMIE method being applied to a syndromic surveillance system outside the UK. This work has illustrated the potential for RAMMIE to be applied to countries developing new syndromic surveillance systems, or without the infrastructure to support bespoke statistical developments.

For peer review only

This work also promotes further collaboration between different countries to explore methods to harmonize syndromic surveillance systems. Other public health surveillance initiatives have been adopted across Europe to provide a means of reporting singularly comparable variables and statistics across several countries, including: the European monitoring of excess mortality for public health action (EuroMOMO) [44]; the European Influenza Surveillance Scheme (EISS) [45]; establishment of epidemic thresholds for influenza surveillance[46]; the European Antimicrobial Resistance Surveillance Network (EARS-net) [47]; harmonised norovirus surveillance systems also exist [48, 49]. Within this study, although ED indicators were not entirely harmonized, they had been developed to be the most appropriate for each system and country. This work has also stimulated opportunities to explore other areas of public health that could be enhanced using a multinational syndromic surveillance system in particular those due to non-infectious causes such as injury surveillance and these will be addressed in future work.

The apparent difference in the noise to signal ratio between OSCOUR® and EDSSS i.e. background variation was likely due to the size of each respective network. Peaks of abnormal activity were easier to identify in OSCOUR® and therefore future work within PHE is currently focusing on expanding the EDSSS to improve its geographical representativeness and increase the attendance numbers thereby reducing the noise to signal ratio.

This work shows the potential of real-time syndromic surveillance to enhance the public health response to air pollution incidents, even if real-time changes observed through syndromic surveillance data cannot be absolutely related to air pollution. Contemporaneous feedback may be given on the utility of health warnings issued which may aid in the targeting of advice to particular age groups.

For peer review only

The potential for the harmonisation of syndromic surveillance across national borders is also clear, with opportunities to build on local experience to bring international public health benefits.

For peer review only

Acknowledgements

We acknowledge the contribution and support from the ED clinicians and Trust staff; the on-going support of the Royal College of Emergency Medicine; the technical support provided by EMIS Health and L2S2 Ltd in developing the EDSSS system in England and Northern Ireland. We also acknowledge the contribution from the ED structures and clinicians involved in the OSCOUR® network in France; the on-going support of the Federation of the Regional Observatories of Emergencies; the Scientific Society of Emergency Medicine; the air and climate team of Santé publique France and the Regional Unit of Santé publique France in Paris-Ile-de-France region.

We acknowledge and thank Sylvia Medina (Santé Publique France) for their professional input and critical revision of this manuscript.

HEH receives support from the National Institute for Health Research Health Protection Research Unit (NIHR HPRU) in Gastrointestinal Infections. AJE and GES receive support from the National Institute for Health Research Health Protection Research Unit (NIHR HPRU) in Emergency Preparedness and Response. The views expressed are those of the author(s) and not necessarily those of the NHS, the NIHR, the Department of Health or Public Health England.

Ethics

Ethical approval for this work was not required. The anonymised EDSSS health data used in this study were routinely collected at part of the public health function of PHE. The collection and analysis of data provided by the OSCOUR network in the frame of public health surveillance and epidemiological studies has been authorized by the French National Commission for Data protection and Liberties (CNIL).

Funding

For peer review only

This research received no specific grant from any funding agency, commercial or not-for-profit sectors. This surveillance is undertaken as part of the national surveillance functions of Public Health England and Santé Publique France.

Conflicts of interest

None

Contributor statement

HEH contributed to the study design, prepared the ED data for England and Northern Ireland, completed the statistical analyses, drafted the manuscript and provided critical revision and final approval of the manuscript.

RM contributed to the study design, completed the statistical analyses, drafted the manuscript and provided critical revision and final approval of the manuscript.

AF contributed to the study design, prepared the ED data for France and provided critical revision and final approval of the manuscript.

CCS contributed to the study design, critical revision and final approval of the manuscript.

AD contributed to the study design, prepared the air quality data and provided critical revision and final approval of the manuscript.

TCH contributed to the study design, critical revision and final approval of the manuscript.

GES contributed to the study design, critical revision and final approval of the manuscript.

AJE contributed to the study design, critical revision and final approval of the manuscript.

Data sharing statement

Additional data are not available for sharing.

For peer review only

References

1. World Health Organisation. Air Quality Guidelines Global Update 2005. Particulate matter, ozone, nitrogen dioxide and sulfur dioxide: WHO2006.
2. Public Health England. Public Health Outcomes Framework: 3.01 - Fraction of mortality attributable to particulate air pollution. 2017. http://www.phoutcomes.info/public-health-outcomes-framework#page/6/gid/1000043/pat/15/par/E92000001/ati/6/are/E12000007/iid/30101/age/230/sex/4. Accessed 30/03/2017.
3. Pascal M, de Crouy Chanel P, Wagner V, Corso M, Tillier C, Bentayeb M, et al. The mortality impacts of fine particles in France. Sci Total Environ. 2016;571:416-25.
4. DIRECTIVE 2008/50/EC OF THE EUROPEAN PARLIAMENT AND OF THE COUNCIL of 21 May 2008 on ambient air quality and cleaner air for Europe, 2008/50/EC (2008).
5. Air quality guidelines. Global update 2005. Particulate matter, ozone, nitrogen dioxide and sulfur dioxide, (2006).
6. World Air Quality Index project. Air Pollution in the World: Real time Air Quality map. 2016. http://waqi.info/. Accessed 01/03/2016.
7. CITEAIR. Air Quality in Europe. Compare the current air quality in different European Cities. 2007. http://www.airqualitynow.eu/. Accessed 01/013/2016.
8. Department for Environment Food & Rural Affairs. UK-AIR: Information Resource. 2016. http://uk-air.defra.gov.uk/. Accessed 18 March 2016.
9. Department for Environment Food & Rural Affairs. Daily Air Quality Index. 2013. http://uk-air.defra.gov.uk/air-pollution/daqi. Accessed 18 March 2016.
10. Triple S Project. Assessment of syndromic surveillance in Europe. Lancet. 2011;378:1833-4.
11. Hughes HE, Morbey R, Hughes TC, Locker TE, Shannon T, Carmichael C, et al. Using an Emergency Department Syndromic Surveillance System to investigate the impact of extreme cold weather events. Public Health. 2014;128:628-35.
12. Josseran L, Caillere N, Brun-Ney D, Rottner J, Filleul L, Brucker G, et al. Syndromic surveillance and heat wave morbidity: a pilot study based on emergency departments in France. BMC Med Inform Decis Mak. 2009;9:doi: 10.1186/472-6947-9-14.
13. Elliot AJ, Hughes HE, Hughes TC, Locker TE, Brown R, Sarran C, et al. The impact of thunderstorm asthma on emergency department attendances across London during July 2013. Emerg Med J. 2013.
14. Smith GE, Bawa Z, Macklin Y, Morbey R, Dobney A, Vardoulakis S, et al. Using real-time syndromic surveillance systems to help explore the acute impact of the air pollution incident of March/April 2014 in England. Environ Res. 2015;136:500-4.
15. Johnson J, Ginsberg M. Monitoring health effects of wildfires using the biosense system. MMWR Wkly. 2008;57.
16. Rappold AG, Stone SL, Cascio WE, Neas LM, Kilaru VJ, Carraway MS, et al. Peat bog wildfire smoke exposure in rural North Carolina is associated with cardiopulmonary emergency department visits assessed through syndromic surveillance. Environ Health Perspect. 2011;119:1415-20.
17. Le Monde. Pollution : plus de 30 départements en alerte, transports gratuits en Ile-de-France. 2014. http://www.lemonde.fr/planete/article/2014/03/14/pollution-plus-de-30-departements-en-alerte-transports-gratuits-en-ile-de-france_4382974_3244.html. Accessed 26/5/16.
18. RT news. London to be ‘smogged’ by polluted Paris. 2014. https://www.rt.com/uk/154368-london-smog-pollution-paris/. Accessed 26/5/16.
19. BBC. UK air pollution: How bad is it? 2014. http://www.bbc.co.uk/news/uk-26851399. Accessed 26/5/16.
20. Airparif. Airparif: Air quality monitoring network. 2016. http://www.airparif.asso.fr/en/. Accessed 18 March 2016.

For peer review only

21. Affairs DfEFR. Data Selector. https://uk-air.defra.gov.uk/data/data_selector. Accessed 19/07/2016.
22. Airparif. Téléchargements: Results pollutant. http://www.airparif.asso.fr/telechargement/telechargement-polluant. Accessed 06/07/2016.
23. Elliot AJ, Hughes HE, Hughes TC, Locker TE, Shannon T, Heyworth J, et al. Establishing an emergency department syndromic surveillance system to support the London 2012 Olympic and Paralympic Games. Emerg Med J. 2012;29:954-60.
24. World Health Organisation. International Classification of Disease (ICD). 2010. http://www.who.int/classifications/icd/en/. Accessed 1 November 2013.
25. International Health Terminology Standards Development Organisation. SNOMED CT. 2012. http://www.ihtsdo.org/snomed-ct/. Accessed 30 January 2013.
26. Caserio-Schonemann C, Bousquet V, Fouillet A, Henry V. Le système de surveillance syndromique SurSaUD®. Bulletin Epidémiologique Hebdomadaire. 2014:38-44.
27. Morbey RA, Elliot AJ, Charlett A, Verlander NQ, Andrews N, Smith GE. The application of a novel 'rising activity, multi-level mixed effects, indicator emphasis' (RAMMIE) method for syndromic surveillance in England. Bioinformatics. 2015;31:3660-5.
28. Stata version 13.1. College Station, TX 77845 USA: Stata Corporation; 2013.
29. Alhanti BA, Chang HH, Winquist A, Mulholland JA, Darrow LA, Sarnat SE. Ambient air pollution and emergency department visits for asthma: a multi-city assessment of effect modification by age. J Expos Sci Environ Epidemiol. 2016;26:180-8.
30. Elliot AJ, Smith S, Dobney A, Thornes J, Smith GE, Vardoulakis S. Monitoring the effect of air pollution episodes on health care consultations and ambulance call-outs in England during March/April 2014: A retrospective observational analysis. Environmental Pollution. 2016;214:903-11.
31. EUMETSAT. Severe thunderstorms over parts of France. 2014. http://www.eumetsat.int/website/home/Images/ImageLibrary/DAT_2238698.html. Accessed 25/5/16.
32. RFI. Storms to return to France after night of hail, rain, thunder, lightning. 2014. http://en.rfi.fr/visiting-france/20140609-storms-return-france-after-night-hail-rain-lightning-damage. Accessed 25/5/16.
33. MetOffice. UK Climate, Climate Summaries, July 2014. 2015. http://www.metoffice.gov.uk/climate/uk/summaries/2014/july. Accessed 27/5/16 2016.
34. Dabrera G, Murray V, Emberlin J, Ayres JG, Collier C, Clewlow Y, et al. Thunderstorm asthma: an overview of the evidence base and implications for public health advice. QJM. 2013;106:207-17.
35. Wallis DN, Webb J, Brooke D, Brookes B, Brown R, Findlay A, et al. A major outbreak of asthma associated with a thunderstorm: experience of accident and emergency departments and patients' characteristics. BMJ. 1996;312:601-4.
36. Hajat S, Goubet SA, Haines A. Thunderstorm-associated asthma: the effect on GP consultations. Br J Gen Pract. 1997;47:639-41.
37. BBC. 'Thunderstorm asthma' deaths in Melbourne rise to eight. 2016. http://www.bbc.co.uk/news/world-australia-38121579.
38. Julious SA, Osman LM, Jiwa M. Increases in asthma hospital admissions associated with the end of the summer vacation for school-age children with asthma in two cities from England and Scotland. Public Health. 2007;121:482-4.
39. Fleming DM, Cross KW, Sunderland R, Ross AM. Comparison of the seasonal patterns of asthma identified in general practitioner episodes, hospital admissions, and deaths. Thorax. 2000;55:662-5.
40. Lincoln D, Morgan G, Sheppeard V, Jalaludin B, Corbett S, Beard J. Childhood asthma and return to school in Sydney, Australia. Public Health. 2006;120:854-62.
41. Institut de Veille Sanitaire. Surveillance syndromique SurSaUD®: Bulletin du réseau OSCOUR®: Institut de Veille Sanitaire2014 1 October 2014 Contract No.: 496.

For peer review only

42. Health Protection Agency. Emergency Department Syndromic Surveillance System Bulletin: Health Protection Agency, Team R-tSS;2014 23 March 2014.
43. McLaren J, Williams ID. The impact of communicating information about air pollution events on public health. Sci Total Environ. 2015;538:478-91.
44. EuroMOMO. European monitoring of excess mortality for public health action. 2017. http://www.euromomo.eu/index.html. Accessed 30/03/2017.
45. Paget J, Marquet R, Meijer A, van der Velden K. Influenza activity in Europe during eight seasons (1999–2007): an evaluation of the indicators used to measure activity and an assessment of the timing, length and course of peak activity (spread) across Europe. BMC Infect Dis. 2007;7:1-7.
46. Vega T, Lozano JE, Meerhoff T, Snacken R, Mott J, Ortiz de Lejarazu R, et al. Influenza surveillance in Europe: establishing epidemic thresholds by the Moving Epidemic Method. Influenza Other Respir Viruses. 2013;7:546-58.
47. European Centre for Disease Prevention and Control. European Antimicrobial Resistance Surveillance Network (EARS-Net) - See more at: http://ecdc.europa.eu/en/activities/surveillance/EARS-Net/Pages/index.aspx#sthash.5U9aDN1w.dpuf. http://ecdc.europa.eu/en/activities/surveillance/EARS-Net/Pages/index.aspx. Accessed 8/6/14.
48. Vega E, Barclay L, Gregoricus N, Williams K, Lee D, Vinjé J. Novel Surveillance Network for Norovirus Gastroenteritis Outbreaks, United States. Emerg Infect Dis. 2011;17:1389-95.
49. National Institute for Public Health and the Environment R. NoroNet. http://www.rivm.nl/en/Topics/N/NoroNet. Accessed 8/6/16.

For peer review only

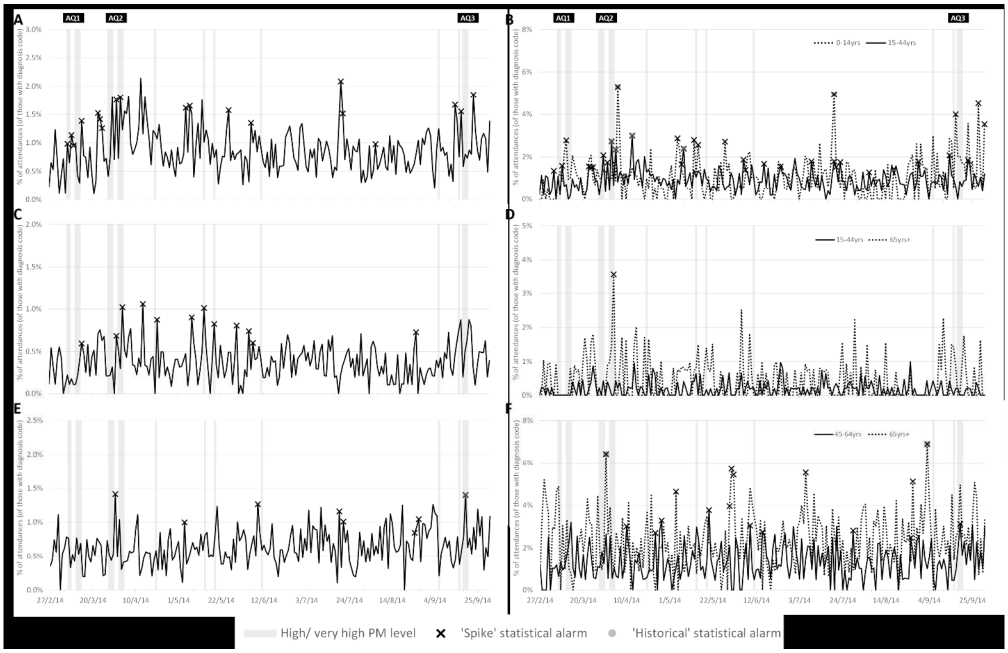

Figure 1: Daily percentages of London ED attendances for syndromic surveillance indicators of A-B; asthma, C-D; difficulty breathing and E-F; myocardial ischaemia, selected age groups, with statistical alarms, reported to EDSSS.

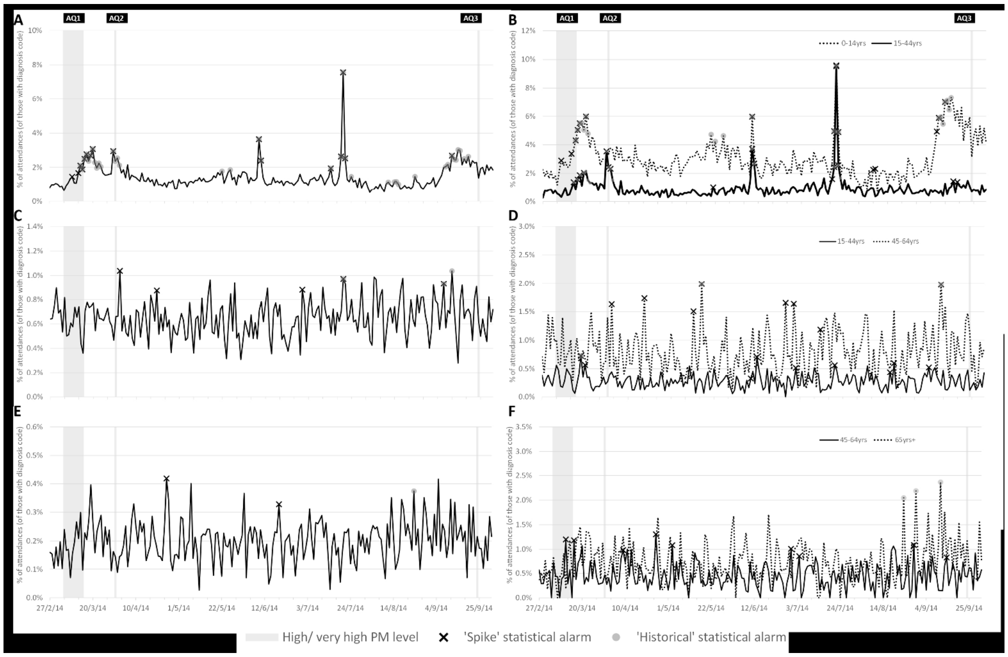

Figure 2: Daily percentages of Paris ED attendances for syndromic surveillance indicators of A-B; asthma, C-D; difficulty breathing and E-F; myocardial ischaemia, selected age groups, with statistical alarms, reported to OSCOUR®.

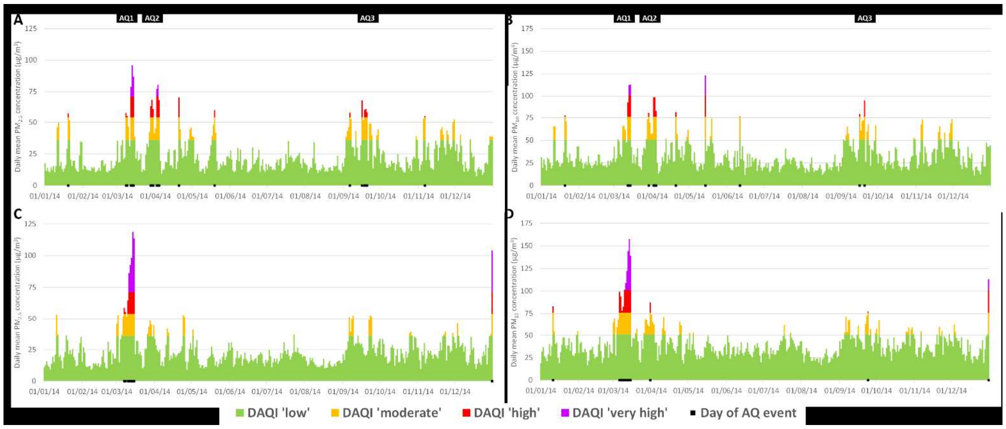

Supplementary Figure 1: Calculated mean daily PM value and corresponding Daily Air Quality Index band, by day during 2014 in London a. PM2.5, b. PM10: Paris c. PM2.5, d. PM10.

For peer review only

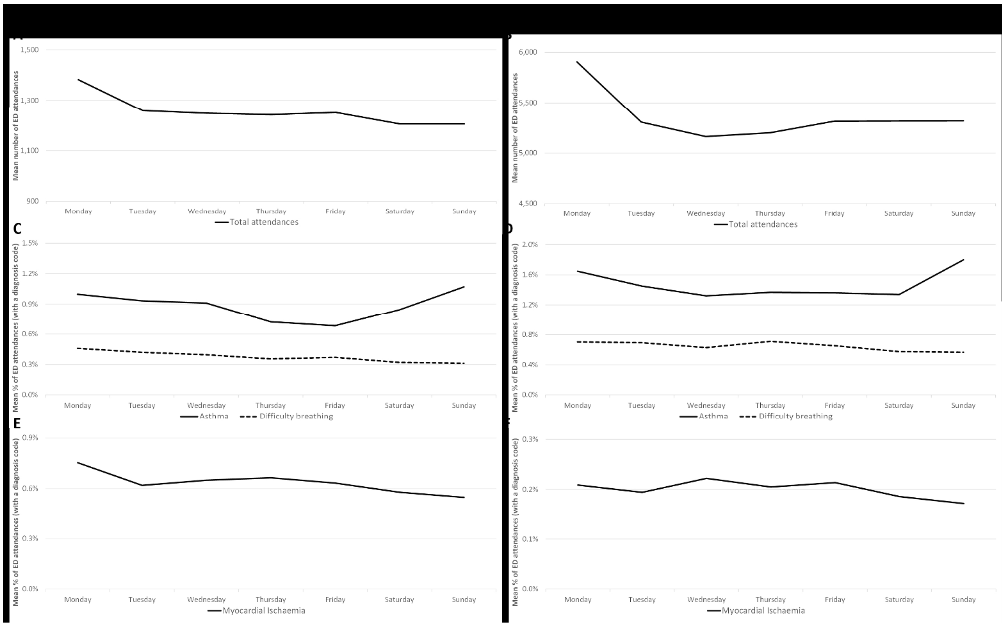

Supplementary Figure 2: Mean emergency department attendances by day of week, 27 February 2014 – 1 October 2014, by syndromic indicators, London reported to EDSSS (a,c,e) and Paris reported to OSCOUR® (b,d,f).

review only

Supplementary table 1: Diagnostic codes mapped to for syndromic surveillance indicators included in the EDSSS (London) and OSCOUR® (Paris) emergency department systems and used in the study.

EDSSS			OSCOUR	
**Indicator**	**Code system**	**Codes**	**Indicator**	**Codes (ICD-10)**
**Asthma**	ICD-10	J450, J459	**Asthme (Asthma)**	J45, J450, J451, J458, J459, J46
	Snomed	30352005, 31387002, 55570000, 57546000, 161527007, 182728008, 195967001, 266364000, 281239006, 304527002, 312453004, 370204008, 370218001, 370219009, 389145006, 401135008, 409663006, 425969006, 445427006, 201031000000108, 340901000000107, 589241000000104, 653751000000109		
**Difficulty breathing/ wheeze**	ICD-10	R06.0, R060, R062, R068	**Dyspnée/ Insuffisance respiratoire (Dyspnoea/ respiratory failure)**	J960, J961, J961+0, J961+1, J969, R060
	Snomed	9763007, 18197001, 23141003, 24612001, 55442000, 56018004, 58596002, 60845006, 62744007, 68095009, 70407001, 161941007, 161947006, 162891007, 162894004, 230145002, 233683003, 267036007, 301703002, 301826004, 307487006, 386813002,		

For peer review only

		427354000, 427679007, 442025000, 276191000000107, 498001000000107, 498011000000109, 502631000000100, 572661000000100, 755581000000101, 755591000000104, 755611000000107, 756081000000102		
**Myocardial ischemia**	ICD-10	I200, I209, I219, I2510	**Ischémie myocardique (Myocardial ischemia)**	I20, I200, I200+0, I201, I208, I209, I21, I210, I2100, I21000, I2108, I211, I2110, I21100, I2118, I2, I212, I2120, I21200, I2128, I213, I2130, I21300, I2138, I214, I2140, I21400, I2148, I219, I2190, I21900, I2198, I22, I220, I2200, I22000, I2208, I221, I2210, I22100, I2218, I228, I2280, I22800, I2288, I229, I2290, I22900, I2298, I23, I230, I231, I232, I233, I234, I235, I236, I238, I24, I240, I241, I248, I249, I25, I250, I251, I252, I253, I254, I255, I256, I258, I259
	Snomed	22298006, 48447003, 53741008, 54329005, 57054005, 59021001, 67682002, 73795002, 155308009, 194828000, 233819005, 233822007, 233843008, 394659003, 398274000, 401303003, 401314000, 414545008, 414795007, 671571000000105		

For peer review only

Supplementary table 2: Results of the Wilcoxon-Mann-Witney test illustrating the standardised value (z value) and significance (P value) of syndromic indicators to days of poor air quality (including 1-3 day lag).

Indicator	City	lag (days)	all ages		0-14yrs		15-44yrs		46-64yrs		65yrs+	
			z value	p value	z value	p value	z value	p value	z value	p value	z value	p value
Asthma	London	0	-0.227	0.8204	-2.857	**0.0043**	1.287	0.1982	1.077	0.2813	-1.009	0.3128
		1	-1.443	0.1490	-3.213	**0.0013**	0.556	0.5784	-0.791	0.4291	-1.026	0.3048
		2	-1.713	**0.0867**	-3.838	**0.0001**	0.787	0.4310	-0.558	0.5768	-1.438	0.1503
		3	-1.627	0.1038	-2.574	**0.0100**	-0.141	0.8876	-0.442	0.6586	0.816	0.4145
	Paris	0	-0.963	0.3356	-1.566	0.1173	0.529	0.5971	-0.624	0.5326	0.000	1.0000
		1	-2.035	**0.0419**	-2.576	**0.0100**	-0.330	0.7418	-1.582	0.1137	-0.354	0.7237
		2	-2.706	**0.0068**	-3.090	**0.0020**	-0.943	0.3454	-2.558	**0.0105**	-0.194	0.8464
		3	-3.049	**0.0023**	-3.201	**0.0014**	-1.797	**0.0724**	-2.77	**0.0056**	-0.756	0.4499
Difficulty Breathing	London	0	-0.055	0.9563	-0.963	0.3357	1.311	0.1898	-0.361	0.7181	-0.140	0.8889
		1	-1.261	0.2073	-2.975	**0.0029**	1.797	**0.0723**	0.445	0.6564	-0.728	0.4666
		2	-0.444	0.6573	-1.385	0.1659	0.223	0.8236	1.452	0.1464	-0.580	0.5620
		3	-1.552	0.1207	-1.236	0.2166	-0.695	0.4872	-0.01	0.9916	-0.296	0.7670
	Paris	0	-0.604	0.5459	0.031	0.9749	-0.585	0.5582	-0.736	0.4615	-0.147	0.8830
		1	-0.057	0.9547	-1.032	0.3021	-0.490	0.6242	0.603	0.5466	-0.078	0.9376
		2	-1.364	0.1725	-1.095	0.2735	-0.674	0.5004	-0.565	0.5722	-1.521	0.1283
		3	-1.144	0.2526	-0.528	0.5974	-0.942	0.3464	0.427	0.6697	-1.222	0.2217
MI	London	0	-0.605	0.5452	-	-	-0.084	0.9327	-1.275	0.2022	0.027	0.9787
		1	-0.588	0.5565	-	-	0.329	0.7421	-1.994	**0.0461**	0.374	0.7084
		2	-0.081	0.9354	-	-	-0.084	0.9327	-0.61	0.5419	0.053	0.9574
		3	-0.571	0.5680	-	-	0.544	0.5862	-1.415	0.1571	-0.695	0.4873
	Paris	0	-0.364	0.7158	0.546	0.5850	-1.257	0.2089	-0.089	0.9293	0.367	0.7138
		1	0.243	0.8082	0.546	0.5850	-1.257	0.2089	-0.022	0.9828	1.594	0.1110
		2	-0.331	0.7408	0.546	0.5850	-0.522	0.6016	-0.235	0.8141	0.635	0.5253
		3	-0.676	0.4992	0.546	0.5850	-0.578	0.5630	0.384	0.7011	-0.403	0.6872

Figures in **bold** are significant to the 90% significance level; those **bold and underlined** to the 95% significance level.

For peer review only

BMJ Open

A retrospective observational study of emergency department syndromic surveillance data during air pollution episodes across London and Paris in 2014

Journal:	*BMJ Open*
Manuscript ID	bmjopen-2017-018732.R1
Article Type:	Research
Date Submitted by the Author:	30-Nov-2017

Complete List of Authors:	Hughes, Helen; Public Health England, Real-time Syndromic Surveillance; The Farr Institute @ HeRC,<br>Morbey, Roger; Public Health England, Real-time Syndromic Surveillance Team<br>Fouillet, Anne; Sante publique France, Syndromic Surveillance Unit<br>Caserio-Schönemann, Céline; Sante publique France, Syndromic Surveillance Unit<br>Dobney, Alec; Public Health England, Environmental Hazards and Emergencies Department<br>Hughes, Thomas; John Radcliffe Hospital, Emergency Department; Royal College of Emergency Medicine<br>Smith, Gillian; Public Health England, Real-time Syndromic Surveillance Team<br>Elliot, Alex; Health Protection Agency, Real-time Syndromic Surveillance Team

**Primary Subject Heading**:	Public health
Secondary Subject Heading:	Emergency medicine, Global health, Epidemiology
Keywords:	syndromic surveillance, air quality, particulate matter, emergency department, Asthma < THORACIC MEDICINE

SCHOLARONE™ Manuscripts

A retrospective observational study of emergency department syndromic surveillance data during air pollution episodes across London and Paris in 2014

H. E. Hughes[1,2], R. Morbey[1], A. Fouillet[3], C. Caserio-Schönemann[3], A Dobney[4], T. C. Hughes[5,6], G. E. Smith[1], and A. J. Elliot[1]

1 Real-time Syndromic Surveillance Team, National Infection Service, Public Health England, Birmingham, UK;

2 Farr Institute at HeRC, Liverpool, UK

3 Syndromic Surveillance Unit, Santé publique France, the national public health agency, Saint-Maurice, France;

4 Environmental Hazards and Emergencies Department, Centre for Radiation, Chemical and Environmental Hazards, Public Health England, Birmingham, UK;

5 John Radcliffe Hospital, Oxford, UK;

6 The Royal College of Emergency Medicine, London, UK.

Address for correspondence:

Helen E Hughes, Real-time Syndromic Surveillance Team, Public Health England, 1st Floor, 5 St Philip's Place, Birmingham B3 2PW, UK

email: h.hughes7@liverpool.ac.uk

phone: +44 (0)844 225 3560 option 4 > option 2; fax: +44 (0)121 236 2215

Reprints will not be available from the author.

Word count: 3613

Key words: Syndromic surveillance, Emergency Department, Air quality, particulate matter, asthma, London, Paris

For peer review only

Abstract

Introduction

Poor air quality (AQ) is a global public health issue and AQ events can span across countries. Using emergency department (ED) syndromic surveillance from England and France, we describe changes in human health indicators during periods of particularly poor AQ in London and Paris during 2014.

Methods

Using daily AQ data for 2014, we identified 3 periods of poor AQ affecting both London and Paris. Anonymised near real-time ED attendance syndromic surveillance data from EDs across England and France were used to monitor the health impact of poor AQ.

Using the routine English syndromic surveillance detection methods, increases in selected ED syndromic indicators (asthma, difficulty breathing and myocardial ischaemia), in total and by age, were identified and compared to periods of poor AQ in each city. Retrospective Wilcoxon-Mann-Whitney tests were used to identify significant increases in ED attendance data on days with (and up to 3 days following) poor AQ.

Results

Almost 1.5 million ED attendances were recorded during the study period (27/2/14-1/10/14). Significant increases in ED attendances for asthma were identified around periods of poor AQ in both cities, especially in children (0-14yrs). Some variation was seen in Paris with a rapid increase during the first AQ period in asthma attendances amongst children (0-14yrs), whereas during the second period the increase was greater in adults.

Discussion

For peer review only

This work demonstrates the public health value of real-time syndromic surveillance in response to air pollution incidents, and the potential for further cross-border harmonisation to provide Europe-wide early alerting to health impacts.

Strengths and limitations of this study

- Routinely collected syndromic surveillance data from both England (London) and France (Paris) were analysed using similar health indicators
- A single statistical method, designed specifically for daily syndromic surveillance, was applied to data from both cities
- Air quality measurements were standardised across both cities, to overcome differences in the standard reporting from each
- Pollutants other than particulate matter were not included, though they may be responsible for impacts on human health
- We could not control for the potential effects of health warnings and media coverage on health care seeking behaviour

For peer review only

Introduction

Air quality

Air pollution has negative impacts on human health. Short term exposure to poor air quality can affect lung function, including exacerbating asthma symptoms, and is associated with other acute deteriorations in respiratory and cardiovascular health [1]. Similar health effects have also been reported due to long term exposure, with exposure to ambient air pollution associated with lung cancer and chronic respiratory and cardiovascular conditions [1]. In addition to illness within the community and increased need for health care, air pollution is also associated with increased mortality, with an estimated 4.7% of deaths in the England attributed to air pollution [2] and 9% of deaths in France attributed to $PM_{2.5}$ [3].

Air quality (AQ) monitoring identifies long term trends informing policy, provides evidence of meeting (or missing) statutory target levels and quantifies the impact of preventative measures [4, 5]. Daily AQ monitoring enables daily reporting of both actual and modelled AQ (predicting one or more days in advance), for whole countries and/or individual cities, as well as on a smaller scale around individual monitoring stations [6-8]. This information is increasingly easy to access through websites and apps and is often reported through the media, especially following formal health warnings [9].

Syndromic surveillance

Syndromic surveillance initially focussed on infectious diseases such as influenza but is increasingly being used for other non-infectious public health events. This type of surveillance uses real-time data from patient contacts with health care services (e.g. telephone helplines, general practice/ family doctors, or emergency departments). Patient contacts/ attendances are grouped by diagnoses/ symptoms creating syndromic indicators such as 'respiratory' or 'gastrointestinal', providing valuable information for public health action [10]. The use of emergency department (ED) data lends itself particularly well to the

For peer review only

syndromic surveillance of non-infectious public health events, with patients seeking attention for a range of acute conditions [11-13]. Previous investigation of periods of poor air quality have shown associated increases in health seeking behaviour as evidenced by syndromic surveillance, particularly for asthma and/ or difficulty breathing and heart failure [14-16], though not for myocardial infarction [16].

Aims

During March and early April 2014 there was a period of widespread poor AQ across Europe. In particular, the urban conurbations of London (England) and Paris (France) were affected by high temperatures, Saharan dust and industrial emissions, resulting in widespread media attention [17-19]. Here, we use routine emergency department (ED) syndromic surveillance data collected across London and Paris during poor AQ periods throughout 2014 to investigate the compatibility of the two countries' ED syndromic surveillance systems and describe the public health impact and associated short-term changes in health care seeking behaviour for selected respiratory and cardiac syndromes across different age groups.

For peer review only

Methods

Air quality data

The area studied here has been limited to London and the whole Paris region (Île-de-France), rather than a country level. In England, the Department for Environment, Food and Rural Affairs monitors and reports on levels of air pollution using monitoring stations and provides health advice using the Daily Air Quality Index (DAQI) [9]. Air quality in the Paris region is monitored by Airparif and reported using the Citeair index [20].

Both DAQI and Citeair systems monitor and report on multiple pollutants, however each index is reported using different methodology. Therefore the daily pollution levels across both London and Paris were standardised here, using the reported levels of particulate matter ($PM_{2.5}$ and PM_{10}). The city wide average value for each PM on each calendar day was calculated as a mean of the maximum values reported for each monitoring station on that day, in that city [21, 22]. Periods of poor AQ were then defined as those when either the calculated $PM_{2.5}$ or PM_{10} average value corresponded to the DAQI index levels of 7-10, which are classified as 'high', to 'very high' ($PM_{2.5}$ >=54 µg/m³ or PM_{10} >=76 µg/m³). At these levels people, including those with no pre-existing medical conditions, are advised to consider reducing their activity levels, particularly outdoors [8].

Emergency department syndromic surveillance data

The Emergency Department Syndromic Surveillance System (EDSSS), is a sentinel ED system coordinated by Public Health England (PHE), collecting anonymised data from participating EDs on a daily basis (data for the previous day 00:00 to 23:59 are transferred to PHE the following morning) [23]. Diagnosis coding in EDs in England was not standardised at the time of this investigation. Each ED had a list of diagnosis terms created locally which was available for selection in the patient attendance record. These diagnostic terms have associated codes linked to them with each ED using one of three codesets: Commissioning

For peer review only - http://bmjopen.bmj.com/site/about/guidelines.xhtml

Data Set Accident and Emergency Diagnosis Tables [24], ICD-10 [25] or Snomed CT [26]. EDs eligible for inclusion in this study were defined as those reporting using ICD-10 or Snomed CT diagnosis coding systems which provide the level of detail required for the identification of the indicators of interest. This investigation included EDSSS participating EDs within the London PHE Centre, which all fall within central London.

The French national ED syndromic surveillance system collects daily data from the Organisation de la Surveillance COordonnée des URgences (OSCOUR®) network of EDs, coordinated by Santé Publique France [27] (again, data for the previous day 00:00 to 23:59 are transferred and analysed the following morning for 85% of ED attendances. OSCOUR® allowing for updates and delayed reporting, the missing 15% of ED attendances are reported in the following 2 days [28]). All EDs reporting to OSCOUR® use ICD-10 for the coding of diagnoses selected in the patient attendance record [28]. Aggregated, anonymised daily data for the Paris region were made available for this analysis.

Epidemiological analysis

Syndromic indicators (asthma, difficulty breathing and myocardial ischaemia (MI) (table 1)) were selected from the comparable indicators already created for each system, based on clinical knowledge and experience of the potential health effects linked to air pollution and those used in previous syndromic surveillance work.

Table 1: Syndromic surveillance indicators included in the EDSSS (London) and OSCOUR® (Paris) emergency department systems and used in the study

EDSSS (London)	OSCOUR® (Paris)	Reported here as
Asthma	Asthme	Asthma
Wheeze/ difficulty breathing	Dyspnée/ Insuffisance respiratoire	Difficulty breathing
Myocardial ischaemia	Ischémie myocardique	Myocardial Ischaemia (MI)

For peer review only

These syndromic surveillance indicators, which are routinely used in both EDSSS and OSCOUR® are an aggregation of relevant diagnostic codes representing similar diagnostic terms and available in the patient record. Though 'diagnostic' information these diagnoses have potentially been made before any final confirmation and may be based on the symptoms presented, with no level of certainty indicated. The overall asthma and MI indicator groupings were very similar in each system, with the terms included all describing either asthma or myocardial ischaemic conditions. Differences were found in non-asthma difficulty breathing type indicators, where EDSSS included symptomatic wheeze/ difficulty breathing type diagnoses and OSCOUR® included dyspnoea/ respiratory failure diagnoses (table 2). Please note: not every code listed was reported by – or even available for selection – from every ED. More relevant codes may exist for each indicator than described here, however only codes reported to EDSSS/ OSCOUR® in this study are included).

Table 2: Diagnostic codes mapped to for syndromic surveillance indicators included in the EDSSS (London) and OSCOUR® (Paris) emergency department systems and used in the study.

**EDSSS**			**OSCOUR**	
**Indicator**	**Code system**	**Codes**	**Indicator**	**Codes (ICD-10)**
**Asthma**	ICD-10	J450, J459	**Asthme (Asthma)**	J45, J450, J451, J458, J459, J46
	Snomed	30352005, 31387002, 55570000, 57546000, 161527007, 182728008, 195967001, 266364000, 281239006, 304527002, 312453004, 370204008, 370218001, 370219009, 389145006, 401135008, 409663006,		

For peer review only

		425969006, 445427006, 20103100000108, 340901000000107, 589241000000104, 653751000000109		
**Difficulty breathing/ wheeze**	ICD-10	R06.0, R060, R062, R068	**Dyspnée/ Insuffisance respiratoire (Dyspnoea/ respiratory failure)**	J960, J961, J961+0, J961+1, J969, R060
	Snomed	9763007, 18197001, 23141003, 24612001, 55442000, 56018004, 58596002, 60845006, 62744007, 68095009, 70407001, 161941007, 161947006, 162891007, 162894004, 230145002, 233683003, 267036007, 301703002, 301826004, 307487006, 386813002, 427354000, 427679007, 442025000, 276191000000107, 498001000000107, 498011000000109, 502631000000100, 572661000000100, 755581000000101, 755591000000104, 755611000000107, 756081000000102		
**Myocardial ischemia**	ICD-10	I200, I209, I219, I2510	**Ischémie myocardique (Myocardial ischemia)**	I20, I200, I200+0, I201, I208, I209, I21, I210, I2100, I21000, I2108, I211, I2110, I21100, I2118, I2, I212, I2120, I21200, I2128, I213, I2130, I21300, I2138, I214, I2140, I21400, I2148, I219, I2190, I21900, I2198, I22, I220, I2200, I22000, I2208,
	Snomed	22298006, 48447003, 53741008, 54329005, 57054005, 59021001, 67682002, 73795002, 155308009, 194828000, 233819005, 233822007, 233843008, 394659003,		

For peer review only

		398274000, 401303003, 401314000, 414545008, 414795007, 671571000000105		I221, I2210, I22100, I2218, I228, I2280, I22800, I2288, I229, I2290, I22900, I2298, I23, I230, I231, I232, I233, I234, I235, I236, I238, I24, I240, I241, I248, I249, I25, I250, I251, I252, I253, I254, I255, I256, I258, I259

For peer review only

For each syndromic surveillance system, attendances were aggregated by age group defined as 0-14, 15-44, 45-64 and 65 years and over.

The epidemiological analysis of ED attendance data included construction of trends in attendances for each syndromic indicator, both for all ages and for each age group, and city. The daily percentage of attendances for each indicator were calculated using the number of [2]attendances within an indicator (numerator) and the daily number of total (all cause) attendances with a diagnosis code within each surveillance system (denominator). .

Statistical analysis

The EDSSS and OSCOUR® are both live public health surveillance systems prospectively collecting data with automated contemporaneous statistical algorithms underpinning the detection of unusual activity. We applied the routine syndromic surveillance statistical detection algorithm from England: the RAMMIE method (Rising Activity, Multi-level Mixed effects Indicator Emphasis [29]). RAMMIE was applied to both English and French ED data, including to age specific data. Using RAMMIE two separate statistical thresholds were calculated: a ‘2-year’ threshold (based on the previous 2 years of data) to identify significant activity compared to previous years, and a ‘2-week’ threshold (based on the previous two weeks) to identify recent, statistically significant, increases in daily activity. RAMMIE routinely allows for the prioritisation of alarms to facilitate the identification of significant

activity, however, this function was not used here to ensure that all statistically significant activity was identified, and not just those signals prioritised by RAMMIE.

To ensure that sufficient data were included here to cover each of the AQ events identified, a study period of a minimum of 7 days pre the first and 7 days post the final period of poor AQ identified in London/ Paris during 2014 was selected. A further period of 2 years of data prior to the first AQ event provided required baseline data for the RAMMIE method.

In addition to the RAMMIE analysis, the Wilcoxon-Mann-Whitney non-parametric test was used to test for significant differences in the syndromic indicators during the 2014 study period, by age group between those days with a poor AQ and those without. To allow for the possibility of a delayed response, separate analyses were conducted incorporating lags of one to three days following a day of poor AQ.

All analyses were undertaken using Stata v13.1 [30].

For peer review only

Results

Air quality events

During 2014, several periods of poor AQ were identified where the ‘high’ or ‘very high’ air pollution thresholds for particulate matter ($PM_{2.5}$ and/or PM_{10}) had been breached in both London and Paris (figure 1). Periods of poor air quality in Paris were generally observed to be of a longer duration and with higher DAQI levels than in London, though more individual days of poor AQ were identified in London. Two main periods of poor AQ overlapped in these cities in mid-March (AQ1, the largest event in both locations and where transboundary dust from the Sahara contributed to the makeup of the particulate matter fraction [14]) and early April (AQ2, mainly in London, though a 1 day PM_{10} spike in Paris), with a third, less severe period during September occurring in both cities within a 7 day period (AQ3) (table 3).

An overall study period was defined as 27 February 2014 – 1 October 2014 (216 days), to encompass each period where poor AQ occurred in both London and Paris, including 7 days before and after the first and final AQ events identified (table 3).

Table 3: Dates of poor air quality, coinciding in London and Paris during 2014

	AQ1	AQ2	AQ3	Total AQ days
**London**	08/03/14 - 14/03/14	28/03/14 - 04/04/14	16/09/14 - 20/09/14	15
**Paris**	06/03/14 - 15/03/14	31/03/14	24/09/14	12

For peer review only

ED attendances

Over the study period 1,436,163 ED attendances were recorded across both London and Paris (table 4). Total attendances were higher in Paris (1,163,353; from 58 EDs) than London (272,810; from 5 EDs, 3 using ICD-10, 2 using Snomed CT). A comparable level of diagnosis coding was included in each city with 79% of London attendances and 72% of Paris attendances including a clinical diagnosis code.

On a weekly basis, total ED attendances in both London and Paris showed similar trends, with a peak observed on a Monday. Examination of indicator trends illustrated that there were further similarities between EDSSS and OSCOUR® with highest levels of asthma attendances (as a percentage of attendances with a diagnosis code; and lowest levels of MI attendances, reported on Sundays (figure 2).

For peer review only

Table 4: Attendances recorded in EDs, by city, over the study period (27/02/14-01/10/14)

		ED Attendances			*Attendances with a diagnosis*			*Indicator attendances*		
**City**	**EDs**	**ICD10**	**Snomed**	**Total**	**ICD10**	**Snomed**	**Total**	**Asthma**	**Difficulty breathing**	**Myocardial ischaemia**
London	5*	115,539	157,271	272,810	81,980 (71%)	132,750 (84%)	214,730 (79%)	1,893 (0.9%)	812 (0.4%)	1,370 (0.6%)
Paris	58	1,163,353	-	1,163,353	840,309 (72%)	-	840,309 (72%)	12,374 (1.5%)	5,433 (0.6%)	1,685 (0.2%)

*1 small ED (which used ICD-10) stopped reporting to EDSSS on 10/09/2014. All 5 EDs were included in descriptive and RAMMIE analysis; 4 EDs that reported throughout were included in Wilcoxon-Mann-Whitney testing.

For peer review only

ED attendances during poor air quality periods

The application of RAMMIE generated statistical 'alarms', where the number of attendances observed was greater than threshold calculated from the baseline data ('2-year' alarm; based on previous 2 years of data) and/or more recent trend data ('2-week' alarm; based on previous 2 weeks). RAMMIE alarms, where significantly higher than expected levels of ED attendances were observed, showed a degree of correspondence with the dates of poor AQ (figures 3 & 4).

London ED attendances

Small increases in asthma attendances (all ages) in London EDs were observed following AQ1 (figure 3a). ED asthma attendances continued to increase during and immediately following AQ2. RAMMIE 2-week alarms were reported for the increases in asthma (all ages) immediately following AQ1 in London, indicating an attendance level higher than the previous 2 weeks. However, single 2-week alarms were not unusual in these data and were also observed during periods with no reported AQ issues. 2-year asthma alarms are less frequent and were not observed in these data during the study period.

The observed increase of asthma attendances during the AQ2 episode in London was most evident in children aged 0-14 years, and young adults (15-44 years) with each age group reaching a peak in attendances 1 to 2 days later (figure 3b). Asthma attendances for older adults showed no evidence of increase around periods of poor AQ (data not shown).

An additional peak in asthma (all ages) attendances was observed on 20/7/14 (figure 3a), particularly in children (0-14yrs; figure 3b), though there was no poor AQ identified at that time. During early September increases in all age attendances for asthma, largely driven by child attendances (0-14yrs), were observed to have started prior to AQ3.

For peer review only

A small increase in difficulty breathing attendances (all ages) immediately following AQ2 (figure 3c), was most apparent in the older adults (65 and over years; figure 3d). This single day peak was the highest level seen in this age group, around double the usual level, though not significantly higher than historical data. Other age groups were not affected.

MI attendances were less common than asthma attendances in London EDs (table 4) and affected the adult age groups almost exclusively, as would be expected. Though a peak (resulting in both 2-week and 2-year alarm) in MI attendances was observed during AQ2, particularly in those aged 65yrs and over, a similar peak also occurred in late September, several days prior to the AQ3 and 2-week alarms occurred quite frequently throughout the year (figure 3e & f).

Paris ED attendances

Clear increases in ED attendances (all ages) for asthma occurred during both AQ1 and AQ2 in Paris (figure 4a). These increases were detected by RAMMIE as statistically significant in comparison to previous years (2-year alarm), as well as compared to the preceding 2 weeks (2-week alarm). However, when broken down by age, the increase in asthma attendances in the 0-14 years age group occurred during AQ1, but not AQ2; while asthma attendances in young adults (15-44yrs) were greater during AQ2 than AQ1. No statistical alarms were observed for asthma in children around AQ2, though they were present for young adults (figure 4b).

The largest peak in asthma attendances was observed on 20/07/14, for all ages apart from 65yrs and over (data not shown), matching the spike seen in London, despite this not being a poor AQ period. One further peak in asthma attendances, apparent in all ages and individual age groups, was observed on 9-10/6/14 (figure 4a & b). The observed peaks were not concomitant with any period of poor AQ in Paris, nor London.

For peer review only

Similar to London, an increase in asthma attendances was observed in Paris at the beginning of September, prior to AQ3, driven predominantly by children (0-14 years).

Difficulty breathing attendances in Paris were much lower than for asthma overall, with a single increase after AQ2 (figure 4c). Within the 15-44yrs age group there was, however an increase in difficulty breathing attendances following AQ1 (figure 4d).

Attendances for MI in Paris showed no evidence of increase in Paris during/ following days of poor AQ (figures 4e & f), though some statistical alarms were observed throughout the year, particularly a series of three 2-year alarms during late August and September in those aged 65 years and over.

Retrospective statistical analysis

Wilcoxon-Mann-Whitney test results provide further evidence, alongside the descriptive epidemiology and RAMMIE results, that there is a strong association between days of poor AQ and asthma attendances all ages and particularly in children 0-14 years (table 5). Furthermore, the statistical significances of the associations between asthma attendances and poor AQ were highest when modelled with a lag between the day of poor AQ and attendances; two days for London and three days for Paris. Though there was some evidence of increased attendances for difficulty breathing and MI in some age groups in London one day after poor AQ, these alarms were single significant values (rather than the grouping of significant asthma results by age group; figure 3d&f). These increased MI and difficulty breathing attendances in the day following poor AQ were not seen in the Paris data (figure 4d&f).

For peer review only

Table 5: Results of the Wilcoxon-Mann-Witney test illustrating the standardised value (z value) and significance (P value) of syndromic indicators to days of poor air quality (including 1-3 day lag).

		lag	all ages		0-14yrs		15-44yrs		46-64yrs		65yrs+	
Indicator	City	(days)	z value	p value	z value	p value	z value	p value	z value	p value	z value	p value
Asthma	London	0	-0.227	0.8204	-2.857	**0.0043**	1.287	0.1982	1.077	0.2813	-1.009	0.3128
		1	-1.443	0.1490	-3.213	**0.0013**	0.556	0.5784	-0.791	0.4291	-1.026	0.3048
		2	-1.713	**0.0867**	-3.838	**0.0001**	0.787	0.4310	-0.558	0.5768	-1.438	0.1503
		3	-1.627	0.1038	-2.574	**0.0100**	-0.141	0.8876	-0.442	0.6586	0.816	0.4145
	Paris	0	-0.963	0.3356	-1.566	0.1173	0.529	0.5971	-0.624	0.5326	0.000	1.0000
		1	-2.035	**0.0419**	-2.576	**0.0100**	-0.330	0.7418	-1.582	0.1137	-0.354	0.7237
		2	-2.706	**0.0068**	-3.090	**0.0020**	-0.943	0.3454	-2.558	**0.0105**	-0.194	0.8464
		3	-3.049	**0.0023**	-3.201	**0.0014**	-1.797	**0.0724**	-2.77	**0.0056**	-0.756	0.4499
Difficulty Breathing	London	0	-0.055	0.9563	-0.963	0.3357	1.311	0.1898	-0.361	0.7181	-0.140	0.8889
		1	-1.261	0.2073	-2.975	**0.0029**	1.797	**0.0723**	0.445	0.6564	-0.728	0.4666
		2	-0.444	0.6573	-1.385	0.1659	0.223	0.8236	1.452	0.1464	-0.580	0.5620
		3	-1.552	0.1207	-1.236	0.2166	-0.695	0.4872	-0.01	0.9916	-0.296	0.7670
	Paris	0	-0.604	0.5459	0.031	0.9749	-0.585	0.5582	-0.736	0.4615	-0.147	0.8830
		1	-0.057	0.9547	-1.032	0.3021	-0.490	0.6242	0.603	0.5466	-0.078	0.9376
		2	-1.364	0.1725	-1.095	0.2735	-0.674	0.5004	-0.565	0.5722	-1.521	0.1283
		3	-1.144	0.2526	-0.528	0.5974	-0.942	0.3464	0.427	0.6697	-1.222	0.2217
MI	London	0	-0.605	0.5452	-	-	-0.084	0.9327	-1.275	0.2022	0.027	0.9787
		1	-0.588	0.5565	-	-	0.329	0.7421	-1.994	**0.0461**	0.374	0.7084
		2	-0.081	0.9354	-	-	-0.084	0.9327	-0.61	0.5419	0.053	0.9574
		3	-0.571	0.5680	-	-	0.544	0.5862	-1.415	0.1571	-0.695	0.4873
	Paris	0	-0.364	0.7158	0.546	0.5850	-1.257	0.2089	-0.089	0.9293	0.367	0.7138
		1	0.243	0.8082	0.546	0.5850	-1.257	0.2089	-0.022	0.9828	1.594	0.1110
		2	-0.331	0.7408	0.546	0.5850	-0.522	0.6016	-0.235	0.8141	0.635	0.5253
		3	-0.676	0.4992	0.546	0.5850	-0.578	0.5630	0.384	0.7011	-0.403	0.6872

Figures in **bold** are significant to the 90% significance level; those **bold and underlined** to the 95% significance level.

For peer review only

Discussion

Main findings

We used two national ED syndromic surveillance systems to describe and compare the short-term changes in ED indicators during periods of poor AQ in two European capital cities. The AQ events reported here in Paris and London were related to the same pollutants ($PM_{2.5}$/ PM_{10}), and were very similar in terms of the dates and duration, and changes in public health outcomes in terms of ED attendances.

The most sensitive ED indicator during periods of poor AQ was asthma, with the impact most apparent up to 3 days after a day of poor AQ. The breakdown of attendances by age group revealed some differences, with the strongest associations overall seen between poor AQ and asthma attendances in children. This finding was consistent with previous studies which have shown children to be more susceptible to exacerbation of asthma symptoms requiring health care in association with air pollution [31].

The investigation of individual AQ incidents demonstrated the potential for differing levels of impact on different age groups at different times. Though generally children were most affected by AQ, a large increase in adult asthma attendances was observed during and immediately following AQ2 in both London and Paris. Within England this increase in attendances around AQ2 has previously been described [32]. As the second period of poor AQ to occur in a short period of time, media coverage and the associated communication of health warning information and interventions put in place during AQ2 may have resulted in changes in behaviour which affected the levels of exposure of different age groups.

In addition to the increases observed during AQ periods, a sharp increase in asthma attendances (all ages) was observed in Paris on 9-10/06/14, and in both London and Paris on 20/07/14. These peaks did not coincide with any AQ event identified here, however, additional meteorological data (not presented) revealed periods of major thunderstorm

activity within each city at the time [33-35]. These findings match those previously reported, including from the EDSSS, describing the health effects of 'thunderstorm asthma', where sudden exacerbation of asthma symptoms results in increased health care seeking behaviour over a short time period [13, 36-39], possibly due to increased levels of pollen and fungal spores, though the mechanism has not yet been confirmed [36].

We also observed further increases in asthma attendances in both Paris and London (and England and France as whole; data not shown) towards the start of September. This increase was particularly evident in children and is likely linked to an annual 'back to school' increase in asthma type attendances in EDs during September [40-42].

Other syndromic indicators investigated showed little (difficulty breathing), to no (MI) association with the AQ incidents identified here.

Strengths and limitations

The OSCOUR® system includes greater representative coverage nationally, with more EDs participating than the sentinel EDSSS system (540 EDs across France were reporting to OSCOUR® [43]). While 34 EDs across England and Northern Ireland were reporting to EDSSS at 20 March 2014, the five reported here were located in London making the EDSSS more representative in London than at the national level [44]). The large number of OSCOUR® EDs reported here resulted in much more stable data from Paris, reducing background noise and allowing clearer differentiation of spikes/increases in attendances. The smaller number of attendances within the EDSSS data made identifying spikes 'harder', however the use of RAMMIE enables significant increases in attendances to be identified, even when not initially obvious [29].

Despite underlying differences in the method of data collection, with EDSSS taking a single snapshot of daily attendances and OSCOUR® allowing the initial snapshot data to be updated retrospectively, both systems reported over 70% completion of the clinical diagnosis

For peer review only - http://bmjopen.bmj.com/site/about/guidelines.xhtml

For peer review only

field making diagnostic data comparable. Furthermore, though these systems were developed individually, it was found that the syndromic indicators used within each system were similar, making comparisons of health impact possible. However, the EDSSS used a wheeze/ difficulty breathing indicator whereas OSCOUR® used a difficulty breathing/ respiratory failure indicator. This difference is, in part, likely to be related to the use of different clinical coding systems, with the identification of symptoms (e.g. wheeze) more difficult using ICD-10 (as used in France) than Snomed CT (used by some EDs in England).

The use of percentage or ED visits (with a diagnosis code), as an indication of ED attendances (rather than actual numbers), as reported here may be impacted by the overall levels of ED attendances (and levels of diagnostic coding) on any one day. Though travel and outdoor activities are discouraged during AQ events, there are other factors which have a much greater impact on ED attendances (such as national and school holiday periods). The patterns and total numbers of attendances during 2014, including AQ periods, were not different to those seen in other years. This is different to the changes seen during extreme cold weather when total attendances has been seen in to be reduced in the English EDSSS, as transportation is not physically possible for most people [11]. By using percentage of attendances the impact of events, such as periods of poor AQ, can be clearly seen in terms of changes in ED workload, such as changes in case mix and/ or age groups attending.

The levels of attendances for each indicator were different between cities, with respiratory indicators higher in Paris (asthma 1.5%, difficulty breathing 0.7%), than London (asthma 0.9%, difficulty breathing 0.4%) and MI attendances higher in London (0.6%) than in Paris (0.2%). This disparity in attendance levels between countries may be due to differences in diagnosis coding practices, clinical procedures used for treating patients (e.g. immediate transfer to cardiac care rather than ED for MI patients) or even areas of specialty for each ED (e.g. some London EDs are part of specialist heart care hospitals so may see more MI

For peer review only

patients). However, the trends observed within weeks were very similar in both systems, implying they are broadly comparable (figure 2).

A limitation of the statistical methods used here is that the occurrence of previous events (e.g. poor AQ or weather systems) influencing the indicators were not identified or removed from the 2 years of historical data used as RAMMIE training data. The potential inclusion of unrecognised events may impact on the RAMMIE model thresholds, though 2 years is considered sufficient for meaningful results (personal communication with R. Morbey).

This study focussed solely on particulate matter, though other pollutants impact on human health. The application of the DAQI levels to both London and Paris mean daily data allowed for an international comparison, based on days with higher than usual $PM_{2.5}$ and/ or PM_{10} specific to each city. The use of the highest daily $PM_{2.5}$/ PM_{10} values was considered, but these values were found to be at the high/ very high on the DAQI scale on the majority of days of 2014.

The impact of health warnings and media reporting associated with actual and predicted periods of poor AQ could not be controlled for here. The intention of health warnings, which are reported in the media, is to reduce the impact on human health, encouraging the public to reduce exposure as recommended [9, 45]. There were increases in asthma attendances in children during and following AQ1 in Paris in particular, though these younger age groups appeared unaffected during later events, whereas young adults were more greatly affected by AQ2. These differences of impact by age group in AQ2 may have been due to changes in behaviour of younger age groups so soon after AQ1 and subsequent reduced exposure to poor AQ, rather than a biological response observed in adults only. In addition to the impact of media reporting, France has introduced several other measures when air quality limit values are exceeded in major cities; speed limits, alternate driving days (to limit the number of cars

For peer review only - http://bmjopen.bmj.com/site/about/guidelines.xhtml

on the road) and free public transportation. The implementation of these measures could have had an impact on the results presented here.

It is important here to underline that variations of near real-time indicators are not easy to attribute directly to poor AQ. An absence of short term variation (e.g. MI in this study) cannot not be interpreted as a total lack of any longer term impact. Similarly, the identification of a significant increase in syndromic indicators reported here (e.g. asthma) has not formally accounted for other associated factors such as climatic conditions (e.g. weather and allergens) or viral circulation. Further time series analysis should be completed to control potential confounding factors.

Future work

This work has prompted the systematic investigation of asthma attendances by age group around AQ events in England and Northern Ireland, using the EDSSS. In France (following the March 2014 periods of poor AQ reported here), the health authorities requested and are now provided with, systematic surveillance of OSCOUR® ED attendances for asthma by age group during poor quality events. This work shows the potential of real-time syndromic surveillance to enhance the public health response to air pollution incidents, even if real-time changes observed through syndromic surveillance data cannot be absolutely related to air pollution.

The increases in attendance levels for specified indicators, particularly asthma in children, provides an insight into not only the age groups affected, but also how the workload and case mix within EDs can rapidly change. Contemporaneous feedback may be given on the utility of health warnings issued which may aid in the targeting of advice to particular age groups and also the preparations made in EDs in terms of staffing and materials required.

Where increased ED attendances were observed during periods of no known changes in AQ, there is potential for further investigation of the potential causes. The identification of periods

For peer review only

of thunderstorm activity on the days of the highest asthma attendances reported here should be investigated further.

This study is the first example of the RAMMIE method being applied to a syndromic surveillance system outside the UK, identifying and highlighting increases in ED attendances during periods of known poor AQ. This work has illustrated the potential for RAMMIE to be applied to countries developing new syndromic surveillance systems, or without the infrastructure to support bespoke statistical developments. However, the limitations of this method must always be considered, where increased levels resulting in statistical alarms (either 2-week or 2-year) must be viewed alongside local intelligence and knowledge, not every alarms will be due to poor AQ, but the indicators can be used for monitoring the impact of AQ events on public health.

This work also promotes further collaboration between different countries to explore methods to harmonize syndromic surveillance systems. Other public health surveillance initiatives have been adopted across Europe to provide a means of reporting singularly comparable variables and statistics across several countries, including: the European monitoring of excess mortality for public health action (EuroMOMO) [46]; the European Influenza Surveillance Scheme (EISS) [47]; establishment of epidemic thresholds for influenza surveillance [48]; the European Antimicrobial Resistance Surveillance Network (EARS-net) [49]; harmonised norovirus surveillance systems also exist [50, 51]. Within this study, although ED indicators were not entirely harmonized, they had been developed to be the most appropriate for each system and country. This work has also stimulated opportunities to explore other areas of public health that could be enhanced using a multinational syndromic surveillance system in particular those due to non-infectious causes such as injury surveillance and these will be addressed in future work.

For peer review only

The apparent difference in the noise to signal ratio between OSCOUR® and EDSSS i.e. background variation was likely due to the size of each respective network. Peaks of abnormal activity were easier to identify in OSCOUR® and therefore future work within PHE is currently focusing on expanding the EDSSS to improve its geographical representativeness and increase the attendance numbers thereby reducing the noise to signal ratio.

The potential for the harmonisation of syndromic surveillance across national borders is also clear, with opportunities to build on local experience to bring international public health benefits.

For peer review only

Acknowledgements

We acknowledge the contribution and support from the ED clinicians and Trust staff; the on-going support of the Royal College of Emergency Medicine; the technical support provided by EMIS Health and L2S2 Ltd in developing the EDSSS system in England and Northern Ireland. We also acknowledge the contribution from the ED structures and clinicians involved in the OSCOUR® network in France; the on-going support of the Federation of the Regional Observatories of Emergencies; the Scientific Society of Emergency Medicine; the air and climate team of Santé publique France and the Regional Unit of Santé publique France in Paris-Ile-de-France region.

We acknowledge and thank Sylvia Medina (Santé Publique France) for their professional input and critical revision of this manuscript.

HEH receives support from the National Institute for Health Research Health Protection Research Unit (NIHR HPRU) in Gastrointestinal Infections. AJE and GES receive support from the National Institute for Health Research Health Protection Research Unit (NIHR HPRU) in Emergency Preparedness and Response. The views expressed are those of the author(s) and not necessarily those of the NHS, the NIHR, the Department of Health or Public Health England.

Ethics

Ethical approval for this work was not required. The anonymised EDSSS health data used in this study were routinely collected at part of the public health function of PHE. The collection and analysis of data provided by the OSCOUR network in the frame of public health surveillance and epidemiological studies has been authorized by the French National Commission for Data protection and Liberties (CNIL).

Funding

For peer review only

This research received no specific grant from any funding agency, commercial or not-for-profit sectors. This surveillance is undertaken as part of the national surveillance functions of Public Health England and Santé Publique France.

Conflicts of interest

None

Contributor statement

HEH contributed to the study design, prepared the ED data for England and Northern Ireland, completed the statistical analyses, drafted the manuscript and provided critical revision and final approval of the manuscript.

RM contributed to the study design, completed the statistical analyses, drafted the manuscript and provided critical revision and final approval of the manuscript.

AF contributed to the study design, prepared the ED data for France and provided critical revision and final approval of the manuscript.

CCS contributed to the study design, critical revision and final approval of the manuscript.

AD contributed to the study design, prepared the air quality data and provided critical revision and final approval of the manuscript.

TCH contributed to the study design, critical revision and final approval of the manuscript.

GES contributed to the study design, critical revision and final approval of the manuscript.

AJE contributed to the study design, critical revision and final approval of the manuscript.

Data sharing statement

Additional data are not available for sharing.

For peer review only

References

1. World Health Organisation. Air Quality Guidelines Global Update 2005. Particulate matter, ozone, nitrogen dioxide and sulfur dioxide: WHO2006.
2. Public Health England. Public Health Outcomes Framework: 3.01 - Fraction of mortality attributable to particulate air pollution. 2017. http://www.phoutcomes.info/public-health-outcomes-framework#page/6/gid/1000043/pat/15/par/E92000001/ati/6/are/E12000007/iid/30101/age/230/sex/4. Accessed 30/03/2017.
3. Pascal M, de Crouy Chanel P, Wagner V, Corso M, Tillier C, Bentayeb M, et al. The mortality impacts of fine particles in France. Sci Total Environ. 2016;571:416-25.
4. DIRECTIVE 2008/50/EC OF THE EUROPEAN PARLIAMENT AND OF THE COUNCIL of 21 May 2008 on ambient air quality and cleaner air for Europe, 2008/50/EC (2008).
5. Air quality guidelines. Global update 2005. Particulate matter, ozone, nitrogen dioxide and sulfur dioxide, (2006).
6. World Air Quality Index project. Air Pollution in the World: Real time Air Quality map. 2016. http://waqi.info/. Accessed 01/03/2016.
7. CITEAIR. Air Quality in Europe. Compare the current air quality in different European Cities. 2007. http://www.airqualitynow.eu/. Accessed 01/013/2016.
8. Department for Environment Food & Rural Affairs. UK-AIR: Information Resource. 2016. http://uk-air.defra.gov.uk/. Accessed 18 March 2016.
9. Department for Environment Food & Rural Affairs. Daily Air Quality Index. 2013. http://uk-air.defra.gov.uk/air-pollution/daqi. Accessed 18 March 2016.
10. Triple S Project. Assessment of syndromic surveillance in Europe. Lancet. 2011;378:1833-4.
11. Hughes HE, Morbey R, Hughes TC, Locker TE, Shannon T, Carmichael C, et al. Using an Emergency Department Syndromic Surveillance System to investigate the impact of extreme cold weather events. Public Health. 2014;128:628-35.
12. Josseran L, Caillere N, Brun-Ney D, Rottner J, Filleul L, Brucker G, et al. Syndromic surveillance and heat wave morbidity: a pilot study based on emergency departments in France. BMC Med Inform Decis Mak. 2009;9:doi: 10.1186/472-6947-9-14.
13. Elliot AJ, Hughes HE, Hughes TC, Locker TE, Brown R, Sarran C, et al. The impact of thunderstorm asthma on emergency department attendances across London during July 2013. Emerg Med J. 2013.
14. Smith GE, Bawa Z, Macklin Y, Morbey R, Dobney A, Vardoulakis S, et al. Using real-time syndromic surveillance systems to help explore the acute impact of the air pollution incident of March/April 2014 in England. Environ Res. 2015;136:500-4.
15. Johnson J, Ginsberg M. Monitoring health effects of wildfires using the biosense system. MMWR Wkly. 2008;57.
16. Rappold AG, Stone SL, Cascio WE, Neas LM, Kilaru VJ, Carraway MS, et al. Peat bog wildfire smoke exposure in rural North Carolina is associated with cardiopulmonary emergency department visits assessed through syndromic surveillance. Environ Health Perspect. 2011;119:1415-20.
17. Le Monde. Pollution : plus de 30 départements en alerte, transports gratuits en Ile-de-France. 2014. http://www.lemonde.fr/planete/article/2014/03/14/pollution-plus-de-30-departements-en-alerte-transports-gratuits-en-ile-de-france_4382974_3244.html. Accessed 26/5/16.
18. RT news. London to be ‘smogged’ by polluted Paris. 2014. https://www.rt.com/uk/154368-london-smog-pollution-paris/. Accessed 26/5/16.
19. BBC. UK air pollution: How bad is it? 2014. http://www.bbc.co.uk/news/uk-26851399. Accessed 26/5/16.
20. Airparif. Airparif: Air quality monitoring network. 2016. http://www.airparif.asso.fr/en/. Accessed 18 March 2016.

21. Affairs DfEFR. Data Selector. https://uk-air.defra.gov.uk/data/data_selector. Accessed 19/07/2016.
22. Airparif. Téléchargements: Results pollutant. http://www.airparif.asso.fr/telechargement/telechargement-polluant. Accessed 06/07/2016.
23. Elliot AJ, Hughes HE, Hughes TC, Locker TE, Shannon T, Heyworth J, et al. Establishing an emergency department syndromic surveillance system to support the London 2012 Olympic and Paralympic Games. Emerg Med J. 2012;29:954-60.
24. NHS digital. Accident and Emergency Diagnosis Tables. 2013. http://www.datadictionary.nhs.uk/web_site_content/supporting_information/clinical_coding/accident_and_emergency_diagnosis_tables.asp?shownav=1. Accessed 1 November 2013 2013.
25. World Health Organisation. International Classification of Disease (ICD). 2010. http://www.who.int/classifications/icd/en/. Accessed 1/11/13.
26. International Health Terminology Standards Development Organisation. SNOMED CT. 2012. http://www.ihtsdo.org/snomed-ct/. Accessed 30/3/13.
27. Caserio-Schonemann C, Bousquet V, Fouillet A, Henry V. Le système de surveillance syndromique SurSaUD®. Bulletin Epidémiologique Hebdomadaire. 2014:38-44.
28. Fouillet A, Bousquet V, Pontais I, Gallay A, Schönemann CC. The French Emergency Department OSCOUR Network: Evaluation After a 10-year Existence. Online Journal of Public Health Informatics. 2015;7:e74.
29. Morbey RA, Elliot AJ, Charlett A, Verlander NQ, Andrews N, Smith GE. The application of a novel 'rising activity, multi-level mixed effects, indicator emphasis' (RAMMIE) method for syndromic surveillance in England. Bioinformatics. 2015;31:3660-5.
30. Stata version 13.1. College Station, TX 77845 USA: Stata Corporation; 2013.
31. Alhanti BA, Chang HH, Winquist A, Mulholland JA, Darrow LA, Sarnat SE. Ambient air pollution and emergency department visits for asthma: a multi-city assessment of effect modification by age. J Expos Sci Environ Epidemiol. 2016;26:180-8.
32. Elliot AJ, Smith S, Dobney A, Thornes J, Smith GE, Vardoulakis S. Monitoring the effect of air pollution episodes on health care consultations and ambulance call-outs in England during March/April 2014: A retrospective observational analysis. Environmental Pollution. 2016;214:903-11.
33. EUMETSAT. Severe thunderstorms over parts of France. 2014. http://www.eumetsat.int/website/home/Images/ImageLibrary/DAT_2238698.html. Accessed 25/5/16.
34. RFI. Storms to return to France after night of hail, rain, thunder, lightning. 2014. http://en.rfi.fr/visiting-france/20140609-storms-return-france-after-night-hail-rain-lightning-damage. Accessed 25/5/16.
35. MetOffice. UK Climate, Climate Summaries, July 2014. 2015. http://www.metoffice.gov.uk/climate/uk/summaries/2014/july. Accessed 27/5/16 2016.
36. Dabrera G, Murray V, Emberlin J, Ayres JG, Collier C, Clewlow Y, et al. Thunderstorm asthma: an overview of the evidence base and implications for public health advice. QJM. 2013;106:207-17.
37. Wallis DN, Webb J, Brooke D, Brookes B, Brown R, Findlay A, et al. A major outbreak of asthma associated with a thunderstorm: experience of accident and emergency departments and patients' characteristics. BMJ. 1996;312:601-4.
38. Hajat S, Goubet SA, Haines A. Thunderstorm-associated asthma: the effect on GP consultations. Br J Gen Pract. 1997;47:639-41.
39. BBC. 'Thunderstorm asthma' deaths in Melbourne rise to eight. 2016. http://www.bbc.co.uk/news/world-australia-38121579.
40. Julious SA, Osman LM, Jiwa M. Increases in asthma hospital admissions associated with the end of the summer vacation for school-age children with asthma in two cities from England and Scotland. Public Health. 2007;121:482-4.

For peer review only

41. Fleming DM, Cross KW, Sunderland R, Ross AM. Comparison of the seasonal patterns of asthma identified in general practitioner episodes, hospital admissions, and deaths. Thorax. 2000;55:662-5.
42. Lincoln D, Morgan G, Sheppeard V, Jalaludin B, Corbett S, Beard J. Childhood asthma and return to school in Sydney, Australia. Public Health. 2006;120:854-62.
43. Institut de Veille Sanitaire. Surveillance syndromique SurSaUD®: Bulletin du réseau OSCOUR®: Institut de Veille Sanitaire2014 1 October 2014 Contract No.: 496.
44. Health Protection Agency. Emergency Department Syndromic Surveillance System Bulletin: Health Protection Agency, Team R-tSS;2014 23 March 2014.
45. McLaren J, Williams ID. The impact of communicating information about air pollution events on public health. Sci Total Environ. 2015;538:478-91.
46. EuroMOMO. European monitoring of excess mortality for public health action. 2017. http://www.euromomo.eu/index.html. Accessed 30/03/2017.
47. Paget J, Marquet R, Meijer A, van der Velden K. Influenza activity in Europe during eight seasons (1999–2007): an evaluation of the indicators used to measure activity and an assessment of the timing, length and course of peak activity (spread) across Europe. BMC Infect Dis. 2007;7:1-7.
48. Vega T, Lozano JE, Meerhoff T, Snacken R, Mott J, Ortiz de Lejarazu R, et al. Influenza surveillance in Europe: establishing epidemic thresholds by the Moving Epidemic Method. Influenza Other Respir Viruses. 2013;7:546-58.
49. European Centre for Disease Prevention and Control. European Antimicrobial Resistance Surveillance Network (EARS-Net) - See more at: http://ecdc.europa.eu/en/activities/surveillance/EARS-Net/Pages/index.aspx#sthash.5U9aDN1w.dpuf. http://ecdc.europa.eu/en/activities/surveillance/EARS-Net/Pages/index.aspx. Accessed 8/6/14.
50. Vega E, Barclay L, Gregoricus N, Williams K, Lee D, Vinjé J. Novel Surveillance Network for Norovirus Gastroenteritis Outbreaks, United States. Emerg Infect Dis. 2011;17:1389-95.
51. National Institute for Public Health and the Environment R. NoroNet. http://www.rivm.nl/en/Topics/N/NoroNet. Accessed 8/6/16.

For peer review only

Figure 1: Calculated mean daily PM value and corresponding Daily Air Quality Index band, by day during 2014 in London a. $PM_{2.5}$, b. PM_{10}: Paris c. $PM_{2.5}$, d. PM_{10}.

Figure 2: Mean emergency department attendances by day of week, 27 February 2014 – 1 October 2014, by syndromic indicators, London reported to EDSSS (a,c,e) and Paris reported to OSCOUR® (b,d,f).

Figure 3: Daily percentages of London ED attendances for syndromic surveillance indicators of A-B; asthma, C-D; difficulty breathing and E-F; myocardial ischaemia, selected age groups, with statistical alarms, reported to EDSSS.

Figure 4: Daily percentages of Paris ED attendances for syndromic surveillance indicators of A-B; asthma, C-D; difficulty breathing and E-F; myocardial ischaemia, selected age groups, with statistical alarms, reported to OSCOUR®.

For peer review only

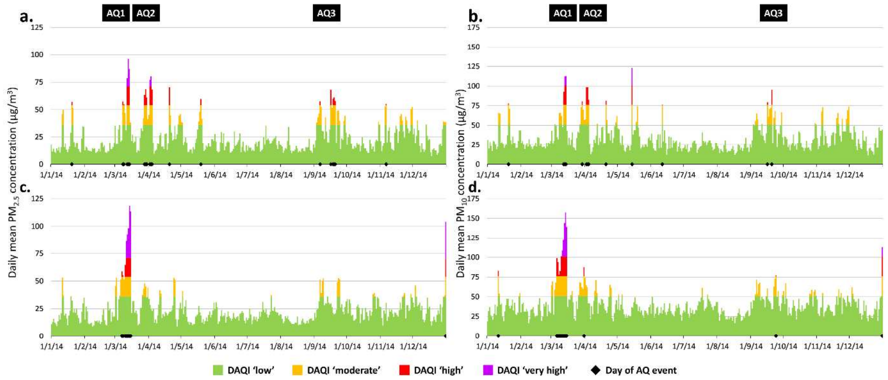

Figure 1: Calculated mean daily PM value and corresponding Daily Air Quality Index band, by day during 2014 in London a. PM2.5, b. PM10: Paris c. PM2.5, d. PM10.

599x399mm (300 x 300 DPI)

iew only

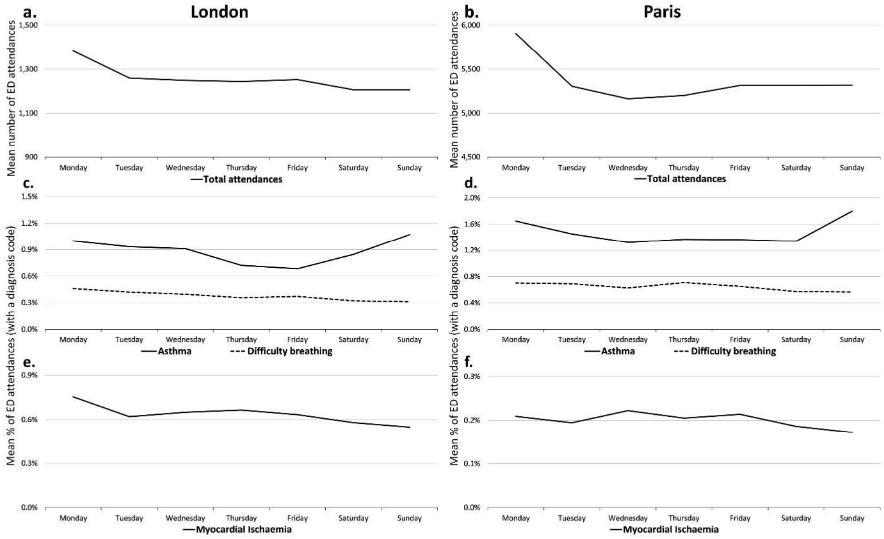

Figure 2: Mean emergency department attendances by day of week, 27 February 2014 – 1 October 2014, by syndromic indicators, London reported to EDSSS (a,c,e) and Paris reported to OSCOUR® (b,d,f).

599x399mm (300 x 300 DPI)

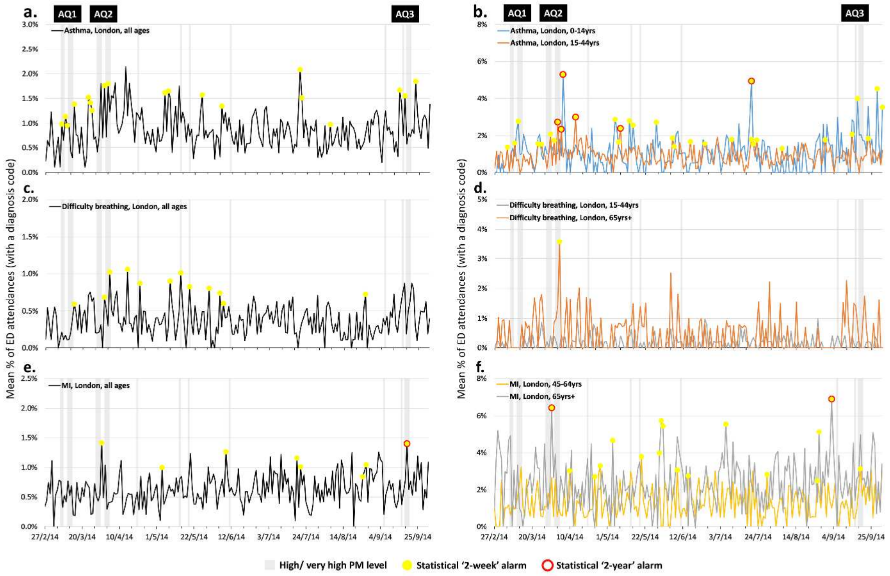

Figure 3: Daily percentages of London ED attendances for syndromic surveillance indicators of A-B; asthma, C-D; difficulty breathing and E-F; myocardial ischaemia, selected age groups, with statistical alarms, reported to EDSSS.

599x399mm (300 x 300 DPI)

ew only

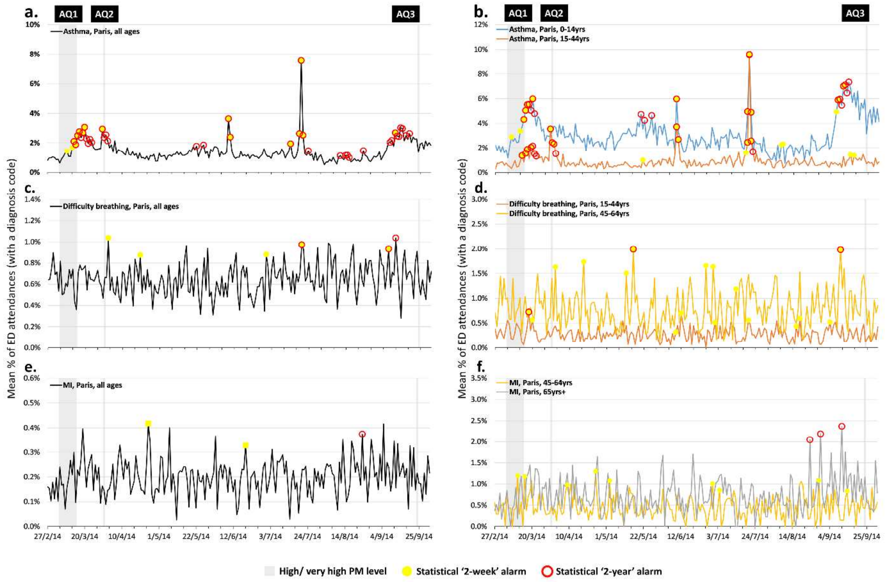

Figure 4: Daily percentages of Paris ED attendances for syndromic surveillance indicators of A-B; asthma, C-D; difficulty breathing and E-F; myocardial ischaemia, selected age groups, with statistical alarms, reported to OSCOUR®.

599x399mm (300 x 300 DPI)

For peer review only

STROBE Statement—checklist of items that should be included in reports of observational studies

	Item No	Recommendation
**Title and abstract**	1	(*a*) Indicate the study's design with a commonly used term in the title or the abstract<br><br>**A retrospective comparison of emergency department syndromic surveillance data during air pollution episodes across London and Paris in 2014 [p1]**
		(*b*) Provide in the abstract an informative and balanced summary of what was done and what was found<br><br>**Introduction**<br>Poor air quality (AQ) is a global public health issue and AQ events can span across countries. Using emergency department (ED) syndromic surveillance from England and France, we describe changes in human health indicators during periods of particularly poor AQ in London and Paris during 2014.<br>**Methods**<br>Using daily AQ data for 2014, we identified 3 periods of poor AQ affecting both London and Paris. Anonymised near real-time ED attendance syndromic surveillance data from EDs across England and France were used to monitor the health impact of poor AQ.<br>Using the routine English syndromic surveillance detection methods, increases in selected ED syndromic indicators (asthma, difficulty breathing and myocardial ischaemia), in total and by age, were identified and compared to periods of poor AQ in each city. Retrospective Wilcoxon-Mann-Whitney tests were used to identify significant increases in ED attendance data on days with (and up to 3 days following) poor AQ.<br>**Results**<br>Almost 1.5 million ED attendances were recorded during the study period (27/2/14-1/10/14). Significant increases in ED attendances for asthma were identified around periods of poor AQ in both cities, especially in children (0-14yrs). Some variation was seen in Paris with a rapid increase during the first AQ period in asthma attendances amongst children (0-14yrs), whereas during the second period the increase was greater in adults.<br>**Discussion**<br>This work demonstrates the public health value of real-time syndromic surveillance in response to air pollution incidents, and the potential for further cross-border harmonisation to provide Europe-wide early alerting to health impacts. **[p2/3]**
**Introduction**		
Background/rationale	2	Explain the scientific background and rationale for the investigation being reported<br><br>Air pollution has negative impacts on human health. Short term exposure to poor air quality can affect lung function, including exacerbating asthma symptoms, and is associated with other acute deteriorations in respiratory and cardiovascular health [1]. Similar health effects have also been reported due to long term exposure, with exposure to ambient air pollution associated with lung cancer and chronic respiratory and cardiovascular conditions [1]. In addition to illness within the community and increased need for health care, air pollution is also associated with increased

For peer review only - http://bmjopen.bmj.com/site/about/guidelines.xhtml

		mortality, with an estimated 4.7% of deaths in the England attributed to air pollution [2] and 9% of deaths in France attributed to PM2.5 [3]. **[p4]**
Objectives	3	State specific objectives, including any prespecified hypotheses<br><br>During March and early April 2014 there was a period of widespread poor AQ across Europe. In particular, the urban conurbations of London (England) and Paris (France) were affected by high temperatures, Saharan dust and industrial emissions, resulting in widespread media attention [17-19]. Here, we use routine emergency department (ED) syndromic surveillance data collected across London and Paris during poor AQ periods throughout 2014 to investigate the compatibility of the two countries' ED syndromic surveillance systems and estimate describe the public health impact and associated short-term changes in health care seeking behaviour for selected respiratory and cardiac syndromes across different age groups. **[p5]**
**Methods**		
Study design	4	Present key elements of study design early in the paper<br><br>Here, we use routine emergency department (ED) syndromic surveillance data collected across London and Paris during poor AQ periods throughout 2014 to investigate the compatibility of the two countries' ED syndromic surveillance systems and estimate describe the public health impact and associated short-term changes in health care seeking behaviour for selected respiratory and cardiac syndromes across different age groups. **[p5]**
Setting	5	Describe the setting, locations, and relevant dates, including periods of recruitment, exposure, follow-up, and data collection<br><br>The area studied here has been limited to London and the whole Paris region (Île-de-France), rather than a country level. **[p6]**<br><br>This investigation included EDSSS participating EDs within the London PHE Centre, which all fall within central London. **[p7]**<br><br>Aggregated, anonymised daily data for the Paris region were made available for this analysis. **[p7]**<br><br>An overall study period was defined as 27 February 2014 – 1 October 2014 (216 days), to encompass each period where poor AQ occurred in both London and Paris, including 7 days before and after the first and final AQ events identified (table 23). **[p10]**
Participants	6	(*a*) *Cohort study*—Give the eligibility criteria, and the sources and methods of selection of participants. Describe methods of follow-up<br>*Case-control study*—Give the eligibility criteria, and the sources and methods of case ascertainment and control selection. Give the rationale for the choice of cases and controls<br>*Cross-sectional study*—Give the eligibility criteria, and the sources and methods of selection of participants<br><br>The Emergency Department Syndromic Surveillance System (EDSSS), is a sentinel ED system coordinated by Public Health England (PHE), collecting anonymised data from participating EDs on a daily basis (data for the previous day 00:00 to 23:59 are

For peer review only - http://bmjopen.bmj.com/site/about/guidelines.xhtml

		transferred to PHE the following morning) [23]. Diagnosis coding in EDs in England was not standardised at the time of this investigation. Each ED had a list of diagnosis terms created locally which was available for selection in the patient attendance record. These diagnostic terms have associated codes linked to them with each ED using one of three codesets: Commissioning Data Set Accident and Emergency Diagnosis Tables[24], ICD-10 [25] or Snomed CT [26]. EDs eligible for inclusion in this study were defined as those reporting using ICD-10 [25] or Snomed CT [26] diagnosis coding systems which provide the level of detail required for the identification of the indicators of interest. **[p6/7]**<br><br>The French national ED syndromic surveillance system collects daily data from the Organisation de la Surveillance COordonnée des URgences (OSCOUR®) network of EDs, coordinated by Santé Publique France [27] (again, data for the previous day 00:00 to 23:59 are transferred and analysed the following morning, though OSCOUR® does allow for updates and delayed reporting, with around 15% of EDs reporting in the following 2 days[28]) [27]. All EDs reporting to OSCOUR® use ICD-10 for the coding of diagnoses selected in the patient attendance record [28]. **[p6/7]**
		(*b*) *Cohort study*—For matched studies, give matching criteria and number of exposed and unexposed<br>*Case-control study*—For matched studies, give matching criteria and the number of controls per case
Variables	7	Clearly define all outcomes, exposures, predictors, potential confounders, and effect modifiers. Give diagnostic criteria, if applicable<br><br>In England, the Department for Environment, Food and Rural Affairs monitors and reports on levels of air pollution using monitoring stations and provides health advice using the Daily Air Quality Index (DAQI) [9]. Air quality in the Paris region is monitored by Airparif and reported using the Citeair index [20]. **[p6]**<br><br>Syndromic indicators (asthma, difficulty breathing and myocardial ischaemia (MI) (table 1)) were selected from the comparable indicators already created for each system, based on clinical knowledge and experience of the potential health effects linked to air pollution and those used in previous syndromic surveillance work. **[p7]**
Data sources/ measurement	8*	For each variable of interest, give sources of data and details of methods of assessment (measurement). Describe comparability of assessment methods if there is more than one group<br><br>Both DAQI and Citeair systems monitor and report on multiple pollutants, however each index is reported using different methodology. Therefore the daily pollution levels across both London and Paris were standardised here, using the reported levels of particulate matter (PM2.5 and PM10). The city wide average value for each PM on each calendar day was calculated as a mean of the maximum values reported for each monitoring station on that day, in that city [21, 22]. Periods of poor AQ were then defined as those when either the calculated PM2.5 or PM10 average value corresponded to the DAQI index levels of 7-10, which are classified as 'high', to 'very high' (PM2.5 >=54 µg/m³ or PM10 >=76 µg/m³). At these levels people, including those with no pre-existing medical conditions, are advised to consider reducing their activity levels, particularly outdoors [8]. **[p6]**

For peer review only - http://bmjopen.bmj.com/site/about/guidelines.xhtml

		These syndromic surveillance indicators, which are routinely used in both EDSSS and OSCOUR® are an aggregation relevant diagnostic codes representing similar diagnostic terms and recorded in the patient record. Though 'diagnostic' information these diagnoses have potentially been made before any final confirmation and may be based on the symptoms presented, with no level of certainty indicated. The overall asthma and MI indicator groupings were very similar in each system, with the terms included all describing either asthma or myocardial ischaemic conditions. Differences were found in non-asthma difficulty breathing type indicators, where EDSSS included symptomatic wheeze/ difficulty breathing type diagnoses and OSCOUR® included dyspnoea/ respiratory failure diagnoses (table 2). Please note: not every code listed was reported by – or even available for selection – from every ED. More relevant codes may exist for each indicator than described here, however only codes reported to EDSSS/ OSCOUR® in this study are included). **[p7/8]**
Bias	9	Describe any efforts to address potential sources of bias<br>Not applicable
Study size	10	Explain how the study size was arrived at<br>An overall study period was defined as 27 February 2014 – 1 October 2014 (216 days). **[p10]**<br>Over the study period 1,436,163 ED attendances were recorded across both London and Paris. **[p11]**
Quantitative variables	11	Explain how quantitative variables were handled in the analyses. If applicable, describe which groupings were chosen and why<br><br>Both DAQI and Citeair systems monitor and report on multiple pollutants, however each index is reported using different methodology. Therefore the daily pollution levels across both London and Paris were standardised here, using the reported levels of particulate matter (PM2.5 and PM10). The city wide average value for each PM on each calendar day was calculated as a mean of the maximum values reported for each monitoring station on that day, in that city [21, 22]. Periods of poor AQ were then defined as those when either the calculated PM2.5 or PM10 average value corresponded to the DAQI index levels of 7-10, which are classified as 'high', to 'very high' (PM2.5 >=54 μg/m³ or PM10 >=76 μg/m³). At these levels people, including those with no pre-existing medical conditions, are advised to consider reducing their activity levels, particularly outdoors [8]. **[p6]**<br><br>Syndromic indicators (asthma, difficulty breathing and myocardial ischaemia (MI) (table 1)) were selected from the comparable indicators already created for each system, based on clinical knowledge and experience of the potential health effects linked to air pollution and those used in previous syndromic surveillance work. **[p7]**<br><br>For each syndromic surveillance system, attendances were aggregated by age group defined as 0-14, 15-44, 45-64 and 65 years and over. **[p8]**
Statistical methods	12	(*a*) Describe all statistical methods, including those used to control for confounding<br><br>We applied the routine syndromic surveillance statistical detection algorithm from England: the RAMMIE method (Rising Activity, Multi-level Mixed effects Indicator Emphasis [29]). **[p8]**

For peer review only

		RAMMIE routinely allows for the prioritisation of alarms to facilitate the identification of significant activity, however, this function was not used here to ensure that all statistically significant activity was identified, and not just those signals prioritised by RAMMIE. **[p9]**<br><br>In addition to the RAMMIE analysis, the Wilcoxon-Mann-Whitney non-parametric test was used to test for significant differences in the syndromic indicators during the 2014 study period, by age group between those days with a poor AQ and those without. To allow for the possibility of a delayed response, separate analyses were conducted incorporating lags of one to three days following a day of poor AQ. **[p9]**
		(*b*) Describe any methods used to examine subgroups and interactions<br><br>RAMMIE was applied to both English and French ED data, including to age specific data. **[p8/9]**<br><br>In addition to the RAMMIE analysis, the Wilcoxon-Mann-Whitney non-parametric test was used to test for significant differences in the syndromic indicators during the 2014 study period, by age group between those days with a poor AQ and those without. **[p9]**
		(*c*) Explain how missing data were addressed
		(*d*) *Cohort study*—If applicable, explain how loss to follow-up was addressed<br>*Case-control study*—If applicable, explain how matching of cases and controls was addressed<br>*Cross-sectional study*—If applicable, describe analytical methods taking account of sampling strategy<br><br>Not applicable
		(*e*) Describe any sensitivity analyses<br>Not applicable
**Results**		
Participants	13*	(a) Report numbers of individuals at each stage of study—eg numbers potentially eligible, examined for eligibility, confirmed eligible, included in the study, completing follow-up, and analysed<br>Not applicable
		(b) Give reasons for non-participation at each stage<br>Not applicable
		(c) Consider use of a flow diagram<br>Not applicable
Descriptive data	14*	(a) Give characteristics of study participants (eg demographic, clinical, social) and information on exposures and potential confounders<br>Not applicable
		(b) Indicate number of participants with missing data for each variable of interest<br>Not applicable
		(c) *Cohort study*—Summarise follow-up time (eg, average and total amount)<br>Not applicable
Outcome data	15*	*Cohort study*—Report numbers of outcome events or summary measures over time<br>Not applicable

For peer review only

		Case-control study—Report numbers in each exposure category, or summary measures of exposure<br>Not applicable
		Cross-sectional study—Report numbers of outcome events or summary measures<br>During 2014, several periods of poor AQ were identified where the ‘high’ or ‘very high’ air pollution thresholds for particulate matter (PM2.5 and/or PM10) had been breached in both London and Paris (figure 1). Periods of poor air quality in Paris were generally observed to be of a longer duration and with higher DAQI levels than in London, though more individual days of poor AQ were identified in London. Two main periods of poor AQ overlapped in these cities in mid-March (AQ1, the largest event in both locations and where transboundary dust from the Sahara contributed to the makeup of the particulate matter fraction [14]) and early April (AQ2, mainly in London, though a 1 day PM10 spike in Paris), with a third, less severe period during September occurring in both cities within a 7 day period (AQ3) (table 3). **[p10]**<br>Over the study period 1,436,163 ED attendances were recorded across both London and Paris (table 4). Total attendances were higher in Paris (1,163,353; from 58 EDs) than London (272,810; from 5 EDs, 3 using ICD-10, 2 using SnomedCT). A comparable level of diagnosis coding was included in each city with 79% of London attendances and 72% of Paris attendances including a clinical diagnosis code. **[p11]**
Main results	16	(*a*) Give unadjusted estimates and, if applicable, confounder-adjusted estimates and their precision (eg, 95% confidence interval). Make clear which confounders were adjusted for and why they were included<br><br>Results of RAMMIE testing are given in figures 3 & 4 and Wilcoxon-Mann-Whitney testing are in table 5, and these are described in the text **[p12-14]**:<br><br>Small increases in asthma attendances (all ages) in London EDs were observed following AQ1 (figure 3a). ED asthma attendances continued to increase during and immediately following AQ2. RAMMIE 2-week alarms were reported for the increases in asthma (all ages) immediately following AQ1 in London, indicating an attendance level higher than the previous 2 weeks. **[p12]**<br><br>Clear increases in ED attendances (all ages) for asthma occurred during both AQ1 and AQ2 in Paris (figure 4a). These increases were detected by RAMMIE as statistically significant in comparison to previous years (2-year alarm), as well as compared to the preceding 2 weeks (2-week alarm). **[p13]**<br><br>Wilcoxon-Mann-Whitney test results provide further evidence, alongside the descriptive epidemiology and RAMMIE results, that there is a strong associationbetween days of poor AQ and asthma attendances all ages and particularly in children 0-14 years (table 5)**[p14]**
		(*b*) Report category boundaries when continuous variables were categorized<br>Not applicable
		(*c*) If relevant, consider translating estimates of relative risk into absolute risk for a meaningful time period<br>Not applicable
Other analyses	17	Report other analyses done—eg analyses of subgroups and interactions, and sensitivity analyses<br><br>The observed increase of asthma attendances during the AQ2 episode in London was most

For peer review only - http://bmjopen.bmj.com/site/about/guidelines.xhtml

For peer review only

evident in children aged 0-14 years, and young adults (15-44 years) with each age group reaching a peak in attendances 1 to 2 days later (figure 3b). Asthma attendances for older adults showed no evidence of increase around periods of poor AQ (data not shown). **[p12]**

Clear increases in ED attendances (all ages) for asthma occurred during both AQ1 and AQ2 in Paris (figure 4a). These increases were detected by RAMMIE as statistically significant in comparison to previous years (2-year alarm), as well as compared to the preceding 2 weeks (2-week alarm). However, when broken down by age, the increase in asthma attendances in the 0-14 years age group occurred during AQ1, but not AQ2; while asthma attendances in young adults (15-44yrs) were greater during AQ2 than AQ1. No statistical alarms were observed for asthma in children around AQ2, though they were present for young adults (figure 4b). **[p13]**

Discussion

Key results — 18 — Summarise key results with reference to study objectives

We used two national ED syndromic surveillance systems to describe and compare the short-term changes in ED indicators during periods of poor AQ in two European capital cities. The AQ events reported here in Paris and London were related to the same pollutants (PM2.5/ PM10), and were very similar in terms of the dates and duration, and changes in public health outcomes in terms of ED attendances.

The most sensitive ED indicator during periods of poor AQ was asthma, with the impact most apparent up to 3 days after a day of poor AQ. The breakdown of attendances by age group revealed some differences, with the strongest associations overall seen between poor AQ and asthma attendances in children. **[p15]**

Limitations — 19 — Discuss limitations of the study, taking into account sources of potential bias or imprecision. Discuss both direction and magnitude of any potential bias

1. The use of percentage or ED visits (with a diagnosis code), as an indication of ED attendances (rather than actual numbers), as reported here may be impacted by the overall levels of ED attendances (and levels of diagnostic coding) on any one day **[p17]**

2. A limitation of the statistical methods used here is that the occurrence of previous events (e.g. poor AQ or weather systems) influencing the indicators were not identified or removed from the 2 years of historical data used as RAMMIE training data **[p18]**

3. This study focussed solely on particulate matter, though other pollutants impact on human health **[p18]**

4. The impact of health warnings and media reporting associated with actual and predicted periods of poor AQ could not be controlled for **[p18]**

5. It is important here to underline that variations of near real-time indicators are not easy to attribute directly to poor AQ. An absence of short term variation (e.g. MI in this study) cannot not be interpreted as a total lack of any longer term impact. Similarly, the identification of a significant increase in syndromic indicators reported here (e.g. asthma) has not formally accounted for other

For peer review only - http://bmjopen.bmj.com/site/about/guidelines.xhtml

		associated factors such as climatic conditions (e.g. weather and allergens) or viral circulation **[p19]**
Interpretation	20	Give a cautious overall interpretation of results considering objectives, limitations, multiplicity of analyses, results from similar studies, and other relevant evidence<br><br>The most sensitive ED indicator during periods of poor AQ was asthma, with the impact most apparent up to 3 days after a day of poor AQ. The breakdown of attendances by age group revealed some differences, with the strongest associations overall seen between poor AQ and asthma attendances in children. This finding was consistent with previous studies which have shown children to be more susceptible to exacerbation of asthma symptoms requiring health care in association with air pollution [31].**[p15]**<br><br>The investigation of individual AQ incidents demonstrated the potential for differing levels of impact on different age groups at different times. Though generally children were most affected by AQ, a large increase in adult asthma attendances was observed during and immediately following AQ2 in both London and Paris. Within England this increase in attendances around AQ2 has previously been described [32]. **[p15]**
Generalisability	21	Discuss the generalisability (external validity) of the study results<br><br>This work shows the potential of real-time syndromic surveillance to enhance the public health response to air pollution incidents, even if real-time changes observed through syndromic surveillance data cannot be absolutely related to air pollution. **[p19]**
**Other information**		
Funding	22	Give the source of funding and the role of the funders for the present study and, if applicable, for the original study on which the present article is based<br>Not applicable

*Give information separately for cases and controls in case-control studies and, if applicable, for exposed and unexposed groups in cohort and cross-sectional studies.

Note: An Explanation and Elaboration article discusses each checklist item and gives methodological background and published examples of transparent reporting. The STROBE checklist is best used in conjunction with this article (freely available on the Web sites of PLoS Medicine at http://www.plosmedicine.org/, Annals of Internal Medicine at http://www.annals.org/, and Epidemiology at http://www.epidem.com/). Information on the STROBE Initiative is available at www.strobe-statement.org.

For peer review only

For peer review only - http://bmjopen.bmj.com/site/about/guidelines.xhtml

BMJ Open

A retrospective observational study of emergency department syndromic surveillance data during air pollution episodes across London and Paris in 2014

Journal:	*BMJ Open*
Manuscript ID	bmjopen-2017-018732.R2
Article Type:	Research
Date Submitted by the Author:	26-Jan-2018
Complete List of Authors:	Hughes, Helen; Public Health England, Real-time Syndromic Surveillance; The Farr Institute @ HeRC,<br>Morbey, Roger; Public Health England, Real-time Syndromic Surveillance Team<br>Fouillet, Anne; Sante publique France, Syndromic Surveillance Unit<br>Caserio-Schönemann, Céline; Sante publique France, Syndromic Surveillance Unit<br>Dobney, Alec; Public Health England, Environmental Hazards and Emergencies Department<br>Hughes, Thomas; John Radcliffe Hospital, Emergency Department; Royal College of Emergency Medicine<br>Smith, Gillian; Public Health England, Real-time Syndromic Surveillance Team<br>Elliot, Alex; Health Protection Agency, Real-time Syndromic Surveillance Team
**Primary Subject Heading**:	Public health
Secondary Subject Heading:	Emergency medicine, Global health, Epidemiology
Keywords:	syndromic surveillance, air quality, particulate matter, emergency department, Asthma < THORACIC MEDICINE

SCHOLARONE™
Manuscripts

A retrospective observational study of emergency department syndromic surveillance data during air pollution episodes across London and Paris in 2014

H. E. Hughes[1,2,], R. Morbey[1], A. Fouillet[3], C. Caserio-Schönemann[3], A Dobney[4], T. C. Hughes[5,6], G. E. Smith[1], and A. J. Elliot[1]

1 Real-time Syndromic Surveillance Team, National Infection Service, Public Health England, Birmingham, UK;

2 Farr Institute at HeRC, Liverpool, UK

3 Syndromic Surveillance Unit, Santé publique France, the national public health agency, Saint-Maurice, France;

4 Environmental Hazards and Emergencies Department, Centre for Radiation, Chemical and Environmental Hazards, Public Health England, Birmingham, UK;

5 John Radcliffe Hospital, Oxford, UK;

6 The Royal College of Emergency Medicine, London, UK.

Address for correspondence:

Helen E Hughes, Real-time Syndromic Surveillance Team, Public Health England, 1st Floor, 5 St Philip's Place, Birmingham B3 2PW, UK

email: h.hughes7@liverpool.ac.uk

phone: +44 (0)844 225 3560 option 4 > option 2; fax: +44 (0)121 236 2215

Reprints will not be available from the author.

Word count: 3613

Key words: Syndromic surveillance, Emergency Department, Air quality, particulate matter, asthma, London, Paris

For peer review only

Abstract

Introduction

Poor air quality (AQ) is a global public health issue and AQ events can span across countries. Using emergency department (ED) syndromic surveillance from England and France, we describe changes in human health indicators during periods of particularly poor AQ in London and Paris during 2014.

Methods

Using daily AQ data for 2014, we identified 3 periods of poor AQ affecting both London and Paris. Anonymised near real-time ED attendance syndromic surveillance data from EDs across England and France were used to monitor the health impact of poor AQ.

Using the routine English syndromic surveillance detection methods, increases in selected ED syndromic indicators (asthma, difficulty breathing and myocardial ischaemia), in total and by age, were identified and compared to periods of poor AQ in each city. Retrospective Wilcoxon-Mann-Whitney tests were used to identify significant increases in ED attendance data on days with (and up to 3 days following) poor AQ.

Results

Almost 1.5 million ED attendances were recorded during the study period (27/2/14-1/10/14). Significant increases in ED attendances for asthma were identified around periods of poor AQ in both cities, especially in children (0-14yrs). Some variation was seen in Paris with a rapid increase during the first AQ period in asthma attendances amongst children (0-14yrs), whereas during the second period the increase was greater in adults.

Discussion

This work demonstrates the public health value of syndromic surveillance during air pollution incidents. There is potential for further cross-border harmonisation to provide Europe-wide

For peer review only

early alerting to health impacts and improve future public health messaging to health care services to provide warning of increases in demand.

Strengths and limitations of this study

- Routinely collected syndromic surveillance data from both England (London) and France (Paris) were analysed using similar health indicators
- A single statistical method, designed specifically for daily syndromic surveillance, was applied to data from both cities
- Air quality measurements were standardised across both cities, to overcome differences in the standard reporting from each
- Pollutants other than particulate matter were not included, though they may be responsible for impacts on human health
- We could not control for the potential effects of health warnings and media coverage on health care seeking behaviour

For peer review only

Introduction

Air quality

Air pollution has negative impacts on human health. Short term exposure to poor air quality can affect lung function, including exacerbating asthma symptoms, and is associated with other acute deteriorations in respiratory and cardiovascular health [1]. Similar health effects have also been reported due to long term exposure, with exposure to ambient air pollution associated with lung cancer and chronic respiratory and cardiovascular conditions [1]. In addition to illness within the community and increased need for health care, air pollution is also associated with increased mortality, with an estimated 4.7% of deaths in the England attributed to air pollution [2] and 9% of deaths in France attributed to $PM_{2.5}$ [3].

Air quality (AQ) monitoring identifies long term trends informing policy, provides evidence of meeting (or missing) statutory target levels and quantifies the impact of preventative measures [4, 5]. Daily AQ monitoring enables daily reporting of both actual and modelled AQ (predicting one or more days in advance), for whole countries and/or individual cities, as well as on a smaller scale around individual monitoring stations [6-8]. This information is increasingly easy to access through websites and apps and is often reported through the media, especially following formal health warnings [9].

Syndromic surveillance

Syndromic surveillance initially focussed on infectious diseases such as influenza but is increasingly being used for other non-infectious public health events. This type of surveillance uses real-time data from patient contacts with health care services (e.g. telephone helplines, general practice/ family doctors, or emergency departments). Patient contacts/ attendances are grouped by diagnoses/ symptoms creating syndromic indicators such as ‘respiratory’ or ‘gastrointestinal’, providing valuable information for public health action [10]. The use of emergency department (ED) data lends itself particularly well to the

For peer review only

syndromic surveillance of non-infectious public health events, with patients seeking attention for a range of acute conditions [11-13]. Previous investigation of periods of poor air quality have shown associated increases in health seeking behaviour as evidenced by syndromic surveillance, particularly for asthma and/ or difficulty breathing and heart failure [14-16], though not for myocardial infarction [16].

Aims

During March and early April 2014 there was a period of widespread poor AQ across Europe. In particular, the urban conurbations of London (England) and Paris (France) were affected by high temperatures, Saharan dust and industrial emissions, resulting in widespread media attention [17-19]. Here, we use routine ED syndromic surveillance data collected across London and Paris during poor AQ periods throughout 2014 to investigate the compatibility of the two countries' ED syndromic surveillance systems and describe the public health impact and associated short-term changes in health care seeking behaviour for selected respiratory and cardiac syndromes across different age groups.

For peer review only

Methods

Air quality data

The area studied here has been limited to London and the whole Paris region (Île-de-France), rather than a country level. In England, the Department for Environment, Food and Rural Affairs monitors and reports on levels of air pollution using monitoring stations and provides health advice using the Daily Air Quality Index (DAQI) [9]. Air quality in the Paris region is monitored by Airparif and reported using the Citeair index [20].

Both DAQI and Citeair systems monitor and report on multiple pollutants, however each index is reported using different methodology. Therefore the daily pollution levels across both London and Paris were standardised here, using the reported levels of particulate matter ($PM_{2.5}$ and PM_{10}). The city wide average value for each PM on each calendar day was calculated as a mean of the maximum values reported for each monitoring station on that day, in that city [21, 22]. Periods of poor AQ were then defined as those when the calculated $PM_{2.5}$ and/ or calculated PM_{10} average value corresponded to the DAQI index levels of 7-10, which are the particulate matter levels classified as 'high' to 'very high' ($PM_{2.5}$ >=54 μg/m³ and/ or PM_{10} >=76 μg/m³). At these levels people, including those with no pre-existing medical conditions, are advised to consider reducing their activity levels, particularly outdoors [8].

Emergency department syndromic surveillance data

The Emergency Department Syndromic Surveillance System (EDSSS), is a sentinel ED system coordinated by Public Health England (PHE), collecting anonymised data from participating EDs on a daily basis (data for the previous day 00:00 to 23:59 are transferred to PHE the following morning) [23]. Diagnosis coding in EDs in England was not standardised at the time of this investigation. Each ED had a list of diagnosis terms created locally which was available for selection in the patient attendance record. These diagnostic terms have

associated codes linked to them with each ED using one of three codesets: Commissioning Data Set (CDS) Accident and Emergency Diagnosis Tables [24], ICD-10 [25] or Snomed CT [26]. EDs eligible for inclusion in this study were defined as those reporting using ICD-10 or Snomed CT diagnosis coding systems which provide the level of detail required for the identification of the indicators of interest; EDs using the CDS coding system were not able to provide the coded diagnosis data in this detail. This investigation included 5 eligible EDSSS participating EDs in London (all located within the London PHE Centre).

The French national ED syndromic surveillance system collects daily data from the Organisation de la Surveillance COordonnée des URgences (OSCOUR®) network of EDs, coordinated by Santé Publique France [27] (data for the previous day 00:00 to 23:59 are transferred and analysed the following morning for 85% of attendances at participating OSCOUR® EDs. The OSCOUR® system allows for updates and delayed reporting, the missing 15% of ED attendances from OSCOUR® EDs are reported in the following 2 days [28]). All EDs reporting to OSCOUR® use ICD-10 for the coding of diagnoses selected in the patient attendance record [28]. Aggregated, anonymised daily data for the Paris region (including 58 eligible EDs) were made available for this analysis.

Epidemiological analysis

Syndromic indicators (asthma, difficulty breathing and myocardial ischaemia (MI) (table 1)) were selected from the comparable indicators already created for each system, based on clinical knowledge and experience of the potential health effects linked to air pollution and those used in previous syndromic surveillance work.

For peer review only

Table 1: Syndromic surveillance indicators included in the EDSSS (London) and OSCOUR® (Paris) emergency department systems and used in the study

EDSSS (London)	OSCOUR® (Paris)	Reported here as
Asthma	Asthme	Asthma
Wheeze/ difficulty breathing	Dyspnée/ Insuffisance respiratoire	Difficulty breathing
Myocardial ischaemia	Ischémie myocardique	Myocardial Ischaemia (MI)

These syndromic surveillance indicators, which are routinely used in both EDSSS and OSCOUR®, are an aggregation of relevant diagnostic codes representing similar diagnostic terms and available in the patient record. These 'diagnoses' may not be confirmed or final and may be based on the symptoms presented, with no level of certainty indicated. The overall asthma and MI indicator groupings were very similar in each system, with the terms included all describing either asthma or myocardial ischaemic conditions. Differences were found in non-asthma difficulty breathing type indicators; EDSSS included symptomatic wheeze/ difficulty breathing type diagnoses whereas OSCOUR® included dyspnoea/ respiratory failure diagnoses (table 2). Please note: not every code listed was reported by – or even available for selection from – every ED. More relevant codes may exist for each indicator than described here, however only codes reported to EDSSS/ OSCOUR® in this study are included. Though each system was found to include different codes and even numbers of codes within each indicator, they would identify most of the same patients for inclusion within the indicators used here.

For peer review only

Table 2: Diagnostic codes mapped to for syndromic surveillance indicators included in the EDSSS (London) and OSCOUR® (Paris) emergency department systems and used in the study.

EDSSS			OSCOUR	
**Indicator**	**Code system**	**Codes**	**Indicator**	**Codes (ICD-10)**
**Asthma**	ICD-10	J450, J459	**Asthme (Asthma)**	J45, J450, J451, J458, J459, J46
	Snomed CT	30352005, 31387002, 55570000, 57546000, 161527007, 182728008, 195967001, 266364000, 281239006, 304527002, 312453004, 370204008, 370218001, 370219009, 389145006, 401135008, 409663006, 425969006, 445427006, 201031000000108, 340901000000107, 589241000000104, 653751000000109		
**Difficulty breathing/ wheeze**	ICD-10	R06.0, R060, R062, R068	**Dyspnée/ Insuffisance respiratoire (Dyspnoea/ respiratory failure)**	J960, J961, J961+0, J961+1, J969, R060
	Snomed CT	9763007, 18197001, 23141003, 24612001, 55442000, 56018004, 58596002, 60845006, 62744007, 68095009, 70407001, 161941007, 161947006, 162891007, 162894004, 230145002, 233683003, 267036007, 301703002, 301826004, 307487006, 386813002,		

For peer review only

		427354000, 427679007, 442025000, 276191000000107, 498001000000107, 498011000000109, 502631000000100, 572661000000100, 755581000000101, 755591000000104, 755611000000107, 756081000000102		
**Myocardial ischemia**	ICD-10	I200, I209, I219, I2510	**Ischémie myocardique (Myocardial ischemia)**	I20, I200, I200+0, I201, I208, I209, I21, I210, I2100, I21000, I2108, I211, I2110, I21100, I2118, I2, I212, I2120, I21200, I2128, I213, I2130, I21300, I2138, I214, I2140, I21400, I2148, I219, I2190, I21900, I2198, I22, I220, I2200, I22000, I2208, I221, I2210, I22100, I2218, I228, I2280, I22800, I2288, I229, I2290, I22900, I2298, I23, I230, I231, I232, I233, I234, I235, I236, I238, I24, I240, I241, I248, I249, I25, I250, I251, I252, I253, I254, I255, I256, I258, I259
	Snomed CT	22298006, 48447003, 53741008, 54329005, 57054005, 59021001, 67682002, 73795002, 155308009, 194828000, 233819005, 233822007, 233843008, 394659003, 398274000, 401303003, 401314000, 414545008, 414795007, 671571000000105		

For peer review only

For each syndromic surveillance system, attendances were aggregated by age group defined as 0-14, 15-44, 45-64 and 65 years and over.

The epidemiological analysis of ED attendance data included construction of trends in attendances for each syndromic indicator, both for all ages and for each age group, and city. The daily percentage(s) of attendances for each indicator were calculated using the number of attendances within an indicator (numerator) and the daily number of total (all cause) attendances with a diagnosis code within each surveillance system (denominator).

Statistical analysis

The EDSSS and OSCOUR® are both live public health surveillance systems prospectively collecting data with automated contemporaneous statistical algorithms underpinning the detection of unusual activity. We applied the routine syndromic surveillance statistical detection algorithm from England: the RAMMIE method (Rising Activity, Multi-level Mixed effects Indicator Emphasis [29]). RAMMIE was applied to both English and French ED data, including to age specific data. Using RAMMIE two separate statistical thresholds were calculated: a '2-year' threshold (based on the previous 2 years of data) to identify significant activity compared to previous years, and a '2-week' threshold (based on the previous two weeks) to identify recent, statistically significant, increases in daily activity. RAMMIE routinely allows for the prioritisation of alarms to facilitate the identification of significant activity, however, this function was not used here to ensure that all statistically significant activity was identified, and not just those signals prioritised by RAMMIE.

To ensure that sufficient data were included here to cover each of the AQ events identified, a study period of a minimum of 7 days pre the first and 7 days post the final period of poor AQ identified in London/ Paris during 2014 was selected. A further period of 2 years of data prior to the first AQ event provided required baseline data for the RAMMIE method.

In addition to the RAMMIE analysis, the Wilcoxon-Mann-Whitney non-parametric test was used to test for significant differences in the syndromic indicators during the 2014 study period, by age group between those days with a poor AQ and those without. To allow for the possibility of a delayed response, separate analyses were conducted incorporating lags of one to three days following a day of poor AQ.

All analyses were undertaken using Stata v13.1 [30].

Results

Air quality events

During 2014, several periods of poor AQ were identified where the 'high' or 'very high' air pollution thresholds for particulate matter ($PM_{2.5}$ and/or PM_{10}) had been breached in both London and Paris (figure 1). Periods of poor air quality in Paris were generally observed to be of a longer duration and with higher DAQI levels than in London, though more individual days of poor AQ were identified in London. Two main periods of poor AQ overlapped in these cities in mid-March and early April. AQ1 was the largest event in both locations and where transboundary dust from the Sahara contributed to the makeup of the particulate matter fraction [14]. AQ2 was apparent mainly in London (though a 1 day PM_{10} spike in Paris was recorded). A third, less severe period occurred in both cities during September within a 7 day period (AQ3; table 3).

An overall study period was defined as 27 February 2014 – 1 October 2014 (216 days), to encompass each period where poor AQ occurred in both London and Paris, including 7 days before and after the first and final AQ events identified (table 3).

Table 3: Dates of poor air quality, coinciding in London and Paris during 2014

	AQ1	AQ2	AQ3	Total AQ days
**London**	08/03/14 - 14/03/14	28/03/14 - 04/04/14	16/09/14 - 20/09/14	15
**Paris**	06/03/14 - 15/03/14	31/03/14	24/09/14	12

ED attendances

Over the study period 1,436,163 ED attendances were recorded across both London and Paris (table 4). Total attendances were higher in Paris (1,163,353; from 58 EDs; >75% of all attendances[31]) than London (272,810; from 5 EDs, 3 using ICD-10, 2 using Snomed CT; <25% of attendances). A comparable level of diagnosis coding was included in each city with 79% of London attendances and 72% of Paris attendances including a clinical diagnosis code.

On a weekly basis, total ED attendances in both London and Paris showed similar trends, with a peak observed on a Monday. Examination of indicator trends illustrated that there were further similarities between EDSSS and OSCOUR® with highest levels of asthma attendances (as a percentage of attendances with a diagnosis code); and lowest levels of MI attendances, reported on Sundays (figure 2).

For peer review only

Table 4: Attendances recorded in EDs, by city, over the study period (27/02/14-01/10/14)

		ED Attendances			*Attendances with a diagnosis*			*Indicator attendances*		
**City**	**EDs**	**ICD10**	**Snomed**	**Total**	**ICD10**	**Snomed**	**Total**	**Asthma**	**Difficulty breathing**	**Myocardial ischaemia**
London	5*	115,539	157,271	272,810	81,980 (71%)	132,750 (84%)	214,730 (79%)	1,893 (0.9%)	812 (0.4%)	1,370 (0.6%)
Paris	58	1,163,353	-	1,163,353	840,309 (72%)	-	840,309 (72%)	12,374 (1.5%)	5,433 (0.6%)	1,685 (0.2%)

*1 small ED (which used ICD-10) stopped reporting to EDSSS on 10/09/2014. All 5 EDs were included in descriptive and RAMMIE analysis; 4 EDs that reported throughout were included in Wilcoxon-Mann-Whitney testing.

For peer review only

ED attendances during poor air quality periods

London ED attendances

Small increases in asthma attendances (all ages) in London EDs were observed following AQ1 (figure 3a). ED asthma attendances continued to increase during and immediately following AQ2. RAMMIE 2-week alarms were reported for the increases in asthma (all ages) immediately following AQ1 in London, indicating an attendance level higher than the previous 2 weeks. However, single 2-week alarms were not unusual in these data and were also observed during periods with no reported AQ issues. 2-year asthma alarms were not observed in the all ages asthma attendances data during the study period.

The observed increase of asthma attendances during the AQ2 episode in London was most evident in children aged 0-14 years, and young adults (15-44 years) with each age group reaching a peak in attendances 1 to 2 days later (figure 3b). Asthma attendances for older adults showed no evidence of increase around periods of poor AQ (data not shown).

An additional peak in asthma (all ages) attendances was observed on 20/7/14 (figure 3a), particularly in children (0-14yrs; figure 3b), though there was no poor AQ identified at that time. During early September increases in all age attendances for asthma, largely driven by child attendances (0-14yrs), were observed to have started prior to AQ3.

A small increase in difficulty breathing attendances (all ages) immediately following AQ2 (figure 3c), was most apparent in the older adults (65 and over years; figure 3d). This single day peak was the highest level seen in this age group, around double the usual level, though not significantly higher than historical data. Other age groups were not affected.

MI attendances were less common than asthma attendances in London EDs (table 4) and affected the adult age groups almost exclusively, as would be expected. Though a peak (resulting in both 2-week and 2-year alarm) in MI attendances was observed during AQ2,

For peer review only

particularly in those aged 65yrs and over, a similar peak also occurred in late September, several days prior to the AQ3. 2-week alarms occurred quite frequently throughout the year (figure 3e & f).

Paris ED attendances

Clear increases in ED attendances (all ages) for asthma occurred during both AQ1 and AQ2 in Paris (figure 4a) and were statistically significant (2-year and 2-week alarms). However, when broken down by age, the increase in asthma attendances in the 0-14 years age group occurred during AQ1, but not AQ2; while asthma attendances in young adults (15-44yrs) were greater during AQ2 than AQ1. No statistical alarms were observed for asthma in children around AQ2, though they were present for young adults (figure 4b).

The largest peak in asthma attendances was observed on 20/07/14, for all ages apart from 65yrs and over (data not shown), matching the spike seen in London, despite air quality not being identified as poor on that day. One further peak in asthma attendances, apparent in all ages and individual age groups, was observed on 9-10/6/14 (figure 4a & b). The observed peaks were not concomitant with any period of poor AQ in Paris, nor London.

Similar to London, an increase in asthma attendances was observed in Paris at the beginning of September, prior to AQ3, driven predominantly by children (0-14 years).

Difficulty breathing attendances in Paris were much lower than for asthma overall, with a single increase after AQ2 (figure 4c). Within the 15-44yrs age group there was, however an increase in difficulty breathing attendances following AQ1 (figure 4d).

Attendances for MI in Paris showed no evidence of increase in Paris during/ following days of poor AQ (figures 4e & f), though some statistical alarms were observed throughout the year, particularly a series of three 2-year alarms during late August and September in those aged 65 years and over.

For peer review only

Retrospective statistical analysis

Wilcoxon-Mann-Whitney test results provide further evidence, alongside the descriptive epidemiology and RAMMIE results, that there is a strong association between days of poor AQ and asthma attendances all ages and particularly in children 0-14 years (table 5). Furthermore, the statistical significances of the associations between asthma attendances and poor AQ were highest when modelled with a lag between the day of poor AQ and attendances; two days for London and three days for Paris. Though there was some evidence of increased attendances for difficulty breathing and MI in some age groups in London one day after poor AQ, these alarms were single significant values (rather than the grouping of significant asthma results by age group; figure 3d&f). These increased MI and difficulty breathing attendances in the day following poor AQ were not seen in the Paris data (figure 4d&f).

For peer review only

Table 5: Results of the Wilcoxon-Mann-Whitney test illustrating the standardised value (z value) and significance (P value) of syndromic indicators to days of poor air quality (including 1-3 day lag).

Indicator	City	lag (days)	all ages z value	all ages p value	0-14yrs z value	0-14yrs p value	15-44yrs z value	15-44yrs p value	46-64yrs z value	46-64yrs p value	65yrs+ z value	65yrs+ p value
Asthma	London	0	-0.227	0.8204	-2.857	**0.0043**	1.287	0.1982	1.077	0.2813	-1.009	0.3128
		1	-1.443	0.1490	-3.213	**0.0013**	0.556	0.5784	-0.791	0.4291	-1.026	0.3048
		2	-1.713	**0.0867**	-3.838	**0.0001**	0.787	0.4310	-0.558	0.5768	-1.438	0.1503
		3	-1.627	0.1038	-2.574	**0.0100**	-0.141	0.8876	-0.442	0.6586	0.816	0.4145
	Paris	0	-0.963	0.3356	-1.566	0.1173	0.529	0.5971	-0.624	0.5326	0.000	1.0000
		1	-2.035	**0.0419**	-2.576	**0.0100**	-0.330	0.7418	-1.582	0.1137	-0.354	0.7237
		2	-2.706	**0.0068**	-3.090	**0.0020**	-0.943	0.3454	-2.558	**0.0105**	-0.194	0.8464
		3	-3.049	**0.0023**	-3.201	**0.0014**	-1.797	**0.0724**	-2.77	**0.0056**	-0.756	0.4499
Difficulty Breathing	London	0	-0.055	0.9563	-0.963	0.3357	1.311	0.1898	-0.361	0.7181	-0.140	0.8889
		1	-1.261	0.2073	-2.975	**0.0029**	1.797	**0.0723**	0.445	0.6564	-0.728	0.4666
		2	-0.444	0.6573	-1.385	0.1659	0.223	0.8236	1.452	0.1464	-0.580	0.5620
		3	-1.552	0.1207	-1.236	0.2166	-0.695	0.4872	-0.01	0.9916	-0.296	0.7670
	Paris	0	-0.604	0.5459	0.031	0.9749	-0.585	0.5582	-0.736	0.4615	-0.147	0.8830
		1	-0.057	0.9547	-1.032	0.3021	-0.490	0.6242	0.603	0.5466	-0.078	0.9376
		2	-1.364	0.1725	-1.095	0.2735	-0.674	0.5004	-0.565	0.5722	-1.521	0.1283
		3	-1.144	0.2526	-0.528	0.5974	-0.942	0.3464	0.427	0.6697	-1.222	0.2217
MI	London	0	-0.605	0.5452	-	-	-0.084	0.9327	-1.275	0.2022	0.027	0.9787
		1	-0.588	0.5565	-	-	0.329	0.7421	-1.994	**0.0461**	0.374	0.7084
		2	-0.081	0.9354	-	-	-0.084	0.9327	-0.61	0.5419	0.053	0.9574
		3	-0.571	0.5680	-	-	0.544	0.5862	-1.415	0.1571	-0.695	0.4873
	Paris	0	-0.364	0.7158	0.546	0.5850	-1.257	0.2089	-0.089	0.9293	0.367	0.7138
		1	0.243	0.8082	0.546	0.5850	-1.257	0.2089	-0.022	0.9828	1.594	0.1110
		2	-0.331	0.7408	0.546	0.5850	-0.522	0.6016	-0.235	0.8141	0.635	0.5253
		3	-0.676	0.4992	0.546	0.5850	-0.578	0.5630	0.384	0.7011	-0.403	0.6872

Figures in **bold** are significant to the 90% significance level; those **bold and underlined** to the 95% significance level.

Discussion

Main findings

We used two national ED syndromic surveillance systems to describe and compare the short-term changes in ED indicators during periods of poor AQ in two European capital cities. The AQ events reported here in Paris and London were related to the same pollutants ($PM_{2.5}$/ PM_{10}), and were very similar in terms of the dates and duration, and changes in public health outcomes in terms of ED attendances.

The most sensitive ED indicator during periods of poor AQ was asthma, with the impact most apparent up to 3 days after a day of poor AQ. The breakdown of attendances by age group revealed some differences, with the strongest associations overall seen between poor AQ and asthma attendances in children. This finding was consistent with previous studies which have shown children to be more susceptible to exacerbation of asthma symptoms requiring health care in association with air pollution [32].

The investigation of individual AQ incidents demonstrated the potential for differing levels of impact on different age groups at different times. Though generally children were most affected by AQ, a large increase in adult asthma attendances was observed during and immediately following AQ2 in both London and Paris. Within England this increase in attendances around AQ2 has previously been described [33]. As the second period of poor AQ to occur in a short period of time, media coverage and the associated communication of health warning information and interventions put in place during AQ2 may have resulted in changes in behaviour which affected the levels of exposure of different age groups.

In addition to the increases observed during AQ periods, a sharp increase in asthma attendances (all ages) was observed in Paris on 9-10/06/14, and in both London and Paris on 20/07/14. These peaks did not coincide with any AQ event identified here, however, additional meteorological data (not presented) revealed periods of major thunderstorm

For peer review only

activity within each city at the time [34-36]. These findings match those previously reported, including from the EDSSS, describing the health effects of 'thunderstorm asthma', where sudden exacerbation of asthma symptoms results in increased health care seeking behaviour over a short time period [13, 37-40], possibly due to increased levels of pollen and fungal spores, though the mechanism has not yet been confirmed [37].

We also observed further increases in asthma attendances in both Paris and London towards the start of September. This increase was particularly evident in children and is likely linked to an annual 'back to school' increase in asthma type attendances in EDs during September [41-43].

Other syndromic indicators investigated showed little (difficulty breathing), to no (MI) association with the AQ incidents identified here.

Strengths and limitations

The OSCOUR® system includes greater representative coverage nationally, with more EDs participating than the sentinel EDSSS system (540 EDs across France were reporting to OSCOUR® [44]. While 34 EDs across England and Northern Ireland were reporting to EDSSS at 20 March 2014, the five reported here were located in London making the EDSSS more representative in London than at the national level [45]). The large number of OSCOUR® EDs reported here resulted in much more stable data from Paris, reducing background noise and allowing clearer differentiation of spikes/increases in attendances. The smaller number of attendances within the EDSSS data made identifying spikes 'harder', however the use of RAMMIE enables significant increases in attendances to be identified, even when not initially obvious [29].

Despite underlying differences in the method of data collection, with EDSSS taking a single snapshot of daily attendances and OSCOUR® allowing the initial snapshot data to be updated retrospectively, both systems reported over 70% completion of the clinical diagnosis

For peer review only

field making diagnostic data comparable. Furthermore, though these systems were developed individually, it was found that the syndromic indicators used within each system were similar, making comparisons of health impact possible. However, the EDSSS used a wheeze/ difficulty breathing indicator whereas OSCOUR® used a difficulty breathing/ respiratory failure indicator. This difference is, in part, likely to be related to the use of different clinical coding systems, with the identification of symptoms (e.g. wheeze) more difficult using ICD-10 (as used in France) than Snomed CT (used by some EDs in England).

The percentage or ED visits (with a diagnosis code), as an indication of ED attendances, as reported here (rather than actual numbers) may be impacted by the overall levels of ED attendances (and levels of diagnostic coding) on any one day. Though travel and outdoor activities are discouraged during AQ events, there are other factors which have a much greater impact on ED attendances (such as national and school holiday periods). The patterns and total numbers of attendances during 2014, including AQ periods, were not different from those seen in other years. The normal levels of overall ED attendances observed during periods of poor AQ, though travel was discouraged, contrasts with the reduced overall ED attendances in the English EDSSS seen during extreme cold weather when transportation is not physically possible for most people [11]. By using percentage of attendances the impact of events, such as periods of poor AQ, can be clearly seen in terms of changes in ED workload, such as changes in case mix and/ or age groups attending.

The levels of attendances for each indicator were different between cities, with respiratory indicators higher in Paris (asthma 1.5%, difficulty breathing 0.7%), than London (asthma 0.9%, difficulty breathing 0.4%) and MI attendances higher in London (0.6%) than in Paris (0.2%). This disparity in attendance levels between countries may be due to differences in diagnosis coding practices, clinical procedures used for treating patients (e.g. immediate transfer to cardiac care rather than ED for MI patients) or even areas of specialty for each ED (e.g. some London EDs are part of specialist heart care hospitals so may see more MI

For peer review only

patients). However, the trends observed within weeks were very similar in both systems, implying they are broadly comparable (figure 2).

A limitation of the statistical methods used here is that the occurrence of previous events (e.g. poor AQ or weather systems) influencing the indicators were not identified or removed from the 2 years of historical data used as RAMMIE training data. The potential inclusion of unrecognised events may impact on the RAMMIE model thresholds, though 2 years is considered sufficient for meaningful results (personal communication with R. Morbey).

This study focussed solely on particulate matter, though other pollutants impact on human health. The application of the DAQI levels to both London and Paris mean daily data allowed for an international comparison, based on days with higher than usual $PM_{2.5}$ and/ or PM_{10} specific to each city. The use of the highest daily $PM_{2.5}$/ PM_{10} values was considered, but these values were found to be at the high/ very high on the DAQI scale on the majority of days of 2014.

The impact of health warnings and media reporting associated with actual and predicted periods of poor AQ could not be controlled for here. The intention of health warnings, which are reported in the media, is to reduce the impact on human health, encouraging the public to reduce exposure as recommended [9, 46]. There were increases in asthma attendances in children during and following AQ1 in Paris in particular, though these younger age groups appeared unaffected during later events, whereas young adults were more greatly affected by AQ2. These differences of impact by age group in AQ2 may have been due to changes in behaviour of younger age groups so soon after AQ1 and subsequent reduced exposure to poor AQ, rather than a biological response observed in adults only. In addition to the impact of media reporting, France has introduced several other measures when air quality limit values are exceeded in major cities; speed limits, alternate driving days (to limit the number of cars

on the road) and free public transportation. The implementation of these measures could have had an impact on the results presented here.

It is important here to underline that variations of near real-time indicators are not easy to attribute directly to poor AQ. An absence of short term variation (e.g. MI in this study) cannot not be interpreted as a total lack of any longer term impact. Similarly, the identification of a significant increase in syndromic indicators reported here (e.g. asthma) has not formally accounted for other associated factors such as climatic conditions (e.g. weather and allergens) or viral circulation. Further time series analysis should be completed to control potential confounding factors.

Future work

This work has prompted the systematic investigation of asthma attendances by age group around AQ events in England and Northern Ireland, using the EDSSS. In France (following the March 2014 periods of poor AQ reported here), the health authorities requested and are now provided with, systematic surveillance of OSCOUR® ED attendances for asthma by age group during poor quality events. This work shows the potential of real-time syndromic surveillance to enhance the public health response to air pollution incidents, even if real-time changes observed through syndromic surveillance data cannot be absolutely related to air pollution. As the evidence base for the utility of syndromic surveillance during air pollution events increases, it is hoped that it will, in combination with environmental data, be used by authorities to provide public health messaging during future events: messages to the public to advise about risks and preventative measures, and to EDs and other health service providers about increases in patient numbers and changes to the case mix of patients attending.

The increases in attendance levels for specified indicators, particularly asthma in children, provides an insight into not only the age groups affected, but also how the workload and case mix within EDs can rapidly change. Contemporaneous feedback may be given on the utility

For peer review only

of health warnings issued which may aid in the targeting of advice to particular age groups and also the preparations made in EDs in terms of staffing and materials required.

Where increased ED attendances were observed during periods of no known changes in AQ, there is potential for further investigation of the potential causes. The identification of periods of thunderstorm activity on the days of the highest asthma attendances reported here should be investigated further.

This study is the first example of the RAMMIE method being applied to a syndromic surveillance system outside the UK, identifying and highlighting increases in ED attendances during periods of known poor AQ. This work has illustrated the potential for RAMMIE to be applied to countries developing new syndromic surveillance systems, or without the infrastructure to support bespoke statistical developments. However, the limitations of this method must always be considered, where increased levels resulting in statistical alarms (either 2-week or 2-year) must be viewed alongside local intelligence and knowledge, not every alarm will be due to poor AQ, but the indicators can be used for monitoring the impact of AQ events on public health.

This work also promotes further collaboration between different countries to explore methods to harmonize syndromic surveillance systems. Other public health surveillance initiatives have been adopted across Europe to provide a means of reporting singularly comparable variables and statistics across several countries, including: the European monitoring of excess mortality for public health action (EuroMOMO) [47]; the European Influenza Surveillance Scheme (EISS) [48]; establishment of epidemic thresholds for influenza surveillance [49]; the European Antimicrobial Resistance Surveillance Network (EARS-net) [50]; harmonised norovirus surveillance systems also exist [51, 52]. Within this study, although ED indicators were not entirely harmonized, they had been developed to be the most appropriate for each system and country. This work has also stimulated opportunities to explore other areas of

For peer review only

public health that could be enhanced using a multinational syndromic surveillance system in particular those due to non-infectious causes such as injury surveillance and these will be addressed in future work.

The apparent difference in the noise to signal ratio between OSCOUR® and EDSSS i.e. background variation was likely due to the size of each respective network. Peaks of abnormal activity were easier to identify in OSCOUR® and therefore future work within PHE is currently focusing on expanding the EDSSS to improve its geographical representativeness and increase the attendance numbers thereby reducing the noise to signal ratio.

The potential for the harmonisation of syndromic surveillance across national borders is also clear, with opportunities to build on local experience to bring international public health benefits.

For peer review only

Acknowledgements

We acknowledge the contribution and support from the ED clinicians and Trust staff; the on-going support of the Royal College of Emergency Medicine; the technical support provided by EMIS Health and L2S2 Ltd in developing the EDSSS system in England and Northern Ireland. We also acknowledge the contribution from the ED structures and clinicians involved in the OSCOUR® network in France; the on-going support of the Federation of the Regional Observatories of Emergencies; the Scientific Society of Emergency Medicine; the air and climate team of Santé publique France and the Regional Unit of Santé publique France in Paris-Ile-de-France region.

We acknowledge and thank Sylvia Medina (Santé Publique France) for their professional input and critical revision of this manuscript.

HEH receives support from the National Institute for Health Research Health Protection Research Unit (NIHR HPRU) in Gastrointestinal Infections. AJE and GES receive support from the National Institute for Health Research Health Protection Research Unit (NIHR HPRU) in Emergency Preparedness and Response. The views expressed are those of the author(s) and not necessarily those of the NHS, the NIHR, the Department of Health or Public Health England.

Ethics

Ethical approval for this work was not required. The anonymised EDSSS health data used in this study were routinely collected at part of the public health function of PHE. The collection and analysis of data provided by the OSCOUR network in the frame of public health surveillance and epidemiological studies has been authorized by the French National Commission for Data protection and Liberties (CNIL).

Funding

For peer review only

This research received no specific grant from any funding agency, commercial or not-for-profit sectors. This surveillance is undertaken as part of the national surveillance functions of Public Health England and Santé Publique France.

Conflicts of interest

None

Contributor statement

HEH contributed to the study design, prepared the ED data for England, completed the statistical analyses, drafted the manuscript and provided critical revision and final approval of the manuscript.

RM contributed to the study design, completed the statistical analyses, drafted the manuscript and provided critical revision and final approval of the manuscript.

AF contributed to the study design, prepared the ED data for France and provided critical revision and final approval of the manuscript.

CCS contributed to the study design, critical revision and final approval of the manuscript.

AD contributed to the study design, prepared the air quality data and provided critical revision and final approval of the manuscript.

TCH contributed to the study design, critical revision and final approval of the manuscript.

GES contributed to the study design, critical revision and final approval of the manuscript.

AJE contributed to the study design, critical revision and final approval of the manuscript.

Data sharing statement

Additional data are not available for sharing.

For peer review only

References

1. World Health Organisation. Air Quality Guidelines Global Update 2005. Particulate matter, ozone, nitrogen dioxide and sulfur dioxide: WHO2006.
2. Public Health England. Public Health Outcomes Framework: 3.01 - Fraction of mortality attributable to particulate air pollution. 2017. http://www.phoutcomes.info/public-health-outcomes-framework#page/6/gid/1000043/pat/15/par/E92000001/ati/6/are/E12000007/iid/30101/age/230/sex/4. Accessed 30/03/2017.
3. Pascal M, de Crouy Chanel P, Wagner V, Corso M, Tillier C, Bentayeb M, et al. The mortality impacts of fine particles in France. Sci Total Environ. 2016;571:416-25.
4. DIRECTIVE 2008/50/EC OF THE EUROPEAN PARLIAMENT AND OF THE COUNCIL of 21 May 2008 on ambient air quality and cleaner air for Europe, 2008/50/EC (2008).
5. Air quality guidelines. Global update 2005. Particulate matter, ozone, nitrogen dioxide and sulfur dioxide, (2006).
6. World Air Quality Index project. Air Pollution in the World: Real time Air Quality map. 2016. http://waqi.info/. Accessed 01/03/2016.
7. CITEAIR. Air Quality in Europe. Compare the current air quality in different European Cities. 2007. http://www.airqualitynow.eu/. Accessed 01/013/2016.
8. Department for Environment Food & Rural Affairs. UK-AIR: Information Resource. 2016. http://uk-air.defra.gov.uk/. Accessed 18 March 2016.
9. Department for Environment Food & Rural Affairs. Daily Air Quality Index. 2013. http://uk-air.defra.gov.uk/air-pollution/daqi. Accessed 18 March 2016.
10. Triple S Project. Assessment of syndromic surveillance in Europe. Lancet. 2011;378:1833-4.
11. Hughes HE, Morbey R, Hughes TC, Locker TE, Shannon T, Carmichael C, et al. Using an Emergency Department Syndromic Surveillance System to investigate the impact of extreme cold weather events. Public Health. 2014;128:628-35.
12. Josseran L, Caillere N, Brun-Ney D, Rottner J, Filleul L, Brucker G, et al. Syndromic surveillance and heat wave morbidity: a pilot study based on emergency departments in France. BMC Med Inform Decis Mak. 2009;9:doi: 10.1186/472-6947-9-14.
13. Elliot AJ, Hughes HE, Hughes TC, Locker TE, Brown R, Sarran C, et al. The impact of thunderstorm asthma on emergency department attendances across London during July 2013. Emerg Med J. 2013.
14. Smith GE, Bawa Z, Macklin Y, Morbey R, Dobney A, Vardoulakis S, et al. Using real-time syndromic surveillance systems to help explore the acute impact of the air pollution incident of March/April 2014 in England. Environ Res. 2015;136:500-4.
15. Johnson J, Ginsberg M. Monitoring health effects of wildfires using the biosense system. MMWR Wkly. 2008;57.
16. Rappold AG, Stone SL, Cascio WE, Neas LM, Kilaru VJ, Carraway MS, et al. Peat bog wildfire smoke exposure in rural North Carolina is associated with cardiopulmonary emergency department visits assessed through syndromic surveillance. Environ Health Perspect. 2011;119:1415-20.
17. Le Monde. Pollution : plus de 30 départements en alerte, transports gratuits en Ile-de-France. 2014. http://www.lemonde.fr/planete/article/2014/03/14/pollution-plus-de-30-departements-en-alerte-transports-gratuits-en-ile-de-france_4382974_3244.html. Accessed 26/5/16.
18. RT news. London to be ‘smogged’ by polluted Paris. 2014. https://www.rt.com/uk/154368-london-smog-pollution-paris/. Accessed 26/5/16.
19. BBC. UK air pollution: How bad is it? 2014. http://www.bbc.co.uk/news/uk-26851399. Accessed 26/5/16.
20. Airparif. Airparif: Air quality monitoring network. 2016. http://www.airparif.asso.fr/en/. Accessed 18 March 2016.

For peer review only

21. Affairs DfEFR. Data Selector. https://uk-air.defra.gov.uk/data/data_selector. Accessed 19/07/2016.
22. Airparif. Téléchargements: Results pollutant. http://www.airparif.asso.fr/telechargement/telechargement-polluant. Accessed 06/07/2016.
23. Elliot AJ, Hughes HE, Hughes TC, Locker TE, Shannon T, Heyworth J, et al. Establishing an emergency department syndromic surveillance system to support the London 2012 Olympic and Paralympic Games. Emerg Med J. 2012;29:954-60.
24. NHS digital. Accident and Emergency Diagnosis Tables. 2013. http://www.datadictionary.nhs.uk/web_site_content/supporting_information/clinical_coding/accident_and_emergency_diagnosis_tables.asp?shownav=1. Accessed 1 November 2013 2013.
25. World Health Organisation. International Classification of Disease (ICD). 2010. http://www.who.int/classifications/icd/en/.
26. International Health Terminology Standards Development Organisation. SNOMED CT. 2012. http://www.ihtsdo.org/snomed-ct/.
27. Caserio-Schonemann C, Bousquet V, Fouillet A, Henry V. Le système de surveillance syndromique SurSaUD®. Bulletin Epidémiologique Hebdomadaire. 2014:38-44.
28. Fouillet A, Bousquet V, Pontais I, Gallay A, Schönemann CC. The French Emergency Department OSCOUR Network: Evaluation After a 10-year Existence. Online Journal of Public Health Informatics. 2015;7:e74.
29. Morbey RA, Elliot AJ, Charlett A, Verlander NQ, Andrews N, Smith GE. The application of a novel 'rising activity, multi-level mixed effects, indicator emphasis' (RAMMIE) method for syndromic surveillance in England. Bioinformatics. 2015;31:3660-5.
30. Stata version 13.1. College Station, TX 77845 USA: Stata Corporation; 2013.
31. Santé publique France. Réseau OSCOUR® - Organisation de la surveillance coordonnée des urgences. 2015. http://invs.santepubliquefrance.fr/Espace-professionnels/Surveillance-syndromique-SurSaUD-R/Reseau-OSCOUR-R.
32. Alhanti BA, Chang HH, Winquist A, Mulholland JA, Darrow LA, Sarnat SE. Ambient air pollution and emergency department visits for asthma: a multi-city assessment of effect modification by age. J Expos Sci Environ Epidemiol. 2016;26:180-8.
33. Elliot AJ, Smith S, Dobney A, Thornes J, Smith GE, Vardoulakis S. Monitoring the effect of air pollution episodes on health care consultations and ambulance call-outs in England during March/April 2014: A retrospective observational analysis. Environmental Pollution. 2016;214:903-11.
34. EUMETSAT. Severe thunderstorms over parts of France. 2014. http://www.eumetsat.int/website/home/Images/ImageLibrary/DAT_2238698.html. Accessed 25/5/16.
35. RFI. Storms to return to France after night of hail, rain, thunder, lightning. 2014. http://en.rfi.fr/visiting-france/20140609-storms-return-france-after-night-hail-rain-lightning-damage. Accessed 25/5/16.
36. MetOffice. UK Climate, Climate Summaries, July 2014. 2015. http://www.metoffice.gov.uk/climate/uk/summaries/2014/july. Accessed 27/5/16 2016.
37. Dabrera G, Murray V, Emberlin J, Ayres JG, Collier C, Clewlow Y, et al. Thunderstorm asthma: an overview of the evidence base and implications for public health advice. QJM. 2013;106:207-17.
38. Wallis DN, Webb J, Brooke D, Brookes B, Brown R, Findlay A, et al. A major outbreak of asthma associated with a thunderstorm: experience of accident and emergency departments and patients' characteristics. BMJ. 1996;312:601-4.
39. Hajat S, Goubet SA, Haines A. Thunderstorm-associated asthma: the effect on GP consultations. Br J Gen Pract. 1997;47:639-41.
40. BBC. 'Thunderstorm asthma' deaths in Melbourne rise to eight. 2016. http://www.bbc.co.uk/news/world-australia-38121579.

For peer review only

41. Julious SA, Osman LM, Jiwa M. Increases in asthma hospital admissions associated with the end of the summer vacation for school-age children with asthma in two cities from England and Scotland. Public Health. 2007;121:482-4.
42. Fleming DM, Cross KW, Sunderland R, Ross AM. Comparison of the seasonal patterns of asthma identified in general practitioner episodes, hospital admissions, and deaths. Thorax. 2000;55:662-5.
43. Lincoln D, Morgan G, Sheppeard V, Jalaludin B, Corbett S, Beard J. Childhood asthma and return to school in Sydney, Australia. Public Health. 2006;120:854-62.
44. Institut de Veille Sanitaire. Surveillance syndromique SurSaUD®: Bulletin du réseau OSCOUR®: Institut de Veille Sanitaire2014 1 October 2014 Contract No.: 496.
45. Health Protection Agency. Emergency Department Syndromic Surveillance System Bulletin: Health Protection Agency, Team R-tSS;2014 23 March 2014.
46. McLaren J, Williams ID. The impact of communicating information about air pollution events on public health. Sci Total Environ. 2015;538:478-91.
47. EuroMOMO. European monitoring of excess mortality for public health action. 2017. http://www.euromomo.eu/index.html. Accessed 30/03/2017.
48. Paget J, Marquet R, Meijer A, van der Velden K. Influenza activity in Europe during eight seasons (1999–2007): an evaluation of the indicators used to measure activity and an assessment of the timing, length and course of peak activity (spread) across Europe. BMC Infect Dis. 2007;7:1-7.
49. Vega T, Lozano JE, Meerhoff T, Snacken R, Mott J, Ortiz de Lejarazu R, et al. Influenza surveillance in Europe: establishing epidemic thresholds by the Moving Epidemic Method. Influenza Other Respir Viruses. 2013;7:546-58.
50. European Centre for Disease Prevention and Control. European Antimicrobial Resistance Surveillance Network (EARS-Net) - See more at: http://ecdc.europa.eu/en/activities/surveillance/EARS-Net/Pages/index.aspx#sthash.5U9aDN1w.dpuf. http://ecdc.europa.eu/en/activities/surveillance/EARS-Net/Pages/index.aspx. Accessed 8/6/14.
51. Vega E, Barclay L, Gregoricus N, Williams K, Lee D, Vinjé J. Novel Surveillance Network for Norovirus Gastroenteritis Outbreaks, United States. Emerg Infect Dis. 2011;17:1389-95.
52. National Institute for Public Health and the Environment R. NoroNet. http://www.rivm.nl/en/Topics/N/NoroNet. Accessed 8/6/16.

For peer review only

Figure 1: Calculated mean daily PM value and corresponding Daily Air Quality Index band, by day during 2014 in London a. $PM_{2.5}$, b. PM_{10}: Paris c. $PM_{2.5}$, d. PM_{10}.

Figure 2: Mean emergency department attendances by day of week, 27 February 2014 – 1 October 2014, by syndromic indicators, London reported to EDSSS (a.total attendances, c. Asthma and Difficulty breathing,e Myocardial Ischaemia) and Paris reported to OSCOUR® (b. total attendances, d. Asthma and Difficulty breathing, f. Myocardial Ischaemia).

Figure 3: Daily percentages of London ED attendances for syndromic surveillance indicators of a. asthma all ages, b. asthma 0-14 and 15-44years, c. Difficulty breathing all ages, d. Difficulty breathing 15-44 and 65+ years, e. Myocardial Ischaemia all ages and f. Myocardial ischaemia 45-64 and 65+ years, with statistical alarms, reported to EDSSS.

Figure 4: Daily percentages of Paris ED attendances for syndromic surveillance indicators of a. asthma all ages, b. asthma 0-14 and 15-44years, c. Difficulty breathing all ages, d. Difficulty breathing 15-44 and 45-64 years, e. Myocardial Ischaemia all ages and f. Myocardial ischaemia 45-64 and 65+ years, with statistical alarms, reported to OSCOUR®.

For peer review only

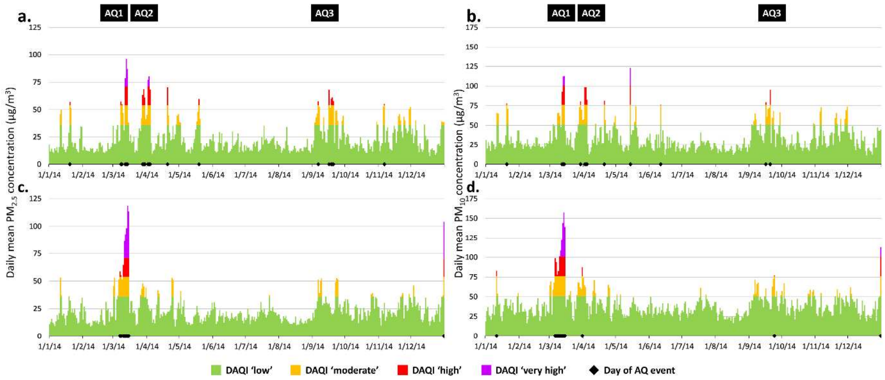

Figure 1: Calculated mean daily PM value and corresponding Daily Air Quality Index band, by day during 2014 in London a. PM2.5, b. PM10: Paris c. PM2.5, d. PM10.

599x399mm (300 x 300 DPI)

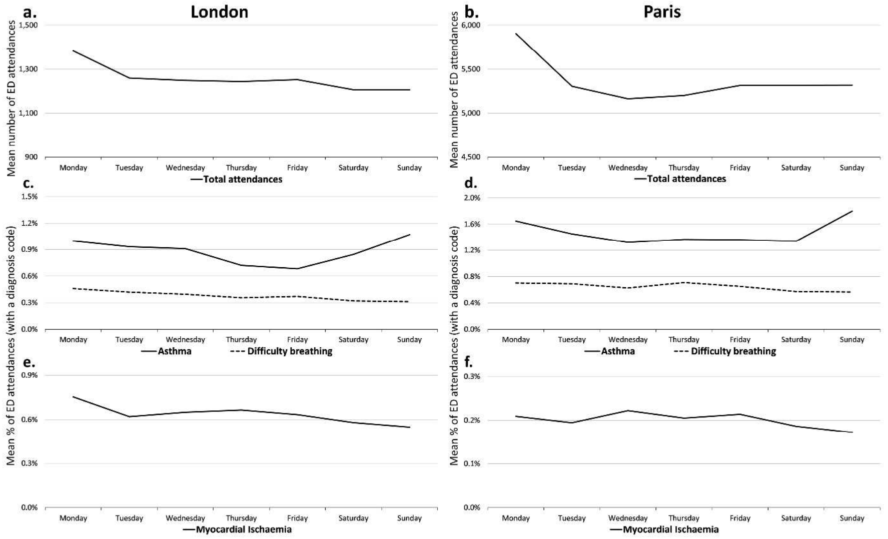

Figure 2: Mean emergency department attendances by day of week, 27 February 2014 – 1 October 2014, by syndromic indicators, London reported to EDSSS (a.total attendances, c. Asthma and Difficulty breathing,e Myocardial Ischaemia) and Paris reported to OSCOUR® (b. total attendances, d. Asthma and Difficulty breathing, f. Myocardial Ischaemia).

599x399mm (300 x 300 DPI)

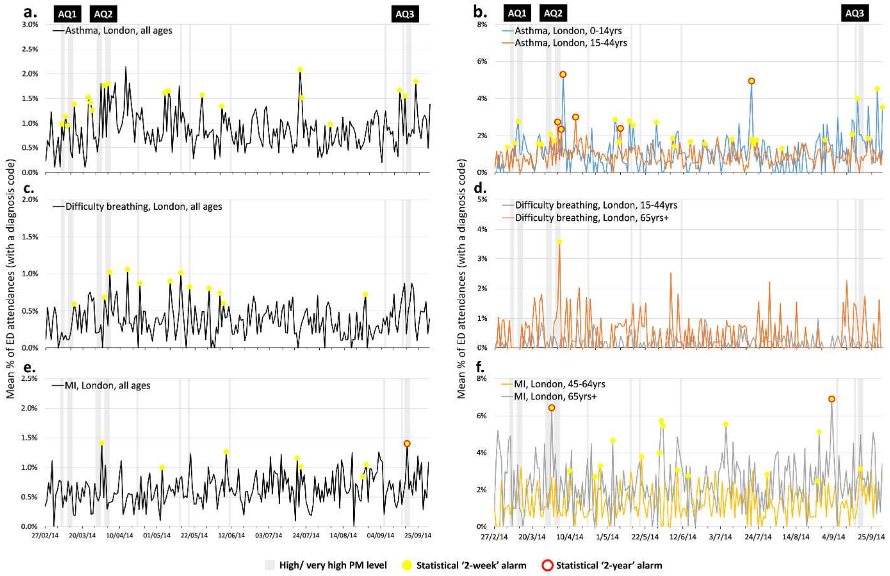

Figure 3: Daily percentages of London ED attendances for syndromic surveillance indicators of a. asthma all ages, b. asthma 0-14 and 15-44years, c. Difficulty breathing all ages, d. Difficulty breathing 15-44 and 65+ years, e. Myocardial Ischaemia all ages and f. Myocardial ischaemia 45-64 and 65+ years, with statistical alarms, reported to EDSSS.

599x399mm (300 x 300 DPI)

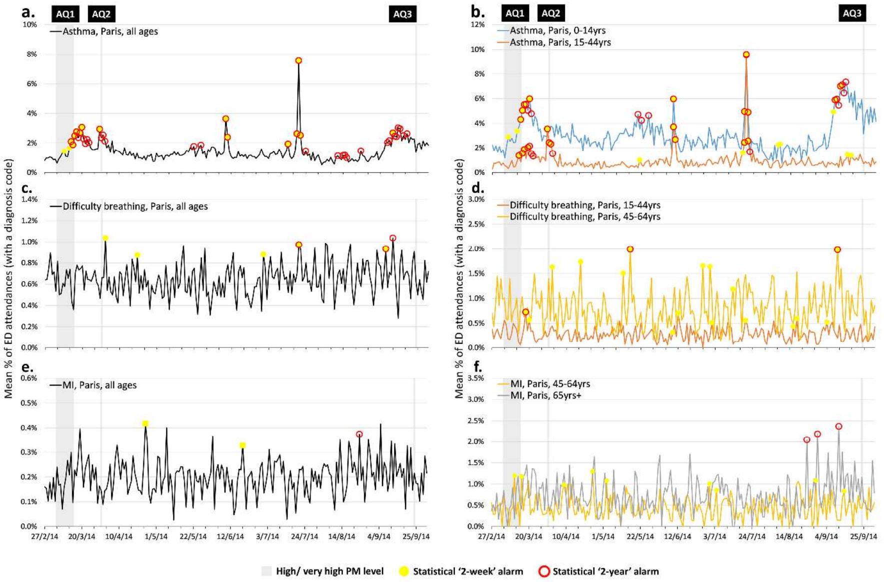

Figure 4: Daily percentages of Paris ED attendances for syndromic surveillance indicators of a. asthma all ages, b. asthma 0-14 and 15-44years, c. Difficulty breathing all ages, d. Difficulty breathing 15-44 and 45-64 years, e. Myocardial Ischaemia all ages and f. Myocardial ischaemia 45-64 and 65+ years, with statistical alarms, reported to OSCOUR®.

599x399mm (300 x 300 DPI)

STROBE Statement—checklist of items that should be included in reports of observational studies

	Item No	Recommendation
**Title and abstract**	1	(*a*) Indicate the study's design with a commonly used term in the title or the abstract<br><br>**A retrospective comparison of emergency department syndromic surveillance data during air pollution episodes across London and Paris in 2014 [p1]**
		(*b*) Provide in the abstract an informative and balanced summary of what was done and what was found<br><br>**Introduction**<br>Poor air quality (AQ) is a global public health issue and AQ events can span across countries. Using emergency department (ED) syndromic surveillance from England and France, we describe changes in human health indicators during periods of particularly poor AQ in London and Paris during 2014.<br>**Methods**<br>Using daily AQ data for 2014, we identified 3 periods of poor AQ affecting both London and Paris. Anonymised near real-time ED attendance syndromic surveillance data from EDs across England and France were used to monitor the health impact of poor AQ.<br>Using the routine English syndromic surveillance detection methods, increases in selected ED syndromic indicators (asthma, difficulty breathing and myocardial ischaemia), in total and by age, were identified and compared to periods of poor AQ in each city. Retrospective Wilcoxon-Mann-Whitney tests were used to identify significant increases in ED attendance data on days with (and up to 3 days following) poor AQ.<br>**Results**<br>Almost 1.5 million ED attendances were recorded during the study period (27/2/14-1/10/14). Significant increases in ED attendances for asthma were identified around periods of poor AQ in both cities, especially in children (0-14yrs). Some variation was seen in Paris with a rapid increase during the first AQ period in asthma attendances amongst children (0-14yrs), whereas during the second period the increase was greater in adults.<br>**Discussion**<br>This work demonstrates the public health value of real-time syndromic surveillance in response to air pollution incidents, and the potential for further cross-border harmonisation to provide Europe-wide early alerting to health impacts. **[p2/3]**
**Introduction**		
Background/rationale	2	Explain the scientific background and rationale for the investigation being reported<br><br>Air pollution has negative impacts on human health. Short term exposure to poor air quality can affect lung function, including exacerbating asthma symptoms, and is associated with other acute deteriorations in respiratory and cardiovascular health [1]. Similar health effects have also been reported due to long term exposure, with exposure to ambient air pollution associated with lung cancer and chronic respiratory and cardiovascular conditions [1]. In addition to illness within the community and increased need for health care, air pollution is also associated with increased

For peer review only

		mortality, with an estimated 4.7% of deaths in the England attributed to air pollution [2] and 9% of deaths in France attributed to PM2.5 [3]. **[p4]**
Objectives	3	State specific objectives, including any prespecified hypotheses<br><br>During March and early April 2014 there was a period of widespread poor AQ across Europe. In particular, the urban conurbations of London (England) and Paris (France) were affected by high temperatures, Saharan dust and industrial emissions, resulting in widespread media attention [17-19]. Here, we use routine emergency department (ED) syndromic surveillance data collected across London and Paris during poor AQ periods throughout 2014 to investigate the compatibility of the two countries' ED syndromic surveillance systems and estimate describe the public health impact and associated short-term changes in health care seeking behaviour for selected respiratory and cardiac syndromes across different age groups. **[p5]**
**Methods**		
Study design	4	Present key elements of study design early in the paper<br><br>Here, we use routine emergency department (ED) syndromic surveillance data collected across London and Paris during poor AQ periods throughout 2014 to investigate the compatibility of the two countries' ED syndromic surveillance systems and estimate describe the public health impact and associated short-term changes in health care seeking behaviour for selected respiratory and cardiac syndromes across different age groups. **[p5]**
Setting	5	Describe the setting, locations, and relevant dates, including periods of recruitment, exposure, follow-up, and data collection<br><br>The area studied here has been limited to London and the whole Paris region (Île-de-France), rather than a country level. **[p6]**<br><br>This investigation included EDSSS participating EDs within the London PHE Centre, which all fall within central London. **[p7]**<br><br>Aggregated, anonymised daily data for the Paris region were made available for this analysis. **[p7]**<br><br>An overall study period was defined as 27 February 2014 – 1 October 2014 (216 days), to encompass each period where poor AQ occurred in both London and Paris, including 7 days before and after the first and final AQ events identified (table 23). **[p10]**
Participants	6	(*a*) *Cohort study*—Give the eligibility criteria, and the sources and methods of selection of participants. Describe methods of follow-up<br>*Case-control study*—Give the eligibility criteria, and the sources and methods of case ascertainment and control selection. Give the rationale for the choice of cases and controls<br>*Cross-sectional study*—Give the eligibility criteria, and the sources and methods of selection of participants<br><br>The Emergency Department Syndromic Surveillance System (EDSSS), is a sentinel ED system coordinated by Public Health England (PHE), collecting anonymised data from participating EDs on a daily basis (data for the previous day 00:00 to 23:59 are

For peer review only - http://bmjopen.bmj.com/site/about/guidelines.xhtml

		transferred to PHE the following morning) [23]. Diagnosis coding in EDs in England was not standardised at the time of this investigation. Each ED had a list of diagnosis terms created locally which was available for selection in the patient attendance record. These diagnostic terms have associated codes linked to them with each ED using one of three codesets: Commissioning Data Set Accident and Emergency Diagnosis Tables[24], ICD-10 [25] or Snomed CT [26]. EDs eligible for inclusion in this study were defined as those reporting using ICD-10 [25] or Snomed CT [26] diagnosis coding systems which provide the level of detail required for the identification of the indicators of interest. **[p6/7]**<br><br>The French national ED syndromic surveillance system collects daily data from the Organisation de la Surveillance COordonnée des URgences (OSCOUR®) network of EDs, coordinated by Santé Publique France [27] (again, data for the previous day 00:00 to 23:59 are transferred and analysed the following morning, though OSCOUR® does allow for updates and delayed reporting, with around 15% of EDs reporting in the following 2 days[28]) [27]. All EDs reporting to OSCOUR® use ICD-10 for the coding of diagnoses selected in the patient attendance record [28]. **[p6/7]**
		(*b*) *Cohort study*—For matched studies, give matching criteria and number of exposed and unexposed<br>*Case-control study*—For matched studies, give matching criteria and the number of controls per case
Variables	7	Clearly define all outcomes, exposures, predictors, potential confounders, and effect modifiers. Give diagnostic criteria, if applicable<br><br>In England, the Department for Environment, Food and Rural Affairs monitors and reports on levels of air pollution using monitoring stations and provides health advice using the Daily Air Quality Index (DAQI) [9]. Air quality in the Paris region is monitored by Airparif and reported using the Citeair index [20]. **[p6]**<br><br>Syndromic indicators (asthma, difficulty breathing and myocardial ischaemia (MI) (table 1)) were selected from the comparable indicators already created for each system, based on clinical knowledge and experience of the potential health effects linked to air pollution and those used in previous syndromic surveillance work. **[p7]**
Data sources/ measurement	8*	For each variable of interest, give sources of data and details of methods of assessment (measurement). Describe comparability of assessment methods if there is more than one group<br><br>Both DAQI and Citeair systems monitor and report on multiple pollutants, however each index is reported using different methodology. Therefore the daily pollution levels across both London and Paris were standardised here, using the reported levels of particulate matter (PM2.5 and PM10). The city wide average value for each PM on each calendar day was calculated as a mean of the maximum values reported for each monitoring station on that day, in that city [21, 22]. Periods of poor AQ were then defined as those when either the calculated PM2.5 or PM10 average value corresponded to the DAQI index levels of 7-10, which are classified as 'high', to 'very high' (PM2.5 >=54 µg/m³ or PM10 >=76 µg/m³). At these levels people, including those with no pre-existing medical conditions, are advised to consider reducing their activity levels, particularly outdoors [8]. **[p6]**

For peer review only - http://bmjopen.bmj.com/site/about/guidelines.xhtml

		These syndromic surveillance indicators, which are routinely used in both EDSSS and OSCOUR® are an aggregation relevant diagnostic codes representing similar diagnostic terms and recorded in the patient record. Though 'diagnostic' information these diagnoses have potentially been made before any final confirmation and may be based on the symptoms presented, with no level of certainty indicated. The overall asthma and MI indicator groupings were very similar in each system, with the terms included all describing either asthma or myocardial ischaemic conditions. Differences were found in non-asthma difficulty breathing type indicators, where EDSSS included symptomatic wheeze/ difficulty breathing type diagnoses and OSCOUR® included dyspnoea/ respiratory failure diagnoses (table 2). Please note: not every code listed was reported by – or even available for selection – from every ED. More relevant codes may exist for each indicator than described here, however only codes reported to EDSSS/ OSCOUR® in this study are included). **[p7/8]**
Bias	9	Describe any efforts to address potential sources of bias<br>Not applicable
Study size	10	Explain how the study size was arrived at<br>An overall study period was defined as 27 February 2014 – 1 October 2014 (216 days). **[p10]**<br>Over the study period 1,436,163 ED attendances were recorded across both London and Paris. **[p11]**
Quantitative variables	11	Explain how quantitative variables were handled in the analyses. If applicable, describe which groupings were chosen and why<br><br>Both DAQI and Citeair systems monitor and report on multiple pollutants, however each index is reported using different methodology. Therefore the daily pollution levels across both London and Paris were standardised here, using the reported levels of particulate matter (PM2.5 and PM10). The city wide average value for each PM on each calendar day was calculated as a mean of the maximum values reported for each monitoring station on that day, in that city [21, 22]. Periods of poor AQ were then defined as those when either the calculated PM2.5 or PM10 average value corresponded to the DAQI index levels of 7-10, which are classified as 'high', to 'very high' (PM2.5 >=54 µg/m³ or PM10 >=76 µg/m³). At these levels people, including those with no pre-existing medical conditions, are advised to consider reducing their activity levels, particularly outdoors [8]. **[p6]**<br><br>Syndromic indicators (asthma, difficulty breathing and myocardial ischaemia (MI) (table 1)) were selected from the comparable indicators already created for each system, based on clinical knowledge and experience of the potential health effects linked to air pollution and those used in previous syndromic surveillance work. **[p7]**<br><br>For each syndromic surveillance system, attendances were aggregated by age group defined as 0-14, 15-44, 45-64 and 65 years and over. **[p8]**
Statistical methods	12	(*a*) Describe all statistical methods, including those used to control for confounding<br><br>We applied the routine syndromic surveillance statistical detection algorithm from England: the RAMMIE method (Rising Activity, Multi-level Mixed effects Indicator Emphasis [29]). **[p8]**

For peer review only - http://bmjopen.bmj.com/site/about/guidelines.xhtml

		RAMMIE routinely allows for the prioritisation of alarms to facilitate the identification of significant activity, however, this function was not used here to ensure that all statistically significant activity was identified, and not just those signals prioritised by RAMMIE. **[p9]**<br><br>In addition to the RAMMIE analysis, the Wilcoxon-Mann-Whitney non-parametric test was used to test for significant differences in the syndromic indicators during the 2014 study period, by age group between those days with a poor AQ and those without. To allow for the possibility of a delayed response, separate analyses were conducted incorporating lags of one to three days following a day of poor AQ. **[p9]**
		(*b*) Describe any methods used to examine subgroups and interactions<br><br>RAMMIE was applied to both English and French ED data, including to age specific data. **[p8/9]**<br><br>In addition to the RAMMIE analysis, the Wilcoxon-Mann-Whitney non-parametric test was used to test for significant differences in the syndromic indicators during the 2014 study period, by age group between those days with a poor AQ and those without. **[p9]**
		(*c*) Explain how missing data were addressed
		(*d*) *Cohort study*—If applicable, explain how loss to follow-up was addressed<br>*Case-control study*—If applicable, explain how matching of cases and controls was addressed<br>*Cross-sectional study*—If applicable, describe analytical methods taking account of sampling strategy<br><br>Not applicable
		(*e*) Describe any sensitivity analyses<br>Not applicable
**Results**		
Participants	13*	(a) Report numbers of individuals at each stage of study—eg numbers potentially eligible, examined for eligibility, confirmed eligible, included in the study, completing follow-up, and analysed<br>Not applicable
		(b) Give reasons for non-participation at each stage<br>Not applicable
		(c) Consider use of a flow diagram<br>Not applicable
Descriptive data	14*	(a) Give characteristics of study participants (eg demographic, clinical, social) and information on exposures and potential confounders<br>Not applicable
		(b) Indicate number of participants with missing data for each variable of interest<br>Not applicable
		(c) *Cohort study*—Summarise follow-up time (eg, average and total amount)<br>Not applicable
Outcome data	15*	*Cohort study*—Report numbers of outcome events or summary measures over time<br>Not applicable

For peer review only

		Case-control study—Report numbers in each exposure category, or summary measures of exposure<br>Not applicable
		Cross-sectional study—Report numbers of outcome events or summary measures<br>During 2014, several periods of poor AQ were identified where the 'high' or 'very high' air pollution thresholds for particulate matter (PM2.5 and/or PM10) had been breached in both London and Paris (figure 1). Periods of poor air quality in Paris were generally observed to be of a longer duration and with higher DAQI levels than in London, though more individual days of poor AQ were identified in London. Two main periods of poor AQ overlapped in these cities in mid-March (AQ1, the largest event in both locations and where transboundary dust from the Sahara contributed to the makeup of the particulate matter fraction [14]) and early April (AQ2, mainly in London, though a 1 day PM10 spike in Paris), with a third, less severe period during September occurring in both cities within a 7 day period (AQ3) (table 3). **[p10]**<br>Over the study period 1,436,163 ED attendances were recorded across both London and Paris (table 4). Total attendances were higher in Paris (1,163,353; from 58 EDs) than London (272,810; from 5 EDs, 3 using ICD-10, 2 using SnomedCT). A comparable level of diagnosis coding was included in each city with 79% of London attendances and 72% of Paris attendances including a clinical diagnosis code. **[p11]**
Main results	16	(*a*) Give unadjusted estimates and, if applicable, confounder-adjusted estimates and their precision (eg, 95% confidence interval). Make clear which confounders were adjusted for and why they were included<br><br>Results of RAMMIE testing are given in figures 3 & 4 and Wilcoxon-Mann-Whitney testing are in table 5, and these are described in the text **[p12-14]**:<br><br>Small increases in asthma attendances (all ages) in London EDs were observed following AQ1 (figure 3a). ED asthma attendances continued to increase during and immediately following AQ2. RAMMIE 2-week alarms were reported for the increases in asthma (all ages) immediately following AQ1 in London, indicating an attendance level higher than the previous 2 weeks. **[p12]**<br><br>Clear increases in ED attendances (all ages) for asthma occurred during both AQ1 and AQ2 in Paris (figure 4a). These increases were detected by RAMMIE as statistically significant in comparison to previous years (2-year alarm), as well as compared to the preceding 2 weeks (2-week alarm). **[p13]**<br><br>Wilcoxon-Mann-Whitney test results provide further evidence, alongside the descriptive epidemiology and RAMMIE results, that there is a strong associationbetween days of poor AQ and asthma attendances all ages and particularly in children 0-14 years (table 5)**[p14]**
		(*b*) Report category boundaries when continuous variables were categorized<br>Not applicable
		(*c*) If relevant, consider translating estimates of relative risk into absolute risk for a meaningful time period<br>Not applicable
Other analyses	17	Report other analyses done—eg analyses of subgroups and interactions, and sensitivity analyses<br><br>The observed increase of asthma attendances during the AQ2 episode in London was most

For peer review only - http://bmjopen.bmj.com/site/about/guidelines.xhtml

evident in children aged 0-14 years, and young adults (15-44 years) with each age group reaching a peak in attendances 1 to 2 days later (figure 3b). Asthma attendances for older adults showed no evidence of increase around periods of poor AQ (data not shown). **[p12]**

Clear increases in ED attendances (all ages) for asthma occurred during both AQ1 and AQ2 in Paris (figure 4a). These increases were detected by RAMMIE as statistically significant in comparison to previous years (2-year alarm), as well as compared to the preceding 2 weeks (2-week alarm). However, when broken down by age, the increase in asthma attendances in the 0-14 years age group occurred during AQ1, but not AQ2; while asthma attendances in young adults (15-44yrs) were greater during AQ2 than AQ1. No statistical alarms were observed for asthma in children around AQ2, though they were present for young adults (figure 4b). **[p13]**

**Discussion**		
Key results	18	Summarise key results with reference to study objectives<br><br>We used two national ED syndromic surveillance systems to describe and compare the short-term changes in ED indicators during periods of poor AQ in two European capital cities. The AQ events reported here in Paris and London were related to the same pollutants (PM2.5/ PM10), and were very similar in terms of the dates and duration, and changes in public health outcomes in terms of ED attendances.<br><br>The most sensitive ED indicator during periods of poor AQ was asthma, with the impact most apparent up to 3 days after a day of poor AQ. The breakdown of attendances by age group revealed some differences, with the strongest associations overall seen between poor AQ and asthma attendances in children. **[p15]**
Limitations	19	Discuss limitations of the study, taking into account sources of potential bias or imprecision. Discuss both direction and magnitude of any potential bias<br><br>1. The use of percentage or ED visits (with a diagnosis code), as an indication of ED attendances (rather than actual numbers), as reported here may be impacted by the overall levels of ED attendances (and levels of diagnostic coding) on any one day **[p17]**<br><br>2. A limitation of the statistical methods used here is that the occurrence of previous events (e.g. poor AQ or weather systems) influencing the indicators were not identified or removed from the 2 years of historical data used as RAMMIE training data **[p18]**<br><br>3. This study focussed solely on particulate matter, though other pollutants impact on human health **[p18]**<br><br>4. The impact of health warnings and media reporting associated with actual and predicted periods of poor AQ could not be controlled for **[p18]**<br><br>5. It is important here to underline that variations of near real-time indicators are not easy to attribute directly to poor AQ. An absence of short term variation (e.g. MI in this study) cannot not be interpreted as a total lack of any longer term impact. Similarly, the identification of a significant increase in syndromic indicators reported here (e.g. asthma) has not formally accounted for other

For peer review only - http://bmjopen.bmj.com/site/about/guidelines.xhtml

		associated factors such as climatic conditions (e.g. weather and allergens) or viral circulation **[p19]**
Interpretation	20	Give a cautious overall interpretation of results considering objectives, limitations, multiplicity of analyses, results from similar studies, and other relevant evidence<br><br>The most sensitive ED indicator during periods of poor AQ was asthma, with the impact most apparent up to 3 days after a day of poor AQ. The breakdown of attendances by age group revealed some differences, with the strongest associations overall seen between poor AQ and asthma attendances in children. This finding was consistent with previous studies which have shown children to be more susceptible to exacerbation of asthma symptoms requiring health care in association with air pollution [31].**[p15]**<br><br>The investigation of individual AQ incidents demonstrated the potential for differing levels of impact on different age groups at different times. Though generally children were most affected by AQ, a large increase in adult asthma attendances was observed during and immediately following AQ2 in both London and Paris. Within England this increase in attendances around AQ2 has previously been described [32]. **[p15]**
Generalisability	21	Discuss the generalisability (external validity) of the study results<br><br>This work shows the potential of real-time syndromic surveillance to enhance the public health response to air pollution incidents, even if real-time changes observed through syndromic surveillance data cannot be absolutely related to air pollution. **[p19]**
**Other information**		
Funding	22	Give the source of funding and the role of the funders for the present study and, if applicable, for the original study on which the present article is based<br>Not applicable

*Give information separately for cases and controls in case-control studies and, if applicable, for exposed and unexposed groups in cohort and cross-sectional studies.

Note: An Explanation and Elaboration article discusses each checklist item and gives methodological background and published examples of transparent reporting. The STROBE checklist is best used in conjunction with this article (freely available on the Web sites of PLoS Medicine at http://www.plosmedicine.org/, Annals of Internal Medicine at http://www.annals.org/, and Epidemiology at http://www.epidem.com/). Information on the STROBE Initiative is available at www.strobe-statement.org.

For peer review only

For peer review only - http://bmjopen.bmj.com/site/about/guidelines.xhtml
